# Insulin-like growth factor-binding protein-7 (IGFBP7) links senescence to heart failure

**DOI:** 10.1038/s44161-022-00181-y

**Published:** 2022-12-22

**Authors:** Liyong Zhang, David Smyth, Mohammad Al-Khalaf, Alice Blet, Qiujiang Du, Jordan Bernick, Michael Gong, Xu Chi, Yena Oh, Malaika Roba-Oshin, Elizabeth Coletta, Michel Feletou, Anthony O. Gramolini, Kyoung-Han Kim, Thais Coutinho, James L. Januzzi, Benoit Tyl, Andre Ziegler, Peter P. Liu

**Affiliations:** 1 University of Ottawa Heart Institute, Ottawa, ON Canada; 2 Cellular and Molecular Medicine, University of Ottawa, Ottawa, ON Canada; 3 Cardiovascular and Metabolic Disease Center for Therapeutic Innovation, Institut de Recherches Internationales Servier, Suresnes, France; 4 Ted Rogers Centre for Heart Research, University of Toronto, Toronto, ON Canada; 5 Department of Medicine, University of Ottawa, Ottawa, ON Canada; 6 Cardiology Division, Massachusetts General Hospital, Harvard Medical School, Baim Institute for Clinical Research, Boston, MA USA; 7 Roche Diagnostics International, Ltd., Rotkreuz, Switzerland

**Keywords:** Ageing, Heart failure, Heart failure, Prognostic markers

## Abstract

Heart failure (HF) is a rising global cardiovascular epidemic driven by aging and chronic inflammation. As elderly populations continue to increase, precision treatments for age-related cardiac decline are urgently needed. Here we report that cardiac and blood expression of IGFBP7 is robustly increased in patients with chronic HF and in an HF mouse model. In a pressure overload mouse HF model, *Igfbp7* deficiency attenuated cardiac dysfunction by reducing cardiac inflammatory injury, tissue fibrosis and cellular senescence. IGFBP7 promoted cardiac senescence by stimulating IGF-1R/IRS/AKT-dependent suppression of FOXO3a, preventing DNA repair and reactive oxygen species (ROS) detoxification, thereby accelerating the progression of HF. In vivo, AAV9-shRNA-mediated cardiac myocyte *Igfbp7* knockdown indicated that myocardial IGFBP7 directly regulates pathological cardiac remodeling. Moreover, antibody-mediated IGFBP7 neutralization in vivo reversed IGFBP7-induced suppression of FOXO3a, restored DNA repair and ROS detoxification signals and attenuated pressure-overload-induced HF in mice. Consequently, selectively targeting IGFBP7-regulated senescence pathways may have broad therapeutic potential for HF.

## Abstract (web summary)

Zhang et al. show increased blood concentration of IGFBP7 in patients and a mouse model with heart failure (HF). IGFBP7 promotes cardiac senescence by stimulating IGF-1R/IRS/AKT-dependent suppression of FOXO3a, and Igfbp7 deficiency or inhibition attenuates cardiac dysfunction in a pressure overload mouse HF model, suggesting that therapeutic targeting of IGFBP7 might be promising for the treatment of HF.

## Main

Heart failure (HF) is a clinical syndrome associated with high mortality and poor quality of life, with a prevalence that increases substantially in the elderly^1^. During aging, deterioration in cardiac structure and function, along with reduced capacity to respond to myocardial stress, injury and inflammation, leads to increased susceptibility to HF^2–4^. The myocardial processes described in HF include excessive oxidative stress and chronic low-grade inflammation and accelerated cardiovascular senescence^5,6^. Cellular senescence, a permanent state of cell cycle arrest in response to various stressors, has emerged as a fundamental contributor to aging and chronological age-related disease^7^. Chronological age-related disorders, including HF and atherosclerosis, link with an increased accumulation of senescent cells in the heart and vessels^8^. Senescent cells become pathogenic by releasing a variety of pro-inflammatory and matrix-degrading molecules, known as the senescence-associated secretory phenotype (SASP), mediating chronic low-grade inflammation and tissue remodeling, causing a progressive functional deterioration and ultimately heart dysfunction^9,10^. Despite a considerable increase in research on the causes and pathophysiology of chronological age-related HF, strategies for prevention or targeting the cause remain elusive. Furthermore, cardiovascular senescence has, to date, been only sparingly studied.

In our laboratory, we have been investigating potential novel mechanisms leading to chronological age-related HF through clinical biomarker discovery and validation. One of the most robust biomarkers identified to date is IGFBP7 (refs. ^11,12^). Based on initial clinical data, IGFBP7 has been suggested, in a recent consensus statement on biomarkers by the American Heart Association, as an important biomarker for the prognosis and diagnosis of HF^13^. Subsequent validations in independent cohorts of chronic HF have demonstrated, in the National Institutes of Health RELAX HF trial, that IGFBP7 is a biomarker of diastolic dysfunction and functional capacity^14–17^. IGFBP7 levels correlated with diastolic filling and left atrial (LA) dilation in patients with HF, and treatment with sacubitril–valsartan decreased IGFBP7 levels^18^. IGFBP7 is as effective as N-terminal pro-brain natriuretic peptide (NT-proBNP) but independent from NT-proBNP in providing diagnosis and prognosis in patients presenting with dyspnea and acute HF where elevated concentrations of IGFBP7 predict major adverse cardiovascular events and are correlated with disease severity and structural abnormalities of the heart^19,20^. Importantly, plasma IGFBP7 level independently predicted left ventricular hypertrophy and cardiac remodeling on echocardiography but also powerfully predicted all-cause mortality in a 10-year community aging study of patients 65 years of age or older^21^. In addition to its prognostic and diagnostic value, the rising plasma IGFBP7 concentrations also predicted renal and cardiac events among participants with type 2 diabetes and high cardiovascular risks in the recent canagliflozin (a SGLT2 inhibitor) cardiovascular assessment study^22^. A rising value of IGFBP7 was even more predictive for worse outcome than an elevated level at baseline, which indicated that IGFBP7 levels are the best predictors of SGLT2 inhibitor efficacy, the latter an agent with broad cardiorenal and metabolic protective properties and in turn prevention of HF^22^.

IGFBP7 inhibits cell proliferation through G1 phase cell cycle arrest and is a prominent protein member of the SASP^23–25^. Excessive SASP activation and inflammation contribute to accelerated cellular aging, tissue degeneration and organ dysfunction^26^. This process is likely active in HF as prevalent upstream morbidities, including hypertension or diabetes, subsequently trigger multiple stresses, such as DNA damage, oxidative stress and innate immune inflammation, all of which contribute to SASP production and cellular senescence^26^. Despite its predictive value and strong association with clinical outcomes, whether elevated circulating IGFBP7 is merely a consequence of or a critical contributor to HF pathogenesis remains unknown. The goal of the present study was to investigate the relevant molecular mechanisms of IGFBP7 in the development and progression of HF. Using a combination of molecular and biochemical analyses of clinical samples, knockout models, in vivo *Igfbp7* knockdown in cardiac myocytes and monoclonal antibody (mAb)-mediated blockade, this study reveals that IGFBP7 directly regulates pathological cardiac remodeling, senescence and fibrosis by suppressing the FOXO3a-mediated pro-longevity pathway, thereby accelerating the progression of HF. Eventually, such IGFBP7-dependent pathways might not only be prognostic, predictive and diagnostic but also a therapeutic target for HF.

## Results

### Elevated plasma IGFBP7 is correlated with chronic inflammation

**Fig. 1:**
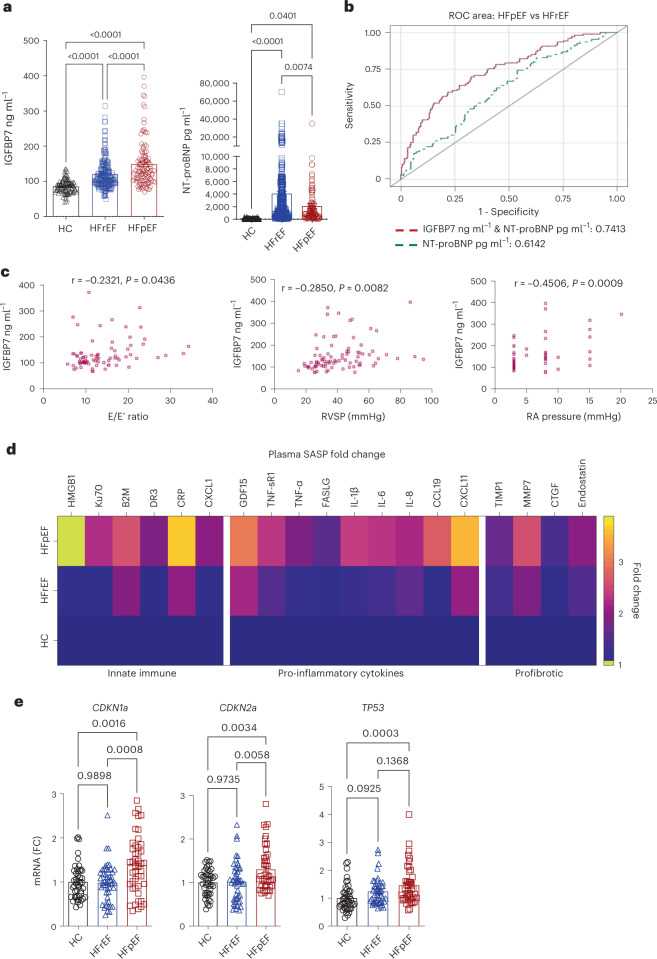
Elevated plasma IGFBP in HF is correlated with chronic inflammation and accelerated cellular senescence. **a**, IGFBP7 and NT-proBNP protein concentrations in plasma samples of patients with HFpEF (*n* = 106), patients with HFrEF (*n* = 207) and controls (*n* = 98) are presented as mean ± s.e.m. in scatter plots; one-way ANOVA with Bonferroni correction for multiple comparisons was used to calculate *P* value. **b**, ROC curve shows that the addition of IGFBP7 to NT-proBNP II values significantly improved the diagnostic performance in the discrimination of HFpEF from HFrEF. **c**, Correlation analysis revealed that increased plasma IGFBP7 level in HFpEF correlates with decreased diastolic function of the heart. **d**, Heat map shows changes of SASP proteins in plasma samples of HFpEF (*n* = 13), HFrEF (*n* = 26) and controls (HC) (*n* = 9); protein expression is shown as fold change against control group. *P* < 0.05–0.0001 for all panels. **e**, Relative gene expression of key senescence markers in whole blood samples; *CDKN2A* (*p16*), *CDKN1A* (*p21*) and *TP53* (*p53*) are shown as fold changes against HC (*n* = 15 per group). Error bars represent s.e.m. One-way ANOVA with Bonferroni correction for multiple comparisons was used to calculate *P* values. RA, right atrial; RVSP, right ventricular systolic pressure.

**Extended Data Fig. 1:**
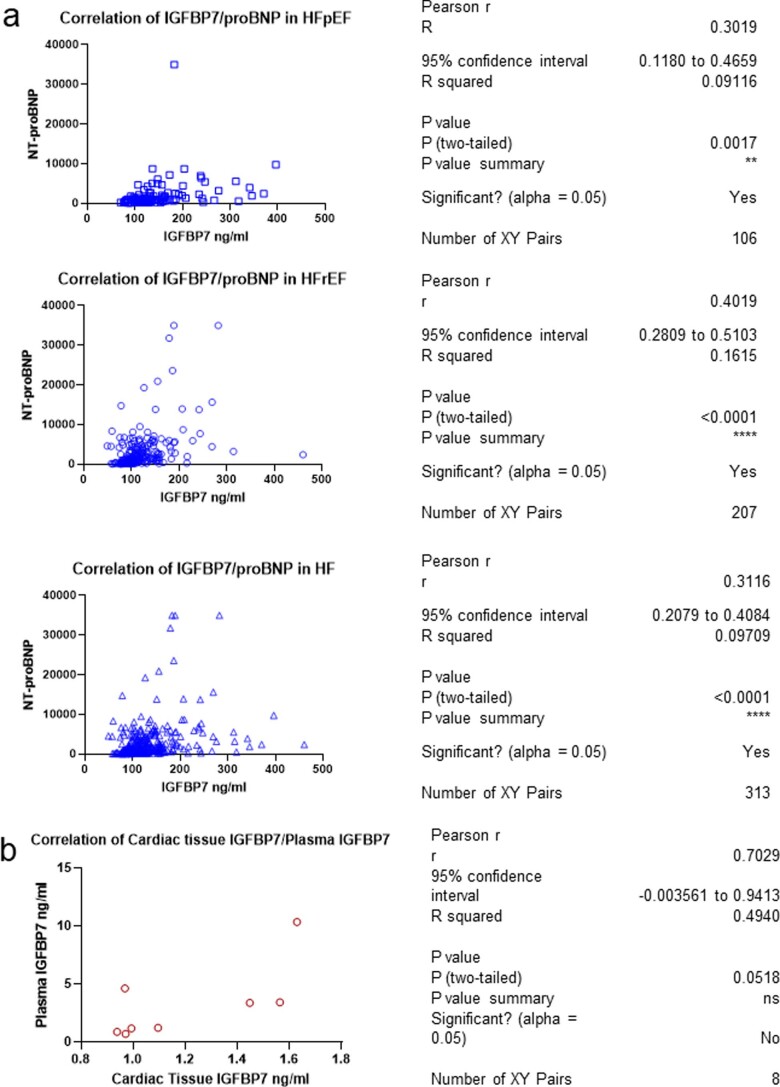
IGFBP7 is independently informative of processes contributing to HF from pro-BNP. **(a)** Correlation analysis revealed there is poor correlation between the level of IGFBP7 and NT-proBNP, whether in the HFpEF, HFrEF or the overall HF population (r value between 0.30 and 0.40). **(b)** Correlation analysis revealed there is reasonable correlation between myocardial levels of IGFBP7 and circulating levels of IGFBP7, with r value of 0.7. Correlation analyses were performed with GraphPad Prism 9 and the Pearson registration analysis were summarized in the right panel next to each plot.

Plasma IGFBP7 protein concentrations were measured using Roche Cobas Elecsys assays in a chronic HF cohort that was sub-grouped into heart failure with preserved ejection fraction (HFpEF) (left ventricular ejection fraction (LVEF) > 50%) (*n* = 106) and heart failure with reduced ejection fraction (HFrEF) (LVEF < 40%) (*n* = 207), according to broadly accepted criteria, and non-HF controls (*n* = 98). NT-proBNP, a well-established biomarker for HF^27^, was also measured as a reference standard. The clinical characteristics of the groups, including demographics, echocardiographic and medical history, are shown in Extended Data Tables 1 and 2. Consistent with previous studies, elevated IGFBP7 and NT-proBNP were readily detected in all patients with HF compared to controls, and this elevation showed marked segregation between HFpEF and HFrEF patient cohorts. IFGBP7 plasma levels were significantly higher in patients with HEpEF (LVEF > 50%) in comparison to HFrEF (LVEF < 40%); in contrast, NT-proBNP was significantly higher in HFrEF (Fig. 1a and Extended Data Table 1). There was poor correlation between the level of IGFBP7 and NT-proBNP, whether in the HFpEF, HFrEF or overall HF population (*r* value between 0.30 and 0.040) (Extended Data Fig. 1a). This indicates that IGFBP7 is independently informative of processes contributing to HF, and does not duplicate NT-proBNP, but appears to complement the information derived from NT-proBNP. Receiver operating characteristic (ROC) analysis showed that the addition of IGFBP7 to NT-proBNP values significantly improved the diagnostic performance in the discrimination of HEpEF from HFrEF (up from 61% to 74%) (Fig. 1b). Echocardiographic analysis revealed that declined cardiac diastolic function is a potential confounding factor of the elevated plasma IGFBP7 in patients with HFpEF (Fig. 1c). Our understanding of IGFBP7 association with senescence led us to investigate whether other SASP proteins were elevated in the plasma of patients with HF. Plasma samples from a group of HFpEF patients (*n* = 13), a group of HFrEF patients (*n* = 26) and controls (*n* = 9) from the same cohort as above were assayed by aptamer-based SomaScan proteomics. We observed that a cluster of SASP proteins, regulators of innate immunity, pro-inflammatory and profibrotic cytokines and chemokines, were significantly elevated in HF, including a marked increase in HFpEF (Fig. 1d). The clinical characteristics of the groups are shown in Supplementary Table 1. In addition, RT–qPCR analysis revealed that gene expression of key senescence markers *CDKN2A* (*p16*), *CDKN1A* (*p21*) and *TP53* (*p53*) was significantly elevated in blood samples of patients with HFpEF (Fig. 1e). The clinical characteristics of the groups are shown in Supplementary Table 2, collectively indicating that elevated plasma IGFBP7 in HF was correlated with chronic inflammation and accelerated cellular senescence.

### Cardiac expression of IGFBP7 is increased in HF

**Fig. 2:**
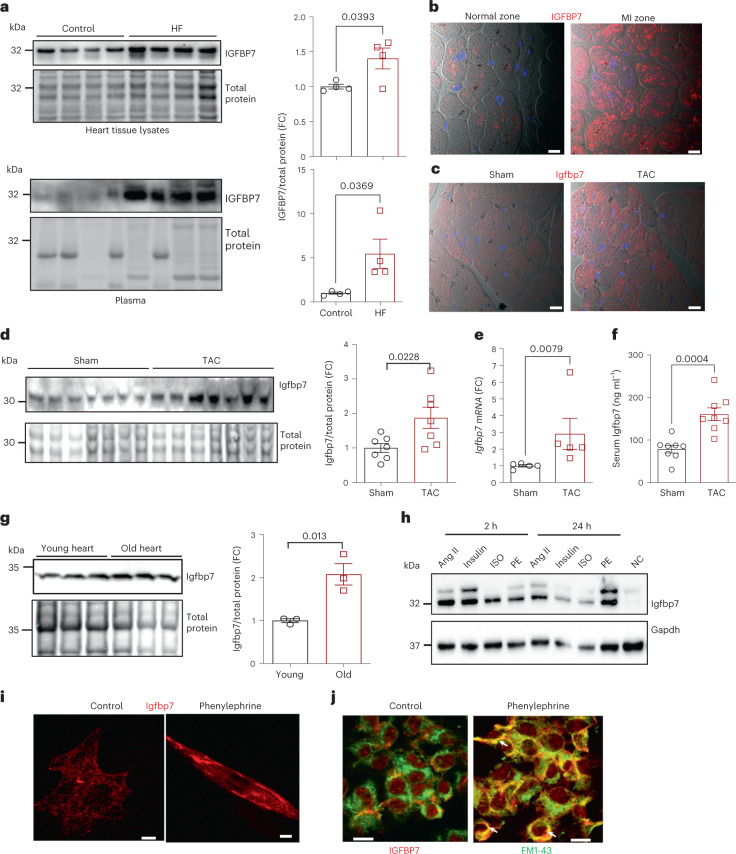
Cardiac expression of IGFBP7 is increased in patients with HF and mouse models of HF. **a**, Representative immunoblotting and quantification show that IGFBP7 protein expression was markedly increased in both heart tissue lysates and plasma samples of patients with HF compared to non-HF controls; expression levels were normalized to total proteinn (*n* = 4 per group). **b**,**c**, Representative confocal microscopy images examined over two independent experiments showing significantly increased IGFBP7 in cardiomyocytes of infarct zone of a patient who suffered from HF after myocardial infarction (MI) compared to normal zone of the same patient (**b**) and elevated Igfbp7 staining in cardiomyocytes of TAC mouse heart compared to sham control 8 weeks after surgery (**c**). Heart sections were probed with anti-IGFBP7 antibody and visualized by staining with Alexa Fluor 555 secondary antibody (red), and nuclei were stained with DAPI (blue). In all images, scale bars are 30 μm. **d**, Representative immunoblotting and quantification show that Igfbp7 protein expression was markedly increased in TAC heart compared to sham heart 8 weeks after surgery; expression levels were normalized to total protein (*n* = 7 per group). **e,** Relative *Igfbp7* gene expression in TAC and sham mouse hearts is shown as fold changes against sham control (*n* = 5 per group). **f**, Elevated serum Igfbp7 was also evidenced in TAC mouse measured by ELISA 8 weeks after TAC (*n* = 8 per group). **g**, Representative immunoblots and quantification show that Igfbp7 protein expression is increased in aged C57BL/6 mouse (24-month-old) heart compared to young (3-month-old) heart; relative Igfbp7 protein level is shown as fold changes against young (*n* = 3 per group). **h**, Representative immunoblots show that Igfbp7 protein was significantly upregulated in neonatal rat cardiomyocytes (rNCMs) treated with various hypertrophic stimuli in vitro. Gapdh was used as loading control. **i**,**j**, Representative confocal microscopy images showing, upon exposure to cardiac stressors, upregulated Igfbp7 (red) relocated toward the plasma membrane in rNCMs (**i**) and IGFBP7 (red) co-localized with vesicular structures (green) that labeled with FM 1-43FX lipophilic styryl dye in AC16 hCMs in vitro (**j**). In all images, scale bars are 20 μm. In all panels, error bars represent s.e.m. Unpaired two-tailed *t-*tests were used to calculate *P* values. Source data

**Extended Data Fig. 2:**
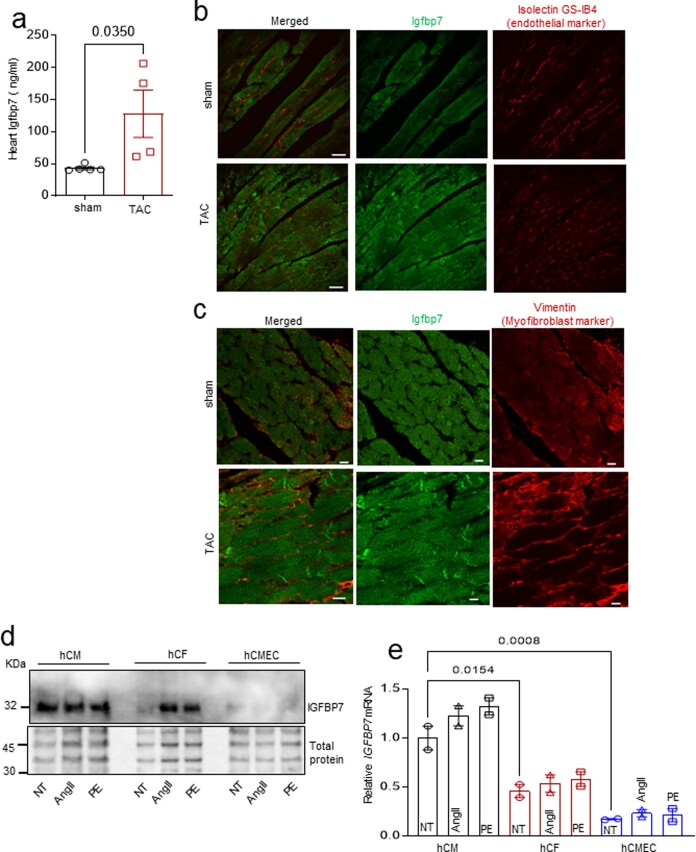
Hypertrophic stress induced IGFBP7 elevation were more evidenced in cardiac myocytes. **(a)** Igfbp7 protein expression was markedly increased in TAC heart compared with sham heart 8 weeks post-surgery measured by ELISA, shown as ng/ml of normalized whole heart lysate; n = 4 per group, unpaired two-tailed Student *t-test* was used to calculate *p* value. **(b–c)** Selected confocal microscope images of multiplex IHC with either **(b)** Igfbp7 and isolectin GS-IB4 (endothelial marker), or **(c)** Igfbp7 with Vimentin (myofibroblast marker) shows TAC induced increase of Igfbp7 protein was more evidenced in cardiac myocytes; less in cardiac microvascular endothelial cells and myofibroblasts. 8 weeks post sham or TAC mouse heart sections were stained with rabbit polyclonal anti-Igfbp7 antibody (abcam ab74169), visualized with Alexa Flour 647 chicken anti-rabbit IgG (ThermoFisher A21443), counter stained with Alexa Flour 568 conjugated Isolectin GS-IB4 (ThermoFisher, I21412) to visualize microvasculature or anti-vimentin antibody to visualize myofibroblast. Scale bar = 30 μm in panel **b**, and 20 μm in panel **c**. **(d)** Representative immunoblots showing increased IGFBP7 protein expression was more evidenced in primary human cardiac myocytes (hCM) than cardiac fibroblasts (hCF) and cardiac microvascular endothelial cells (hCMEC) (all from PromoCell). Total proteins were used as loading controls. **(e)** qRT–PCR analysis shows there is a significantly increased *Igfbp7 mRNA* expression in hCM compared with hCF and hCMEC. *IGFBP7* expression was shown as fold change again non-treated hCM. The data was from two independent treated cell culture experiment. Error bars represent s.e.m., one-way ANOVA with Bonferroni correction for multiple comparisons were used to calculate *p* values. Source data

To explore the possibility that elevated plasma IGFBP7 in patients with HF is due to stress response of the myocardium, heart tissue biopsies and plasma were collected from patients with chronic HF and non-HF controls. Indeed, significantly increased IGFBP7 protein expression was evident in both heart tissue lysates and plasma of patients with HF compared to non-HF controls by immunoblot (Fig. 2a). There is reasonable correlation between myocardial levels of IGFBP7 and circulating levels of IGFBP7, with *r* value of 0.7 (Extended Data Fig. 1b). This suggests that a significant portion of the circulating IGFBP7 reflects the production from the heart, thus underscoring the relevance of circulating IGFBP7 in delineating processes in the myocardium of patients with HF. IGFBP7 immunofluorescence staining of heart sections further demonstrated that the increase in IGFBP7 in the heart of HF was mainly due to increased IGFBP7 expression in cardiomyocytes (Fig. 2b). In addition, in a murine model of surgical transverse thoracic aortic constriction (TAC)-induced HF^28^, elevated Igfbp7 expression was also observed mainly in cardiomyocytes 8 weeks after surgery compared to sham-operated controls (Fig. 2c). This was further confirmed by observation of upregulated Igfbp7 protein (Fig. 2d and Extended Data Fig. 2a) and mRNA (Fig. 2e) expression in TAC hearts compared to sham-operated controls. Elevated serum Igfbp7 concentrations were also detected in TAC mice (Fig. 2f). To address if elevated Igfbp7 protein in TAC mouse heart is due to increased Igfbp7 in cardiomyocytes, multiplex immunofluorescent staining was used, which showed TAC-induced increase of Igfbp7 protein expression in cardiac myocytes—less in cardiac microvascular endothelial cells and myofibroblasts (Extended Data Fig. 2b,c).

As IGFBP7 is associated with senescence, we checked whether normal aging increased IGFBP7 levels in the heart. Heart tissue lysates showed markedly higher Igfbp7 protein expression in aged mice (24-month-old) compared to younger (3-month-old) controls (Fig. 2g). In vitro, Igfbp7 protein was significantly upregulated in rat neonatal cardiomyocytes treated with various hypertrophic stimuli (Fig. 2h). Next, we assayed hypertrophic stimuli angiotensin II (AngII) and phenylephrine (PE) treatment effect upon three major types of human primary cardiac cell types: human cardiac myocytes (hCMs), human cardiac microvascular endothelial cells (HCMECs) and human cardiac fibroblasts (HCFs). Stimulation with hypertrophic stimuli increased IGFBP7 protein (Extended Data Fig. 2d) and mRNA (Extended Data Fig. 2e) in both hCMs and HCFs, and cardiac myocytes have the highest IGFBP7 expression. Immunofluorescence microscopy demonstrated that, in cardiac myocytes after exposure to PE, upregulated Igfbp7 relocated toward the plasma membrane (Fig. 2i) and co-localized with vesicular structures (Fig. 2j), suggesting that stressed cardiomyocytes release IGFBP7.

### IGFBP7 is a key regulator of pathological cardiac remodeling

**Fig. 3:**
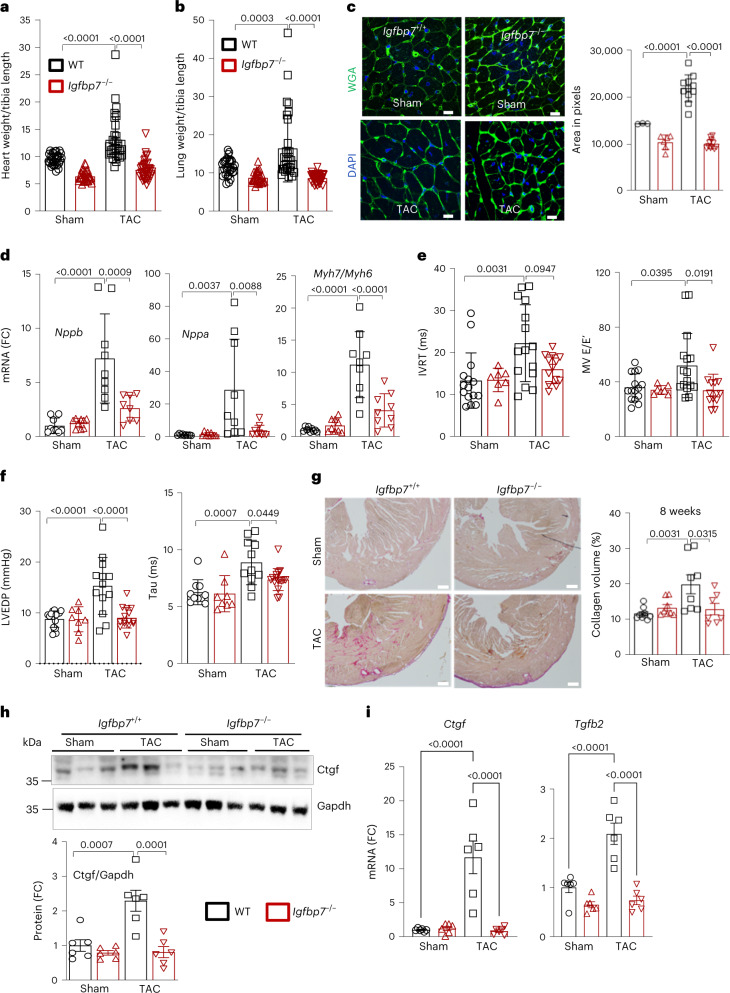
*Igfbp7* deficiency protects mice from pressure-overload-induced HF. *Igfbp7*^*−/−*^ and WT mice were subjected to TAC or sham surgery and analyzed 2–8 weeks after the operation. HW/TL (**a**) and LW/TL (**b**) ratio at 8 weeks (*n* = 30 WT and KO sham, *n* = 31 WT TAC and *n* = 38 KO TAC mice). **c**, Representative WGA staining of transverse heart (8 weeks) cross-sections showing myocyte cross-sectional area (scale bars, 20 μm) and quantitation (*n* = 3 WT sham, *n* = 11 WT TAC, *n* = 5 KO sham and *n* = 11 KO TAC images examined over slides from three mice of each group). **d**, Relative gene expression of *Nppb*, *Nppa* and *Myh7/Myh6* ratio in TAC and sham heart 8 weeks after surgery are shown as fold changes against WT sham group (*n* = 9 per group). **e**, Echocardiographic assessment of cardiac function at 8 weeks after surgery. IVRT and mitral E/E′ ratio measured by transmittal Doppler flow velocity are shown (*n* = 15 WT sham, *n* = 16 WT TAC, *n* = 7 KO sham and *n* = 13 KO TAC mice). **f**, Measurement of cardiac function by PV conductance catheterization 8 weeks after sham and TAC surgery. LVEDP and isovolumic relaxation constant (Tau) (Weiss model) are shown (*n* = 10 WT sham, *n* = 11 WT TAC, *n* = 8 KO sham and *n* = 16 KO TAC mice). **g**, Representative PSR staining for collagen of transverse heart cross-sections and quantization showing increased collagen deposition in WT TAC heart. This is much attenuated by Igfbp7 deficiency. Scale bars, 500 μm. *n* = 10 WT sham, *n* = 8 WT TAC, *n* = 8 KO sham and *n* = 7 KO TAC images were examined over slides from three mice of each group. **h**, Immunoblotting and quantification for Ctgf in heart extracts (*n* = 6 mice examined over two independent experiments). Gapdh was used as loading control. **i**, Relative *Ctgf/Hprt1* and *Tgfb2/Hprt1* expression in TAC and sham heart 2–8 weeks after surgery is shown as fold change against WT sham group (*n* = 6 mice examined over two independent experiments per group). In all panels, error bars represent s.e.m. One-way ANOVA with Bonferroni correction for multiple comparisons was used to calculate *P* values. Source data

**Extended Data Fig. 3:**
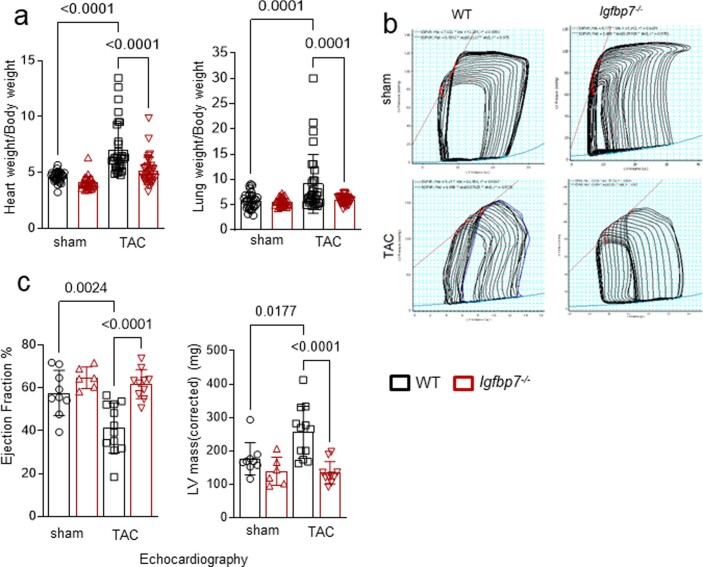
*Igfbp7* deficiency protects mice from pressure overload induced cardiac dysfunction. **(a)** Heart weight/body weight (HW/BW) and lung weight/body weight (LW/BW) ratio at 8 weeks, n = 30 WT and KO sham, 31 WT TAC and 38 KO TAC mice, one-way ANOVA with Bonferroni multiple comparisons was used to calculate *p* values. **(b)** Representative images of pressure–volume loop recordings of WT and *Igfbp7*^*-/-*^ TAC and sham mice, respectively. (**c**) Echocardiographic assessment of cardiac function at 8 weeks post-surgery, ejection fraction (EF) % and corrected LV mass were shown. n = 15 WT sham, 16 WT TAC, 7 Ko sham and 13 KO TAC mice, one-way ANOVA with Bonferroni multiple comparisons was used to calculate *p* values. In all panels error bars represent s.e.m.

To further investigate the role of IGFBP7 in the progression of HF, mice lacking *Igfbp7* (*Igfbp7*^*−/−*^)^29^ and control wild-type (WT) mice were subjected to either TAC or sham control surgery for 8 weeks. Increased heart weight normalized with tibia length (HW/TL) (Fig. 3a), a key indicator of left ventricular hypertrophy, and increased lung weight (Fig. 3b), a sign of congestive HF, were observed in WT mice but not in *Igfbp7*^*−/−*^ mice after TAC surgery (Extended Data Fig. 3a). Wheat germ agglutinin (WGA)-stained heart sections further demonstrated that the increase in cardiac mass in WT TAC heart was mainly due to cardiac myocyte enlargement (Fig. 3c). We evaluated reactivation of fetal genes, a molecular signature of pathological cardiac remodeling and HF^30^, and observed that *Igfbp7* deficiency attenuated elevation of *Nppb* (brain natriuretic peptide), *Nppa* (atrial natriuretic peptide) and *Myh7* (myosin heavy chain 7), in contrast to elevated expression of these genes in WT TAC heart (Fig. 3d). These results suggest that IGFBP7 is a key regulator of pressure-overload-induced HF.

### *Igfbp7* deficiency protects mice from TAC-induced cardiac dysfunction

One of the main characteristics of age-related HF is increase in left ventricular (LV) stiffness^3^. Doppler echocardiographic analysis of the transmitral flow velocity showed that significantly increased isovolumic relaxation time (IVRT) and mitral E/E′ ratio, indicatives of high LV stiffness^31,32^, were noted in WT TAC mice but not in *Igfbp*^*−/−*^ mice (Fig. 3e). We further assessed diastolic function by performing in vivo hemodynamic analysis of LV pressure–volume (PV) relationship. *Igfbp7* deficiency rescued TAC-induced cardiac diastolic dysfunction, as shown by improved LV end-diastolic pressure (LVEDP) and isovolumic relaxation constant (Tau) (Fig. 3f) as well as corrected end-diastolic pressure–volume relationship (EDPVR) (Extended Data Fig. 3b). In addition, *Igfbp7* deficiency abolished TAC-induced decline of LVEF (EF %) and increased LV mass (Extended Data Fig. 3c). Tissue morphometry, echocardiography and PV parameters of the groups are summarized in Supplementary Table 3. In summary, *Igfbp7* deficiency abolished TAC-induced LV dysfunction, further suggesting that *Igfbp7* deficiency protects mice from pressure-overload-induced HF.

### *Igfbp7* deficiency reduces pressure-overload-induced cardiac fibrosis

Myocardial fibrosis is another key pathophysiological feature of HF^4^. Picrosirius red (PSR) staining^33^ of heart histological specimens showed increased fibrosis in WT TAC heart, whereas *Igfbp7* deficiency attenuated collagen accumulation (Fig. 3g). A central mediator of fibrosis, connective tissue growth factor (Ctgf), was increased at the protein level in WT but not *Igfbp7*^*−/−*^ TAC heart (Fig. 3h). This was further confirmed in qRT–PCR measurement of gene expression changes of *Ctgf* as well as *Tgfβ2*, another key regulator of myocardial fibrosis (Fig. 3i). These results suggest that Igfbp7 acts upstream of CTGF and Tgfβ2 to promote cardiac fibrosis.

### *Igfbp7* deficiency protects the heart from cellular senescence

**Fig. 4:**
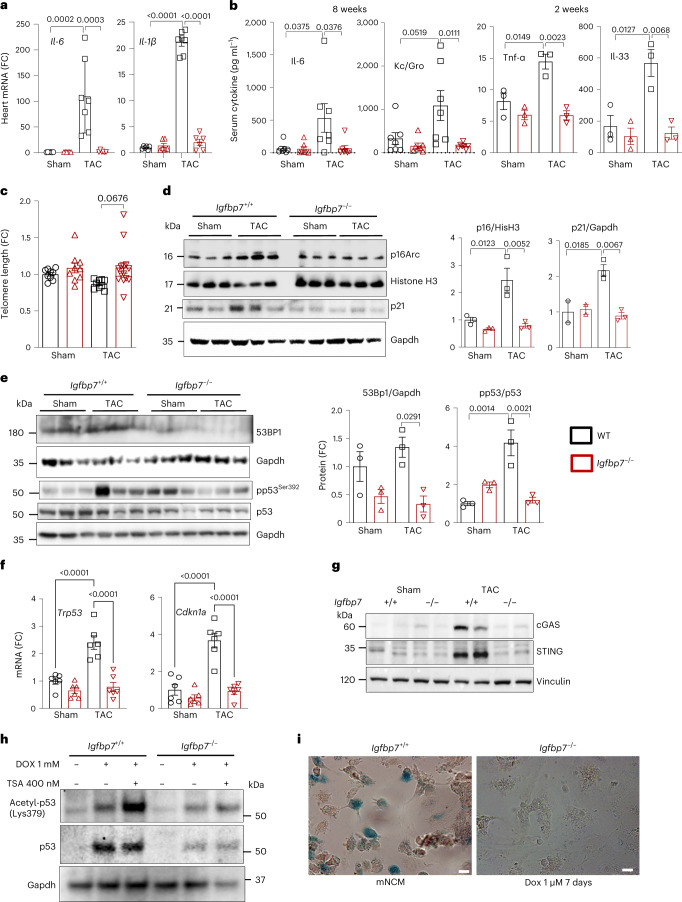
IGFBP7 is required for stress-induced cellular senescence. **a**–**c**, *Igfbp7*^*−/−*^ and WT mice were subjected to TAC or sham surgery and analyzed 2–8 weeks after the operation. **a**, qRT–PCR measurement of relative *Il-6* and *Il-1β* expression shown as fold change against WT sham group in heart samples 8 weeks after surgery (*n* = 6 WT sham, *n* = 6 KO sham, *n* = 6 KO TAC and *n* = 7 WT TAC mice examined over two independent experiments). **b**, Measuring of cytokine levels in blood samples of TAC mice 2 weeks or 8 weeks after the operation by ELISA indicated that *Igfbp7* deficiency blocked TAC-triggered elevation of Il-6, Tnf-α, Kc/Gro and Il-33 in serum (*n* = 7 for 8-week samples, *n* = 3 for 2-week samples). **c**, Relative telomere length measured by quantitative PCR in 8-week post-surgery mouse heart is shown as fold change against WT sham group (*n* = 9 WT sham, *n* = 9 WT TAC, *n* = 9 KO sham and *n* = 14 KO TAC mice examined over three independent experiments). **d**,**e**, Representative immunoblotting and quantification for cellular senescence markers p16ARC and p21 (CIPI/WAF1) (**d**) and 53BP1, phosphor (pp53^ser392^) and total p53 (**e**), in either nuclear fraction (p16ARC) or whole heart extracts. Histone H3 or Gapdh were used as loading control. Protein expression is shown as fold change against WT sham group (*n* = 3 per group). **f**, qRT–PCR measurement of senescence marker *Tp53* and *Cdkn1a* expression in heart samples 2 weeks or 8 weeks after surgery. Gene expression is shown as fold change against WT sham group (*n* = 6 mice examined over two independent experiments). **g**, Representative immunoblotting shows that the innate immune cGAS–STING cytosolic DNA sensing pathway was activated in WT TAC heart. Vinculin was used as loading control. **h**–**i**, *Igfbp7*^*−/−*^ and WT mNCMs were subjected to Dox treatment to induce cellular senescence. **h**, Representative immunoblotting for cellular senescence markers acetylated p53 (acetyl-p53) and total p53 in Dox (1 μM) or Dox (1 μM) + trichostatin A (400 nM) treated mNCMs for 72 hours. Solvent-treated cells were use as control. Gapdh was used as loading control. **i**, Representative microscopy images showing that *Igfbp7* deficiency decreased senescence-associated β-galactosidase-positive cells (blue) in *Igfbp7*^*−/−*^ mNCMs compared to WT mNCMs. Scale bar, 20 μm. In all panels, error bars represent s.e.m. One-way ANOVA with Bonferroni correction for multiple comparisons was used to calculate *P* values. Source data

Increased expression of multiple innate immune inflammatory genes is associated with the development of premature senescence, including key factors of SASP, such as IL-6 and IL-1β^26^. As shown in Fig. 4a, elevated *Il-6* and *Il-1β* gene expression was detected in WT TAC hearts but not in *Igfbp7*^*−/−*^ TAC hearts. Significant increases in the secretion of Il-6, Tnf-α, Kc/Gro (the murine homolog of IL-8) and Il-33 were also seen in plasma of WT TAC mice compared to *Igfbp7*^*−/−*^ TAC mice (Fig. 4b). As telomere shortening is another hallmark of cellular senescence^9^, genomic DNA samples from TAC hearts showed that *Igfbp7* deficiency protected against pressure-overload-induced telomere shortening (Fig. 4c). Subsequently, protein levels of several well-established senescence markers were measured by immunoblotting, including elevated p16Arc and p21Cip1 (Fig. 4d); 53BP1 and phospho-p53/total p53 ratio (Fig. 4e) were detected in WT TAC hearts but not in *Igfbp7*^*−/−*^ TAC hearts. This was further confirmed in mRNA level of *Tp53* and *Cdkn1a* (Fig. 4f).

The cGAS–STING pathway is a component of the innate immune system that functions to detect the presence of cytosolic DNA and, in response, trigger the expression of inflammatory genes that can lead to senescence^34^. cGAS and STING proteins were upregulated in WT TAC hearts, indicating that the increased inflammatory and cellular senescence response could be triggered by pressure-overload-induced cGAS–STING elevation, which was abolished by *Igfbp7* deficiency (Fig. 4g). Next, we further tested whether *Igfbp7* deficiency could protect cardiomyocytes against other stress-induced senescence by exposing mouse neonatal cardiomyocytes (mNCMs) isolated from both WT and *Igfbp7*^*−/−*^ mice to doxorubicin (Dox), a chemotherapy drug that induces cardiotoxicity and premature senescence^35^. Elevated expression of p53 and acetylated p53, hallmarks of cellular senescence^36^, were detected in WT mNCMs but not in *Igfbp7*^*−/−*^ mNCMs (Fig. 4h). Additionally, WT mNCMs exposed to Dox had increased senescence-associated β-galactosidase-positive cells compared to *Igfbp7*^*−/−*^ mNCMs (Fig. 4i).

### IGFBP7 promotes cardiac senescence by simulating IGF-1R/IR-dependent suppression of FOXO3a

**Fig. 5:**
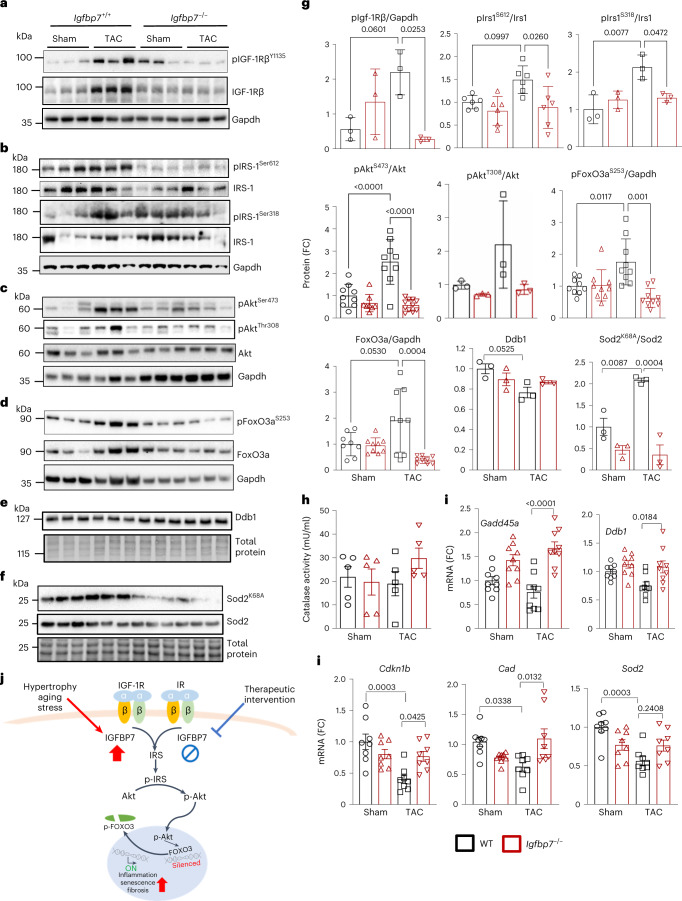
IGFBP7 promotes cardiac senescence by simulating IGF-1R/IR-dependent suppression of FOXO3a. *Igfbp7*^−/−^ and WT mice were subjected to TAC or sham surgery and analyzed 8 weeks after the operation. Representative immunoblotting (**a**–**d**) and quantification (**g**) for activation of IGF-1 receptor (**a**), shown by the ratio of phosphor-IGF-1R to total IGF-1R; IRS-1 (**b**), shown by the ratio of phosphor-IRS-1 to total IRS-1; its downstream activation of Akt (**c**), shown by the ratio of phospho-Akt to total Akt; and inactivation of FoxO3a transcription factor (**d**), shown by the ratio of phospho-FoxO3a and total FoxO3a to Gapdh. Gapdh was used as loading control. Representative immunoblotting (**e**–**f**) and quantification (**g**) show that downregulation of FoxO3a targets Ddb1 in WT mouse hearts (**e**), and acetylation of Sod2 (Sod2^K68A^), as shown by Sod2^K68Ac^ to total Sod2, was upregulated in WT TAC hearts (**f**). Total protein was used as loading control. Protein expression is shown as fold change against WT sham group (*n* = 3 for pIgf-1R^b^, pIrs1^318^, pAkt^308^, Ddb1 and Sod2^k68Ac^ over one experiment; *n* = 6 for pIrs1^S612^ over two independent experiments; and *n* = 9 for pAkt^S473^, pFoxO3a^S253^ and total FoxO3a over three independent experiments). **h**, Increased catalase activity in *Igfbp7*^*−/−*^ TAC hearts as measured by catalase activity assay (*n* = 5 per group). **i**, Relative expression of key FoxO3a-targeted genes *Gadd45a*, *Ddb1*, *Sod2*, *Cad* and *cdkn1b* in heart samples 8 weeks after surgery. Gene expression is shown as fold change against WT sham group (*n* = 9 per group over three independent experiments). In all panels, error bars represent s.e.m. One-way ANOVA with Bonferroni correction for multiple comparisons was used to calculate *P* values. **j**, Diagram shows proposed IGFBP7 action in the stress myocardium. Source data

**Extended Data Fig. 4:**
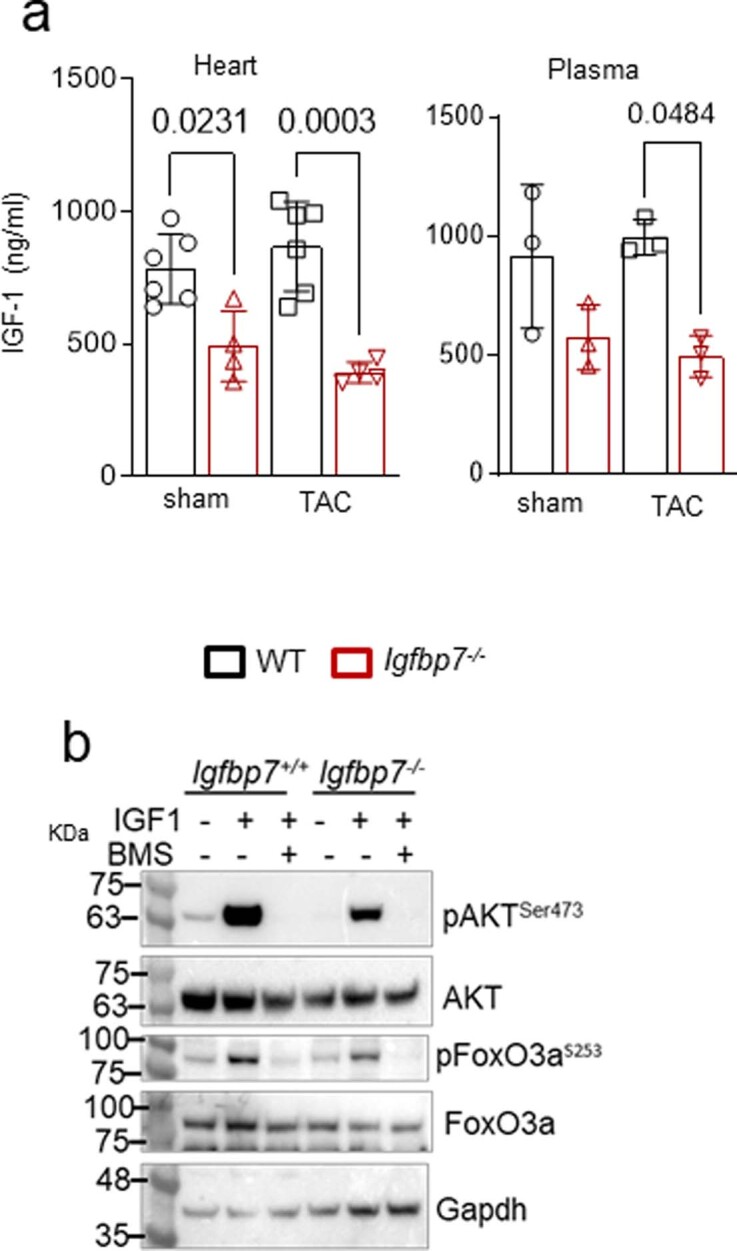
Inhibition of IGFBP7 suppressed IGF1R/AKT induced FOXO3a inactivation. **(a)** Decreased Igf-1 level in *Igfbp7*^*-/-*^ mice hearts and serum as measured by Igf-1 Elisa assay 2 weeks post-surgery. n = 6 for heart lysates, and n = 3 for serum. Error bars represent s.e.m., one-way ANOVA with Bonferroni multiple comparisons was used to calculate *p* values. **(b)** Representative immunoblot shown *Igfbp7* deficiency diminished Igf-1 induced Akt and FoxO3a phosphorylation in mouse neonatal cardiomyocytes (mNCM). Gapdh was used as loading control. Source data

IGFBP7 can significantly influence IGF1/insulin signaling dynamics via extracellular and intracellular pathways^37,38^. To explore if IGFBP7 controls cardiac remodeling by regulation of IGF-1/insulin signaling, we assessed protein levels of Igf-1, a known binding partner of IGFBP7, in heart and plasma samples from WT and *Igfbp7*^*−/−*^ mice. *Igfbp7* deficiency significantly lowered Igf-1 protein levels in both heart tissue and plasma (Extended Data Fig. 4a). Eight weeks after TAC surgery, *Igfbp7* deficiency resulted in downregulation of multiple signal transduction mediators of the IGF-1R/IRS signaling pathway, evidenced by reduced phosphorylation of Igf-1rβ (Fig. 5a) and insulin receptor substrate (IRS-1) (Fig. 5b). *Igfbp7*-deficient heart tissue also exhibited decreased phosphorylation of Akt, a key activator of premature senescence and cardiac hypertrophy^39^ (Fig. 5c). AKT directly phosphorylates FOXO3a, excluding it from the nucleus, thereby inhibiting its transcription activity^40^. We subsequently observed that *Igfbp7* deficiency abolished Akt-mediated FoxO3a suppression, as increased FoxO3a phosphorylation was observed in WT TAC hearts but not in *Igfbp7*^*−/−*^ TAC hearts (Fig. 5d). Two major pathways that control stress resistance and inhibition of cellular senescence, DNA repair and reactive oxygen species (ROS) detoxification, are directly regulated by FOXO3a^41,42^. TAC-induced downregulation of DNA damage-specific binding protein 1 (Ddb1), a target of FOXO3a and a key regulator of double-stranded DNA damage repair, was observed in WT heart, and *Igfbp7* deficiency attenuated the downregulation (Fig. 5e). Increased acetylation of superoxide dismutase 2 (SOD2) was observed in WT heart but not in *Igfbp7*^*−/−*^ heart (Fig. 5f). SOD2, a key detoxification enzyme, lies downstream of FOXO3a, whose acetylation converts its function from an anti-oxidant (dismutase) to a pro-oxidant (peroxidase)^43^. Therefore, *Igfbp7* deficiency maintained Sod2ʼs anti-oxidant activity. Catalase, another key FOXO3a target enzyme for detoxification of ROS, showed increased activity in *Igfbp7*^*−/−*^ TAC heart but was not modulated by TAC in WT heart (Fig. 5h). Furthermore, qRT–PCR analysis revealed downregulation of FOXO3a transcription target genes *Gadd45a*, *Ddb1*, *Cad*, *Sod2* and *Cdkn1b* in WT TAC heart but not in *Igfbp7*^*−/−*^ TAC heart (Fig. 5i). Therefore, as summarized in Fig. 5j, IGFBP7 promotes cardiac senescence by stimulating IGF-1R/IR/IRS/AKT-dependent suppression of FOXO3a, preventing DNA repair and ROS detoxification, suggesting that inhibition of IGFBP7 could be therapeutically beneficial for senescence-related HF.

### *IGFBP7* knockdown blocks hypertrophy and cellular senescence in hCMs

**Fig. 6:**
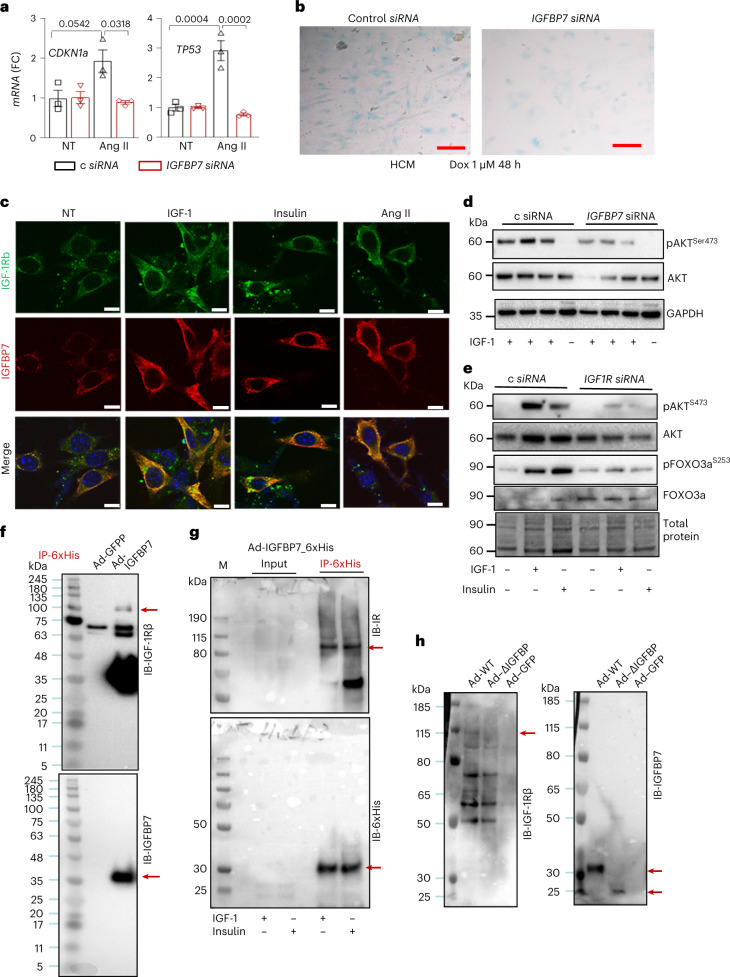
Inhibition of IGFBP7 suppressed IGF1R/AKT-induced FOXO3a inactivation. **a**, Knockdown of *IGFBP7* reduced Ang II-induced *CDKN1a* and *TP53* upregulation in hCMs. Gene expression is shown as fold change against control siRNA-treated hCMs (*n* = 3 per group). Error bars represent s.e.m. One-way ANOVA with Bonferroni correction for multiple comparisons was used to calculate *P* values. **b**, Representative microscopy images showing that *IGFBP7* knockdown decreased senescence-associated β-galactosidase-positive cells in hCMs. Scale bar, 20 μm. **c**, Representative confocal microscopy images showing that IGFBP7 (red) co-localized with IGF-1Rβ (green) in hCMs. Nuclei were stained with DAPI (blue). Scale bars are 10 μm in all images. **d**, Knockdown of *IGFBP7* by siRNA in hCMs blocked IGF-1, and insulin induced AKT phosphorylation; representative immunoblotting of phosphor-AKT and total AKT is shown. Gapdh was used as loading control. **e**, Representative immunoblotting shows that knockdown of *IGF-1R* by siRNA in AC16 human cardiomyocytes has the same effect as *IGFBP7* knockdown in regulating AKT/FOXO3a signaling, which indicates that IGFBP7 regulates AKT/FOXO3a signaling through IGF-1R. **f**–**h**, AC16 hCMs were infected with *ad-IGFBP7-6xHis* and subjected to immunoprecipitation with anti-6xHis Dynabeads; the pulldown was analyzed by western blot with indicated antibodies; and the co-immunoprecipitation pulldown bands are indicated by red arrows. Representative immunoblotting shows that both IGF-1Rβ (**f**) and IR (**g**) were co-immunoprecipitated by anti-6xHis pulldown, whereas deletion of the N-terminal IGFBP motif, as in *Ad-ΔIGFBP-6xHis-*infected cells, reduced IGFBP7 co-immunoprecipitation with IGF-1Rβ (**h**). Immunoblotting with anti-IGFBP7 or anti-6xHis tag was used as control. c, Control. Source data

Next, we further investigated the inhibition of *IGFBP7* expression as a therapeutic target for HF in hCMs by knockdown of IGFBP7 in cardiomyocytes with small interferring RNA (siRNA). When control siRNA-treated hCMs were stimulated with Ang II, a known inducer of myocardial hypertrophy and premature senescence^44^, significantly increased gene expression of key senescence markers *TP53* (p53) and *CDKN1a* (p21) was observed, whereas knockdown of *IGFBP7* by siRNA abolished the increases (Fig. 6a). In addition, significantly increased accumulation of senescence-associated β-galactosidase-positive cells was found in control siRNA-treated hCMs exposed to Dox compared to *IGFBP7* siRNA-treated hCMs (Fig. 6b). Collectively, these findings indicate that IGFBP7, when persistently elevated, accelerates myocyte senescence.

To examine the molecular action of IGFBP7, we tested whether IGFBP7 directly interacts with IGF-1R in hCM cultures simulated with IGF-1, insulin or Ang II. Immunofluorescent staining showed that, upon treatment, IGFBP7 co-localized with IGF-1Rβ (Fig. 6c). When hCMs were exposed to IGF-1 and insulin stimulation, increased phosphorylation of AKT was observed in control siRNA but not in *IGFBP7* siRNA-treated hCMs (Fig. 6d). To further confirm if IGFBP7 regulates AKT/FOXO3a by modulating IGF-1R-mediated signaling, siRNA was used to knock down *IGF-IR* in AC16 hCMs. Consistent with results obtained by *IGFBP7* knockdown, *IGF-IR* knockdown also downregulated AKT/FOXO3 phosphorylation (Fig. 6e). This finding was further confirmed in *Igfbp7*^*−/−*^ and WT mNCM cultures pre-treated with an IGF-1R/insulin receptor (IR) selective inhibitor, BMS-754807, before stimulation with IGF-1 (Extended Data Fig. 4b).

### Requirement of IGFBP motif in modulating IGF-1R/IR signaling

**Extended Data Fig. 5:**
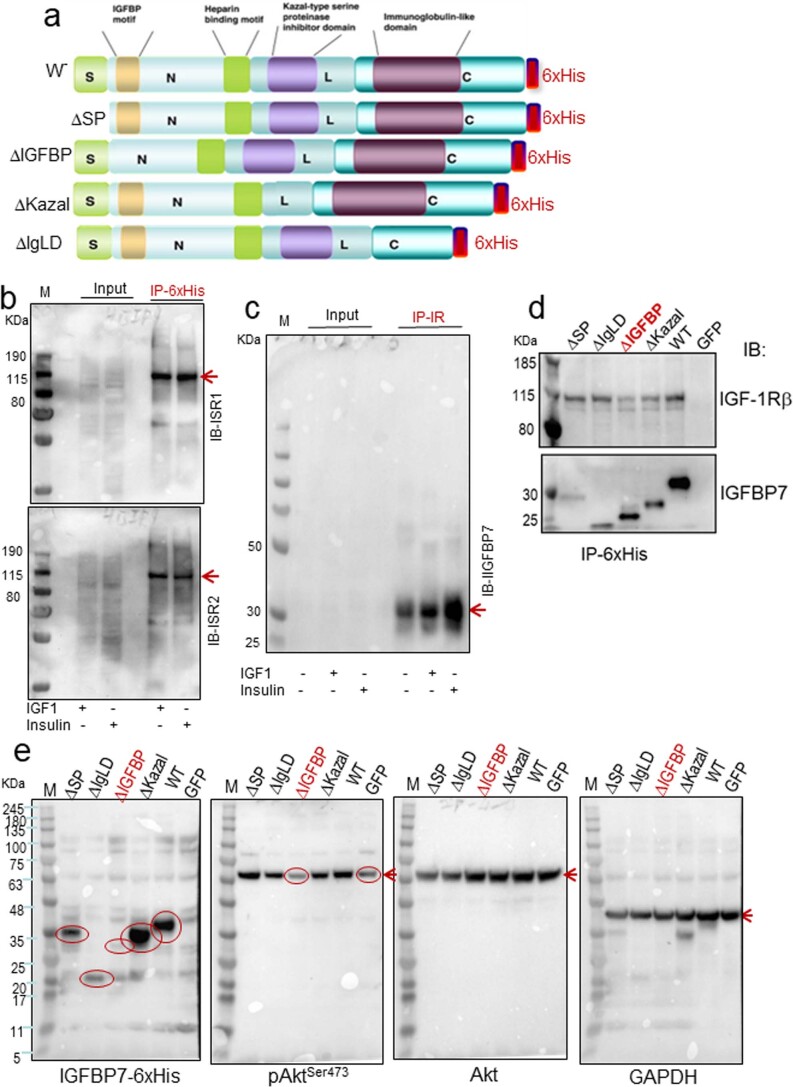
Requirement of IGFBP motif in modulating IGF-1R/IRS signaling. **(a)** Diagrams representing 6xHis-tagged WT (*ad-IGFBP7*) and mutant IGFBP7 (*ad-ΔSP, ad-ΔIGFBP, ad-ΔKazal and ad-ΔIgLD*) adenovirus (Ad) constructs. (**b**,**c)** AC16 cells were infected with *Ad-hIGFBP-6xhis* for 48 hours. **(b)** Representative immunoblot shows IGF-1R and IR binding adaptor protein IRS1 and IRS2 were pulldown by IP with anti-6xHis Dynabeads. **(c)** Representative immunoblot shows IGFBP7 were pull down by reverse IP with anti-IR antibody; The co-IP poll-down bands were indicated by red arrows. (**d**,**e**) AC16 cells were infected with *Ad-hIGFBP7 (WT)*, its mutant forms and control (*ad-GFP*) for 48 hours. **(d)** Representative immunoblot shows deletion of IGFBP motif, reduced IGFBP7 co-IP with IGF-1Rβ. **(e)** Representative immunoblot shown *ad* infected 6xHis-tagged *hIGFBP7* and its mutant forms were readily detected by immunoblot with anti-6His antibody as indicated by red cycle; and deletion of IGFBP motif, diminished AKT phosphorylation. Red cycle indicated down regulation of phosphor-Akt. GAPDH was used as loading control.

Structural characterization of IGFBP7 showed that the protein processed three distinct domains: an N-terminal domain, a C-terminal domain and a linker domain^23^. Based on our finding that IGFBP7 modulated IGF-1R/IR-dependent signaling, we hypothesized that IGFBP7 functions as an adaptor protein, binding to IGF-1R/IR via its N-terminal IGFBP motif. To test this hypothesis, histidine-tagged WT IGFBP7 adenovirus constructs (*Ad-WT IGFBP7-6xHis)* and mutant IGFBP motif deletion adenovirus constructs *(Ad-ΔIGFBP-6xHis*) were generated (Extended Data Fig. 5a). 6xHis-tagged IGFBP7 protein expression was readily detectable in *Ad-IGFBP7-6His-*infected but not in control *Ad*-*GFP*-infected cells. Notably, both IGF-1Rβ (Fig. 6f) and IR (Fig. 6g) were co-immunoprecipitated by anti-6xHis pulldown, whereas deletion of the N-terminal IGFBP motif, as in *Ad-ΔIGFBP-6xHis*-infected cells, reduced IGFBP7 co-immunoprecipitation with IGF-1Rβ (Fig. 6h). In addition, adaptor proteins IRS1 and IRS2, which form a complex with IGF-1R and IR, are also pulled down by IGFBP7-6xHis immunoprecipitation (Extended Data Fig. 5b). Moreover, reverse immunoprecipitation with anti-IR antibody pulled down IGFBP7 (Extended Data Fig. 5c). To further confirm that the IGFBP motif is required for its function in modulating IGF-1R signaling, three additional 6xHis-tagged mutant IGFBP7 adenovirus constructs—*IGFBP7ΔSP-6xHis* (deletion of N-terminal signal peptide), *IGFBPΔKazal-6xHis* (deletion of the linker Kazal-type serine proteinase inhibitor domain) and *IGFBPΔIgLD-6xHis* (deletion of the C-terminal Ig-like domain)—were generated (Extended Data Fig. 5a). Next, AC16 cells were infected with WT and four different *IGFBP7* deletion forms of adenovirus constructs; *Ad-GFP* was used as negative control. As shown in Extended Data Fig. 5d, both WT IGFBP7 and its deletion forms were readily detectable in infected cells with correct corresponding band size. Moreover, only deletion of the IGFBP motif noticeably partially blocked IGFBP7 co-immunoprecipitated with IGF-1Rβ. In addition, IGF-1Rβ-mediated AKT activation was also partially blocked in *AdΔIGFBP*, but no other IGFBP7 mutations infected cells, as shown in Extended Data Fig. 5e. The above results suggest that deletion of the N-terminal IGFBP motif blocks IGFBP7 binding with IGF-1β and IGF-1β-mediated AKT activation, supporting the requirement of IGFBP motif in modulating IGF-1R/IRS signaling.

### Cardiomyocyte-specific knockdown of *Igfbp7* rescued TAC-induced HF in mice

**Fig. 7:**
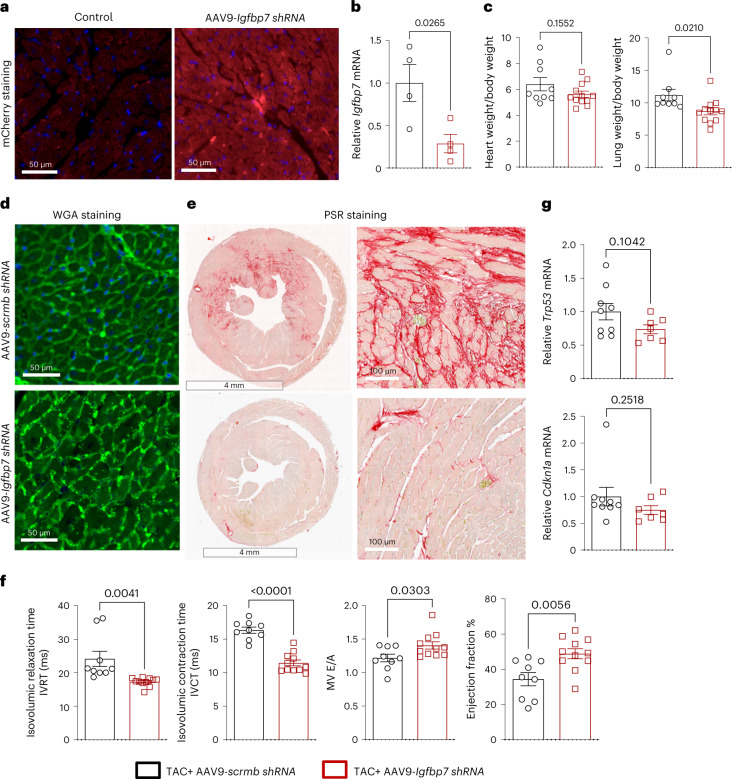
*AAV9*-mediated cardiac-myocyte-specific knockdown of *Igfbp7* rescued TAC-induced HF in mice. *AAV9*-mediated shRNA knockdown of *Igfbp7* in cardiac myocytes by echo-guided LV injection of *AAV9-Igfbp7-shRNA* in post-TAC mice hearts. **a**,**b**, At day 3 after surgery and injection, mCherry staining of heart sections tracked *AAV9-mCherry-U6-mIgfbp7-shRNA* to cardiomyocytes (**a**) (scale bars, 50 μm); meanwhile, qRT–PCR analysis showed that there is a significantly decreased *Igfbp7* mRNA expression in the hearts of *AAV9-mCherry-U6-mIgfbp7-shRNA-*injected mice (*n* = 4) compared to *AAV9-scrmb-shRNA-*injected mouse hearts (*n* = 4) (**b**). **c**–**g**, Four weeks after TAC and injection, *Igfbp7* knockdown attenuated TAC-induced HW/BW and LW/BW increase in *AAV9-mCherry-U6-mIgfbp7-shRNA-*injected mice (*n* = 11) compared to control shRNA*-*injected mice (*n* = 9) (**c**). **d**, Representative WGA staining of transverse heart cross-sections showing myocyte cross-sectional area, which demonstrates that *Igfbp7* knockdown attenuated TAC-induced cardiac myocyte enlargement. Scale bars, 50 μm. **e**, Representative PSR staining of heart sections shows increased collagen deposition in control *AAV9-scrmb-shRNA-*injected TAC hearts, and this is much attenuated by *Igfbp7* knockdown with *AAV9-mCherry-U6-mIgfbp7-shRNA*. Scale bars are 4 mm for top panels and 100 μm for bottom panels. **f**, Echocardiographic analysis shows improved cardiac function in *AAV9-mCherry-U6-mIgfbp7-shRNA-*injected mice (*n* = 11) compared to control shRNA*-*injected mice (*n* = 9). **g**, qRT–PCR revealed that there is a trend of decreased key senescence gene *Trp53* and *Cdkn1a* expression in *AAV9-mCherry-U6-mIgfbp7-shRNA-*injected mice (*n* = 7) compared to control shRNA*-*injected mice (*n* = 9). In all panels, error bars represent s.e.m. Unpaired two-tailed *t-*tests were used to calculate *P* values.

**Extended Data Fig. 6:**
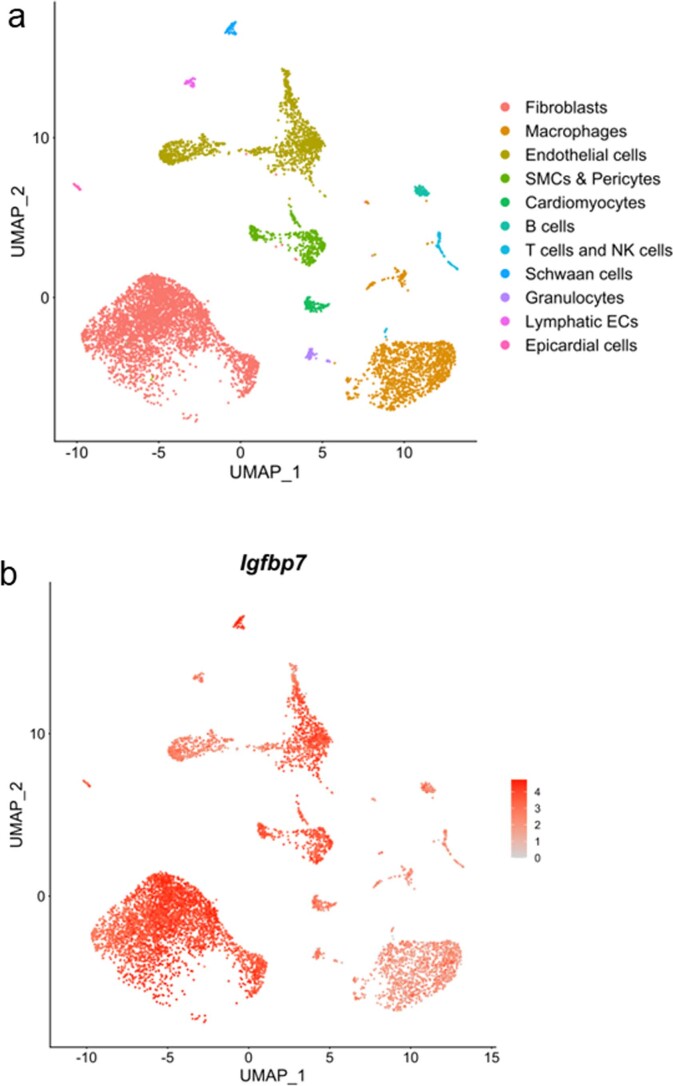
In silicon analysis of publicly available scRNA-seq dataset. In silicon analysis of publicly available scRNA-seq dataset of normal adult mouse heart reveals *Igfbp7 mRNA* is expressed in multiple types of cells of the heart. **(a)** Unbiased clustering of scRNA-seq data from normal adult mouse hearts. **(b)** Featureplot showing expression of *Igfbp7* in multiple cardiac cell populations.

To better understand the expression pattern of *IGFBP7* in the heart at the level of cell specificity, we did an in silico analysis of publicly available single-cell RNA sequencing (scRNA-seq) data of normal adult mouse heart^45^, which shows that *Igfbp7* mRNA is expressed in multiple types of cells of the heart (Extended Data Fig. 6). To address if it is indeed myocardial IGFBP7 that directly regulates pathological cardiac remodeling, AAV9-mediated shRNA knockdown of *Igfbp7* in cardiac myocytes by echocardiography-guided left ventricular intracavitary injection (echo-guided LV injection)^46^ of *AAV9-Igfbp7-shRNA* in post-TAC mice hearts was used. Three days after injection, mCherry staining of heart sections tracked *AAV9-mCherry-U6-mIgfbp7-shRNA* to cardiomyocytes (Fig. 7a); meanwhile, qRT–PCR analysis showed that there is a significantly decreased *Igfbp7* mRNA expression in the hearts of *AAV9-mCherry-U6-mIgfbp7-shRNA*-injected mice compared to *AAV9-scrmb-shRNA-*injected mouse hearts (Fig. 7b). Four weeks after TAC and injection, *Igfbp7* knockdown attenuated TAC-induced heart weight/body weight (HW/BW) and lung weight/body weight (LW/BW) increase in *AAV9-mCherry-U6-mIgfbp7-shRNA*-injected mice (Fig. 7c). WGA staining of heart sections showed that this is due to attenuation of TAC-induced enlargement of myocyte cross-sectional area (Fig. 7d). PSR staining of heart sections showed increased collagen deposition in control *AAV9-scrmb-shRNA-*injected TAC hearts, and this is much attenuated by *Igfbp7* knockdown with *AAV9-mCherry-U6-mIgfbp7-shRNA* (Fig. 7e). Meanwhile, echocardiographic analysis showed improved cardiac function in *AAV9-mCherry-U6-mIgfbp7-shRNA*-injected mice (Fig. 7f). qRT–PCR revealed that there is a trend of decreased key senescence gene *Trp53* and *Cdkn1a* expression in *AAV9-mCherry-U6-mIgfbp7-shRNA*-injected mice (Fig. 7g). Parameters of tissue morphometry and echocardiographic measurement results of the groups are summarized in Supplementary Table 4.

### Antibody-mediated IGFBP7 inhibition in vivo rescued TAC-induced HF

**Fig. 8:**
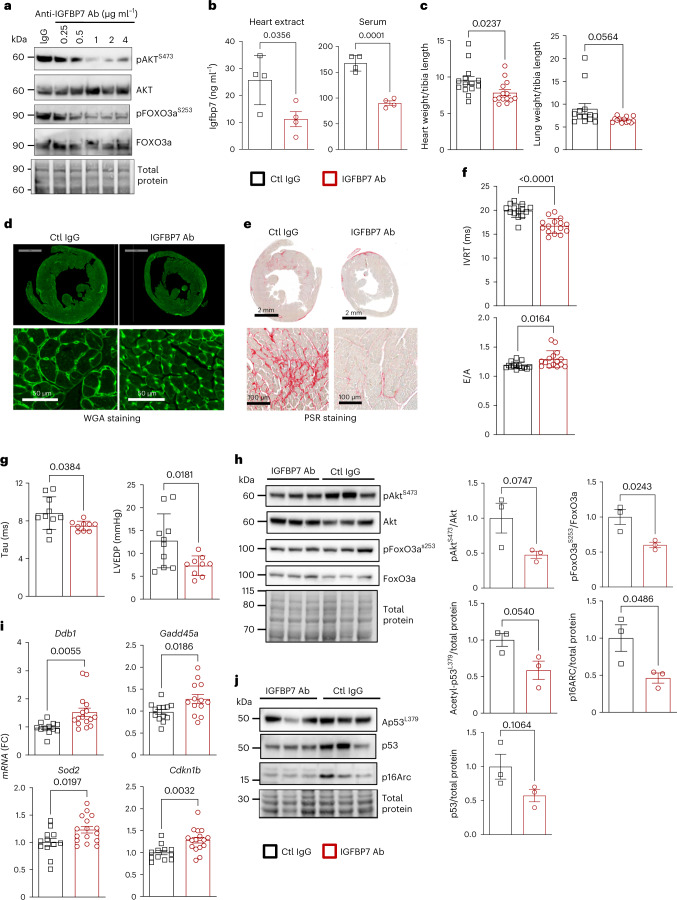
Antibody-mediated IGFBP7 depletion in vivo in mice rescued TAC-induced HF. **a**, Representative immunoblotting showing that blocking IGFBP7 by neutralizing anti-IGFBP7 antibody in human AC16 cardiomyocytes attenuates IGF-1-induced AKT activation and the phosphorylation of FOXO3a. Total protein was used as loading control. **b**–**j**, C57BL/6 mice were subjected to TAC operation and immediately received subcutaneous injection of either 20 μg of rabbit monoclonal IGFBP7 antibody or 20 μg of anti-rabbit IgG isotopy control, followed by analysis at a variety of timepoints after the operation. **b**, ELISA results show that anti-IGFBP7 antibody injection diminished TAC-induced Igfbp7 protein induction in both heart and serum 5 days after treatment (*n* = 4 per group). **c**, HW/TL and LW/TL ratio at 4 weeks (*n* = 14 for each group). **d**, Representative WGA staining of 4-week post-treatment transverse heart cross-sections showing myocyte cross-sectional area. **e**, Representative PSR staining of 4-week post-treatment transverse heart cross-sections shows increased collagen deposition in control IgG-injected TAC heart, and this is much attenuated by Igfbp7 depletion with antibody. In both **d** and **e**, scale bars are 2 mm for top panels and 50 μm for bottom panels. **f**, Transmittal Doppler flow velocity assessment of cardiac function at 4 weeks after TAC and antibody injection; IVRT and mitral E/A ratio are shown (*n* = 14–15 per group). **g**, Measurement of cardiac function by PV conductance catheterization at 4 weeks after surgery further indicated that antibody treatment preserved LV function; isovolumic relaxation constant (Tau) (Weiss model) and LVEDP are shown (*n* = 9–10 per group). **h**, Representative immunoblotting and quantification for activation of Akt, shown by the ratio of phospho-Akt to total Akt, and inactivation of FoxO3a, shown by the ratio of phospho-FoxO3 and total FoxO3. Total protein was used as loading control. Protein expression was shown as fold change against control IgG-treated group (*n* = 3 per group). **i**, Relative expression of key FoxO3a target genes *Gadd45a*, *Ddb1*, *Sod2* and *cdkn1b* in heart samples 4 weeks after TAC and antibody injection. Gene expression is shown as fold change against control IgG-treated group (*n* = 12–15 per group). **j**, Representative immunoblotting and quantification showing that cellular senescence markers acetylated p53 (Ap53^L379^) and total p53 and p16ARC, in whole heart extracts; total protein was used as loading control. Protein expression is shown as fold change against control IgG group (*n* = 3 per group). In all panels, error bars represent s.e.m. Unpaired two-tailed *t-*tests were used to calculate *P* values. Ab, antibody; Ctl, Control. Source data

**Extended Data Fig. 7:**
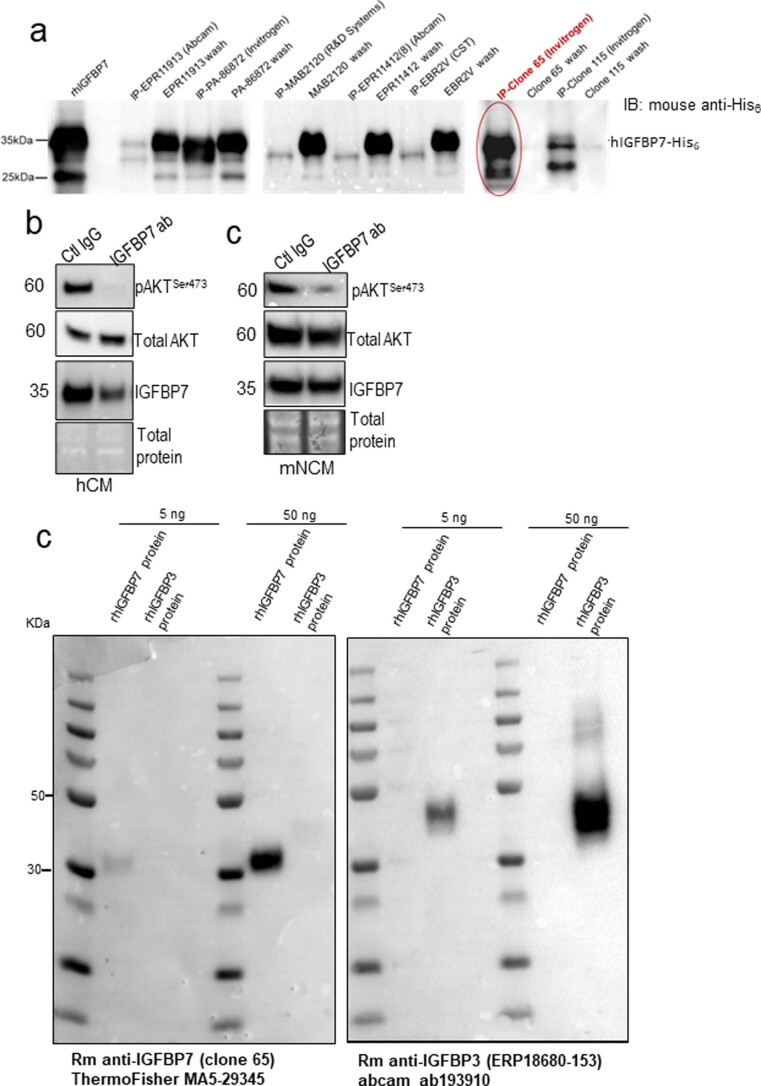
Selection and testing the specificity of IGFBP7 antibody for in vivo neutralization of IGFBP7. **(a)** Representative immunoblot shown seven IGFBP7 antibodies were tested for its binding efficiency to native recombinant human IGFBP7 protein (hIGFBP7) by IP in cell free system, and IGFBP7 recombinant rabbit monoclonal antibody (clone 65, Invitrogen), as indicated by red cycle, has been selected. The IP were pull down with dynabeads protein G immunoprecipitation kit, and blot with anti-6xHis antibody. The first wash through were used as Input. **(b–c)** Representative immunoblot shown blocking IGFBP7 by neutralizing anti-IGFBP7 antibody attenuate IGF-1 induced AKT activation in both **(b)** primary human cardiac myocytes (hCM) and **(c)** mouse neonatal cardiac myocyte (mNCM). Total proteins labeled by no stain protein labeling reagent was used as loading control. **(d)** Representative immunoblotting shows Rm anti- IGFBP7 antibody (clone 68) is only binding to recombinant human IGFBP7 protein (rhIGFBP7) but not recombinant human IGFBP3 protein (rhIGFBP3); and the Rm anti-IGFBP3 antibody (ERP18680-153) is only binding to rhIGFBP3, but not hrIGFBP7. Source data

**Extended Data Fig. 8:**
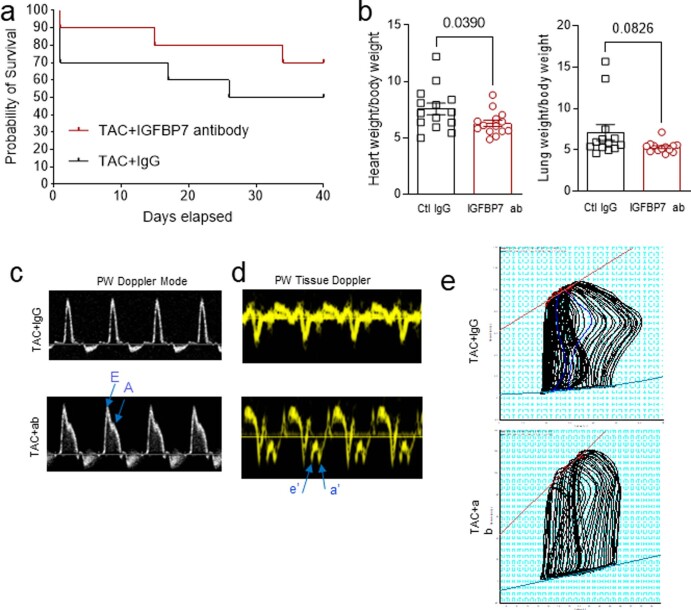
Antibody-mediated IGFBP7 inhibition in vivo rescued TAC-induced HF. **(a)** Mortality rate of IGFBP7 antibody and control IgG treated TAC mice is shown by Kaplan–Meier plots as the survival proportion of each cohort of mice. Survival incidence in mice over the indicated follow-up period was shown as the ratio of the number of mice survived/total mice analyzed for each group. This represents 70% (7/10) for IGFBP7 antibody+TAC mice, 50% (5/10) for control IgG+TAC mice on day 40. **(b)** Heart weight/body weight and lung weight/body weight ratio at 4 weeks, n = 14 for each group, unpaired two-tailed *t-tests* was used to calculate *p* values. (**c**–**e**) Representative images of **(c)** Transmittal doppler flow velocity, **(d)** PW tissue doppler and **(e)** pressure–volume loop recordings of TAC + antibody and TAC + IgG mice, respectively.

To explore inhibition of IGFBP7 in a therapeutically relevant manner, we made use of antibody-targeted inhibition of *Igfbp7* in the TAC mouse model. Initially, we employed a cell-free system to test multiple anti-IGFBP7 antibodies for optimal binding to native recombinant human IGFBP7 protein (hIGFBP). A recombinant rabbit monoclonal anti-IGFBP7 antibody (clone 65) that bound to hIGFBP7 with high affinity (Extended Data Fig. 7a) was selected, and initial testing in cardiac myocytes in vitro showed that pre-treatment with anti-IGFBP7 antibody, before stimulation with IGF-1, effectively attenuated IGFBP7-induced AKT phosphorylation as well as AKT-mediated phosphorylation of FOXO3a (Fig. 8a and Extended Data Fig. 7b,c). In the IGFBP superfamily, IGFBP3 and IGFBP7 have similar structure and functions. To validate the specificity of the anti-IGFBP7 antibody (clone 65), both recombinant human IGFBP7 protein (rhIGFBP7) and human IGFBP3 protein (rhIGFBP3) was used for immunoblotting, and the result indicates IGFBP7 antibody clone 65 binding only to rhIGFBP7 but not to rhIGFBP3 (Extended Data Fig. 7d). Next, we tested whether subcutaneous injection of anti-IGFBP7 antibody clone 65, immediately after TAC surgery in mice, could reduce TAC-induced damage to the heart. ELISA results showed anti-IGFBP7 antibody injection diminished TAC-induced Igfbp7 protein induction in both heart and serum 5 days after treatment (Fig. 8b). Significantly, blockade of Igfbp7 by antibody improved survival (Extended Data Fig. 8a), lowered cardiac mass and lung weight as measured by HW/TL and LW/TL ratio (Fig. 8c and Extended Data Fig. 8b) and reduced ventricular myocyte hypertrophic growth as evaluated with WGA staining (Fig. 8d), collectively indicating improved heart functions. Furthermore, IGFBP7 antibody treatment attenuated fibrotic collagen accumulation as evidenced by PSR staining (Fig. 8e). Pulse-wave Doppler echocardiography focused on the mitral valve showed improved IVRT and mitral E/A ratio in anti-IGFBP7 antibody compared to control IgG-treated mice 4 weeks after surgery (Fig. 8f and Extended Data Fig. 8c); meanwhile, tissue Doppler echocardiography of the mitral annulus showed improved early (E’) and atrial (A’) peak velocities (Extended Data Fig. 8d). In addition, Tau, LVEDP (Fig. 8g) and EDPVR (Extended Data Fig. 8e) approached control levels in anti-IGFBP7 antibody-treated mice as measured by hemodynamics. Parameters of tissue morphometry, echocardiography and PV measurements results of the groups are summarized in Supplementary Table 5. As seen in Igfbp7-deficient mice, Igfbp7 neutralization reduced Akt and FoxO3a phosphorylation and stimulated FoxO3a activation (Fig. 8h), with subsequent transcriptional upregulation of FoxO3a-induced anti-senescence genes, including *Ddb1*, *Gadd45a*, *Sod2* and *Cdkn1b* (Fig. 8i). Anti-IGFBP7 antibody-mediated cardio protection was also shown by reductions in key cellular senescence factors, p16 and p53, at the protein level in the heart (Fig. 8j). In summary, our data demonstrate that targeted IGFBP7 inhibition through therapeutic delivery of neutralizing IGFBP7 antibody rescued TAC-induced HF in mice, which supports it as a potential therapeutic intervention to limit stress-mediated senescence and pro-fibrotic cardiac injury.

## Discussion

IGFBP7 was originally identified by our group as a candidate HF biomarker via proteomic profiling using a murine model of pressure overload HF^11^. Subsequently, circulating IGFBP7ʼs role in HF prognosis and association with diastolic dysfunction have been extensively verified by multiple clinical studies^13–22^. The results we present here advance understanding of HF in that the key concept that emerged is that IGFBP7 has a previously unrecognized role as an upstream regulator of FOXO3a-dependent DNA repair and ROS detoxification signal in the stressed heart. Before this study, little was known about the role of IGFBP7 in the heart or whether it contributed to disease development. Through its interaction with IGF-1, IGFBP7 was found to play an important role during cardiac development, where IGFBP7 is required for embryonic stem cells to commit to a cardiac lineage^47^. In a zebrafish model of IGFBP7 ortholog knockout, Igfbp7 was found to regulate vascular endothelial growth factor A (VEGF-A)-dependent angiogenesis and cardiac growth^48^. The expression of IGFBP7 is not limited to cardiac tissue and has been generally described as a pan-endothelial biomarker under various pathophysiological conditions. Its expression is highest in activated endothelium, where it is stored and released from Weibel–Palade bodies^49^. Its higher level in various tumors has been shown to block VEGF-induced angiogenesis^50^. The structure and function of IGFBP7 share key similarities with members of the CCN family of matricellular proteins^51^, and IGFBP7 has also been shown to be cooperative or complementary with TGF‐β, a contributor to connective tissue formation^52^. CCN members play important roles in cardiac matrix modulation, including remodeling, cellular senescence and fibrosis^53^. The importance of IGFBP7 in contribution to senescence and vascular homeostasis is further highlighted in three other disease states, such as dementia and Alzheimerʼs disease^54^, various cancers^55^ and diabetes and kidney disease^56^. Recently, through integrative analyses of scRNA-seq and spatial transcriptomics, in a study focused on cardiac fibroblasts in regulating the development of HF, it was found that IGFBP7 is secreted from failing cardiomyocytes^57^, which validated our finding that IGFBP7 is robustly upregulated in cardiomyocytes upon stress. Most recently, by evaluating the prognostic value of IGFBP7 in a large cohort of new-onset or worsening HF (BIOSTAT_CHF cohort), it was found that IGFBP7 pathways are involved in different stages of immune system regulation, linking HF to senescence^58^, which serves as a direct validation of our finding in a larger HF cohort.

Here we show that expression of IGFBP7 in cardiomyocytes is robustly increased in patients with HF and in murine pressure overload HF and mediates elevated plasma IGFBP7 and other SASP proteins as well as elevated senescence markers *CDKN2A* (*p16*), *CDKN1A* (*p21*) and *TP53* (*p53*) in peripheral blood. Taken together, the data of this study strongly implicate elevated IGFBP7 as a key indicator of HF and a driver of chronic inflammation and accelerated cellular senescence. Subsequent in vivo studies in the murine model of pressure overload HF demonstrated that *Igfbp7* deficiency attenuated cardiac dysfunction and adverse ventricular remodeling, with reductions in cardiac inflammatory injury and tissue fibrosis. Notably, *Igfbp7* deficiency diminished cellular senescence in the heart, as supported by reduced innate immune cGAS–STING activation, decreased pro-inflammatory cytokine induction and reserved telomere length. The IGF-1/IR intracellular signaling pathway (ILS) has been recognized as one of the dominant pathways in the regulation of cellular senescence^40^. Multiple studies have shown that inhibition of components of ILS signaling extended lifespan in animals^10^. Our data reveal that IGFBP7 promotes adverse cardiac remodeling and senescence by stimulating ILS-dependent suppression of the anti-senescence factor FOXO3a, preventing DNA repair and ROS detoxification. Functional domain analysis by employing truncation mutant expression further revealed that the IGFBP motif is required for IGFBP7ʼs function in modulating ILS signaling. This detrimental effect of IGFBP7 can be reduced by antibody neutralization of IGFBP7 or siRNA-targeted IGFBP7 ablation. Moreover, AAV9-shRNA-mediated cardiac-myocyte-specific knockdown of *Igfbp*7 rescued TAC-induced HF in mice, which indicated that it is myocardial Igfbp7 that directly regulates pathological cardiac remodeling.

Therapeutic strategies that safely interfere with the detrimental effects of cellular senescence, such as selective elimination of senescent cells or disruption of the SASP secretome, known as senotherapy, are gaining considerable attention^59^. Previous work has implicated sacubitril–valsartan, an angiotensin receptor–neprilysin inhibitor, as a means to modestly reduce IGFBP7 concentrations in chronic HFpEF^18^. However, a more direct, anti-IGFBP7 therapy is a promising concept that may minimize the off-target effects of senotherapeutics and is an important consideration for research. An mAb-mediated approach via blockade of specific SASP factors has the benefit of a well-defined target and, therefore, potentially a low risk of off-target effects. Given the unfavorable role of IGFBP7 in the development and progression of HF, and its nature as a member of the SASP, blockade of IGFBP7 could be beneficial. Here we demonstrated antibody-mediated IGFBP7 neutralization in vivo through therapeutic delivery of IGFBP7 mAbs in a TAC mouse model; reversed IGFBP7 suppression of FOXO3a; restored anti-senescence pathways; and attenuated pressure-overload-induced HF in mice. These data suggest the potential benefit of developing therapeutic strategies that safely interfere with IGFBP7 for treatment of chronological age-related HF.

## Methods

### Human sample collection and biomarker assay

The collection and use of human samples in this study were approved by the Ottawa Health Science Network Research Ethics Board (REB), and informed consent was obtained from patients/family members (Ottawa Health Science Research Board REB no.: 20140869-01H). Blood samples were drawn into 6-ml K2 EDTA tubes (BD, 367863), and plasma was separated by centrifuge at 1,500*g* for 15 minutes at 4 °C. Blood samples for RNA isolation and analysis were collected using Tempus Blood RNA Tubes (Thermo Fisher Scientific, 4342792). All samples were stored at −80 °C until assay. Plasma IGFBP7 was measured using a pre-commercial Elecsys assay (Roche Diagnostics) on a Cobas e411 platform (Roche Diagnostics). The IGFBP7 assay has coefficient of variation (CV) of intra-assay precision <3%, inter-assay precision <6% and lower detection limit at 0.01 ng ml^−1^. Meanwhile, NT-proBNP was also measured in the same samples using the Elecsys proBNP II assay (Roche Diagnostics, 04842464 119) on a Cobas e411 platform as a reference standard. The proBNP II assay has a CV of 2.9–6.1% and a measurement range of 5–35,000 pg ml^−1^ or up to 70,000 pg ml^−1^ for two-fold diluted samples, the same as we reported previously^60^. Targeted aptamer-based SOMAscan assay (1.3K version, SomaLogic) was used to map SASP changes in plasma samples, and analysis was performed with SomaSuite (version 1.0.20120704, SomaLogic). Next, 50 μl of plasma samples was serially diluted to 40%, 1% and 0.005% to achieve a broad dynamic range. The diluted samples were introduced to the bead-immobilized SOMAmers and equilibrated for 3.5 hours at 28 °C and 800 r.p.m. on a ThermoMixer (Eppendorf). SOMAmer-bound proteins were then tagged with biotin, and SOMAmer–protein complexes were released from the beads by a photocleavage process (12 minutes under 360-nm ultraviolet light). All three dilutions per sample were recombined, and the biotinylated SOMAmer–protein complexes were captured on streptavidin-coated beads. After further washing steps, bound SOMAmers were released from the protein targets and loaded onto microarray chips and hybridized for 19 hours at 55 °C and 20 r.p.m. The nucleotides were quantitated using an Agilent SureScan G4900 Microarray Scanner (Agilent Technologies). Quantitative analysis of individual aptamer-coded protein targets was performed using SomaSuite, the same as we reported previously^61^. IGFBP7 protein expression in human and mouse samples was detected by immunofluorescent staining and immunoblotting analyses using standard procedures. Explanted heart samples from a transplant recipient who suffered HF were used for this study. Samples were fixed with neutral-buffered 10% formalin solution (Sigma-Aldrich, HT501128) overnight, embedded in paraffin and sectioned to a thickness of 5 μm.

### Mice strains and creation of pressure overload mouse model

*Igfbp7*-null mice in CD1 background were obtained from Arun Seth’s group at Sunnybrook Research Institute and were generated as reported previously^29^. Mice were maintained at the Animal Care and Veterinary Service Facility at the University of Ottawa. All animal experimental protocols were approved by the Animal Care and Use Committee at the University of Ottawa and performed in accordance with institutional guidelines.

As previously described, 10–12-week-old *Igfbp7*^*−/−*^ and control WT mice with a body weight of approximately 25 g were subjected to pressure overload by TAC^28^. In brief, mice were anesthetized with 1–2% (per liter of O_2_) isoflurane during the operation. The chest was opened, and a horizontal skin incision was made at the level of the 2–3 intercostal spaces. The start of the descending aorta was identified right after the subclavian branch. A 7-0 silk suture was placed around the beginning of the descending aorta and tied around a 26-gauge blunt needle, which was subsequently removed. At the end of the procedure, the chest and skin were closed. The mice were kept on a heating pad until responsive to stimuli. Sham-operated animals underwent the identical procedure, except that the aortic constriction was not placed. The mice were monitored for up to 8 weeks after surgery, and their heart functions were determined by serial echocardiography before surgery and at 2 weeks, 4 weeks and 8 weeks after surgery. Mice were randomized to sacrifice on weeks 2 and 8 after banding, for evaluation of morphology, function and detailed molecular expression analysis, as well as blood sampling for production of Igfbp7 in serum. For histological analysis, hearts and lungs were arrested with 1 mol L^−1^ of KCl and fixed with neutral-buffered 10% formalin solution (Sigma-Aldrich, HT501128). For mRNA and protein analyses, hearts were snap-frozen in liquid nitrogen and stored at −80 °C until analysis.

### Antibody administration

As described above, 12–14-week-old WT C57BL/6 mice (Charles River Laboratories) with a body weight of approximately 25 g were subjected to TAC surgery. Immediately after surgery, mice received a subcutaneous injection of either 20 μg of recombinant rabbit monoclonal anti-IGFBP7 antibody (clone 65) (Invitrogen, MA5-29345) or 20 μg of rabbit (DA1E) mAb IgG XP isotope control (Cell Signaling Technology, 3900). The mice were monitored for up to 5 weeks after injection, following the same procedure as above. To track the patten of Igfbp7 inhibition in serum and the heart after injection, in a separated group, mice were randomly sacrificed at multiple timepoints; heart and blood were sampled; and Igfbp7 protein levels were measured by ELISA assay (Abcam, ab245712), as described in the ‘ELISA’ section below.

### Echo-guided LV injection of *AAV9-Igfbp7-shRNA* in post-TAC mice heart

Twenty-two-week-old WT CD-1 mice (Charles River Laboratories) were subjected to TAC surgery as described above (owing to the greater body weight of these mice, we adjusted the blunt needle use for banding from 26-gauge to 25-gauge), followed by echo-guided LV injection^45^ of *AAV9-mCherry-U6-mIgfbp7-shRNA* (3.5 × 10^11^ genome copies (GC) per mouse; Vector Biolabs, 7000). The same amount of *AAV9-GFP-U6-scrmb-shRNA* (Vector Biolabs, 77777) was injected to the control group. The mice were monitored for up to 4 weeks after injection, following the same procedure as above.

### Measurement of cardiac function in mice

Cardiac function was determined by transthoracic echocardiogram, tissue Doppler imaging (TDI) and pulse-wave Doppler imaging using Vevo 2100 or Vevo 3100 imaging system (FUJIFILM VisualSonics,). A 40-MHz transducer was used for the imaging. Qualitative and quantitative measurements were made using Vevo LAB analytic software (FUJIFILM VisualSonics). After removing hair from the left chest, mice were anesthetized with 1–2% (per liter of O_2_) isoflurane for the duration of the recordings. Diastolic function was assessed using pulsed-wave Doppler imaging of the transmitral filling pattern with the early transmitral filling wave (E-wave) and the late filling wave owing to atrial contraction (A-wave). IVRT was measured as the time from closure of the aortic valve to the initiation of the E-wave. Diastolic function was also measured by TDI. TDI was made at the inferolateral region in the four chambers view at the base of the LV with the assessment of early diastolic (E′) and late diastolic (A′) myocardial velocities. Systolic function (ejection fraction and stroke volume) was estimated by two-dimensional area measurements of the endocardial wall at systole and diastole using long-axial electrocardiogram-gated kilohertz visualization (EKV) image analysis; dimensional change and myocardial wall thickness were estimated by measurement of short-axial images. M-mode images were obtained for measurements of LV wall thickness, LV end-diastolic diameter and LV end-systolic diameter. LVEF was calculated as a measure of systolic function.

In vivo hemodynamic measurements of cardiac functions using PV conductance catheterization were performed before sacrifice under 1–1.5% isoflurane anesthesia in close-chested animals using a 1.4-French Millar catheter, and measurements were acquired with the Millar Pressure–Volume System (MPVS) (AD Instruments). The PV loop data were then analyzed using LabChart 8.0 (AD Instruments).

### Histology and immunofluorescent staining

For morphometry, 10% neutral-buffered formalin (Thermo Fisher Scientific, 28-600-67) fixed hearts and lungs were embedded in paraffin and sectioned to a thickness of 5 μm. Alexa Fluor 488-conjugated WGA-stained (1 μg ml^−1^; Thermo Fisher Scientific, W11261) sections were used for measurement of heart morphology, and cardiomyocyte cross-sectional areas were measured using FV10-ASW4.2 Viewer software (Olympus). Nuclei were stained with Hoechst 33342 (1 μg ml^−1^; Thermo Fisher Scientific, WH21492). For detection of fibrotic areas, sections were stained with PSR (Abcam, ab150681) to visualize collagen fibers. For immunofluorescent staining, samples were fixed with 4% paraformaldehyde in PBS (Thermo Fisher Scientific, AAJ19943K2), and paraffin sections were performed with minimal antigen retrieval in 10 mM sodium citrate buffer (MilliporeSigma, 6132-04-3), followed by a cell permeabilization step with 0.1% Triton X-100 (Thermo Fisher Scientific, BP151-100) in PBS. After block with 10% FBS (Thermo Fisher Scientific, 12483020) in PBS for 30 minutes at room temperature, the following antibodies were used for staining: anti-IGFBP7 (Abcam, ab74169, 1:200); FM 1-43FX membrane probe (5 μg ml^−1^) (Thermo Fisher Scientific, F35355); anti-IGF1 receptor (Abcam, ab131476, 1:100); mouse monoclonal anti-vimentin (V1-10) (Abcam, ab20346, 1 μg ml^−1^); and Alexa Fluor 568-conjugated isolectin GS-IB4 (Thermo Fisher Scientific, I21412). After overnight incubation with primary antibody, the sections were incubated with a matching Alexa Fluor dye-conjugated secondary antibody (Thermo Fisher Scientific, A21443, A32740, A48283, A48289, A11031 and A11029, all at 1:1,000) (Cell Signaling Technology, 4412 and 4408 at 1:1,000) at room temperature for 1 hour, followed by nuclear stain with Hoechst 33342 (1 μg ml^−1^) (Thermo Fisher Scientific, WH21492) at room temperature for 10 minutes and were mounted with Dako fluorescence mounting medium (Agilent Dako, S302380-2) and subjected to confocal examination on a Leica Aperio VERSA 8 slide scanner, an Olympus FluoView 1000 laser scanning confocal microscope or a Zeiss Elyra S.1 LSM 880 Airyscan confocal microscope. The total fluorescence intensity from the staining was measured using Aperio ImageScope viewing version 12.4 (Leica Biosystems) or Olympus FV10-ASW4.2 Viewer software.

### Immunoblotting

Whole-cell lysates from tissue and cell samples were prepared on ice with cell/tissue lysis buffer (Cell Signallng Technology, 9803; FroggaBio, 17081) containing a complete cocktail of proteases and phosphatase inhibitors (Thermo Fisher Scientific, A32961; Roche Applied Science, 05892791001). Lysates were cleared by centrifugation at 12,000 r.p.m. for 10 minutes. The supernatants were collected, and protein concentrations were determined using either the Bio-Rad Bradford protein assay (Bio-Rad, 500-0006) or the Qubit Protein Assay Kit (Thermo Fisher Scientific, Q33211). Next, 10–20 μg of protein lysate was separated by Bolt Bis-Tris Plus mini-gel system (Thermo Fisher Scientific) and electrophoretically transferred to PVDF membranes (Bio-Rad, 162-0177). Membranes were incubated overnight at 4 °C with antibodies reactive to the following proteins: IGFBP7 polyclonal antibody (Thermo Fisher Scientific, PA1-86872, 1:1,000); anti-IGFBP7 (EPR11913(B)) (Abcam, ab170932, 1:1,000); Rb mAb to IGFBP3 (Abcam, ab193910, 1:1,000); CTGF (Abcam, ab6992, 1:1,000); anti-53BP1 (Abcam, ab36823, 1:1,000); anti-p21 (EPR18021) (Abcam, ab188224, 1:1,000); anti-p16ARC (EP1551Y) (Abcam, ab51243, 1:1,000); anti-histone H3 (Abcam, ab1791, 1:1,000); phospho-p53 (Ser392) (Cell Signaling Technology, 9281, 1:1,000); acetyl-p53 (Lys379) (Cell Signaling Technology, 2570, 1:1,000); p53 (Developmental Studies Hybridoma Bank (DSHB) product PCRP-TP53-1F7, 0.5 μg ml^−1^; PCRP-TP53-1F7 was deposited to the DSHB by Protein Capture Reagents Program, produced by Johns Hopkins University/CDI Laboratories); phospho-IGF-1 receptor β (Thy1135) (Cell Signaling Technology, 3918, 1:500); IGF-1 receptor β (Cell Signaling Technology, 9750, 1:500); phospho-IRS-1 (Ser612) (Cell Signaling Technology, 3203, 1:500); phospho-IRS-1 (Ser318) (Cell Signaling Technology, 5610, 1:500); IRS-1 (Cell Signaling Technology, 3407, 1:500); anti-IRS1 (Millipore, 06-248, 1:1,000); IRS-2 antibody (Cell Signaling Technology, 4502, 1:1,000); INSR antibody (18-44) (Thermo Fisher Scientific, MA1-10865, 1:1,000); phospho-Akt (Ser473) (D9E) (Cell Signaling Technology, 4060, 1:1,000); phospho-Akt (Thr308) (Cell Signaling Technology, 4056, 1:1,000); Akt (pan) (C67E7) (Cell Signaling Technology, 4691, 1:1,000); phosphor-FOXO3A (S253) (EPR1951(2)) (Abcam, ab154786, 1:1,000); FOXO3A (Abcam, ab109629,1ː1,000); insulin receptor β (C18C4) (Enzo, ADI-905-683, 1:1,000); anti-cGAS (Cell Signaling Technology, 15102, 1:1,000); anti-STING (MilliporeSigma, MABF270, 1 μg ml^−1^); anti-DDB1 (Abcam, ab109027, 1:50,000); anti-SOD2 (acetyl K68) (Abcam, ab137037, 1:1,000); and anti-SOD2 (Abcam, ab68155, 1:1,000). Blots were incubated with HRP-conjugated goat anti-mouse IgG (Bio-Rad, 170-5047, 1:100,000); goat anti-rabbit IgG (Bio-Rad, 170-5046, 1:100,000); and monoclonal mouse anti-goat IgG (Jackson ImmunoResearch, 205-032-176, 1:50,000) and developed using Clarity Western ECL Substrate (Bio-Rad, 170-5061) or SuperSignal West Pico PLUS Chemiluminescent Substrate (Thermo Fisher Scientific, 34580). To detect low-abundant protein, SuperSignal West Femto Maximum Sensitivity Substrate (Thermo Fisher Scientific, 34095) or SuperSignal West Atto Ultimate Sensitivity Chemiluminescent Substrate (Thermo Fisher Scientific, A38544) was used. The intensities of the chemiluminescence signals were detected using the ChemiDoc XRS+ System (Bio-Rad, 1708265) and quantified using Image Lab software (version 6.1, Bio-Rad). To normalize signals to total protein, blot membranes were either pre-stained with No-Stain Protein Labeling Reagent (Thermo Fisher Scientific, A4449) or stripped and re-probed with antibody against GAPDH (Thermo Fisher Scientific, MA5-15738, 1:5,000) or vinculin (MilliporeSigma, V4505, 1:1,000), and the results are shown as fold change against control.

### ELISA

Blood samples were collected via posterior vena cava from mice at the time of sacrifice (2–8 weeks after surgery). Serum samples were obtained by centrifuging blood samples at 1,500*g* for 20 minutes at 4 °C and storing at −80 °C until use. Mouse whole heart lysate was prepared on ice from frozen samples in 1× Cell Lysis Buffer (Cell Signaling Technology, 9803) and cleared by centrifugation at 12,000 r.p.m. for 10 minutes. The supernatants were collected, and protein concentrations were determined using the Bradford protein assay (Bio-Rad, 500-0006). For ELISA detection of Igfbp7, Igf-1, Il-6 and catalase activity in either mouse serum or heart lysate, mouse Igfbp7 ELISA kit (Abcam, ab245712), Catalase Activity Assay Kit (Abcam, ab83464), R&D Systems Mouse/Rat IGF-I (MG100) and Mouse IL-6 (M6000B) Quantikine ELISA Kit were used according to manufacturer protocols with PerkinElmer EnVision multimode plate reader. For multiplex ELISA detection of a selected custom panel of cytokines in serum, Meso Scale Discovery’s U-PLEX platform was used as per manufacturer protocols with a MESO QuickPlex SQ 120 Instrument.

### Isolation and culture of mNCMs

Cell culture of mNCMs was prepared from newborn *Igfbp7*^*−/−*^ and WT mouse hearts within 48 hours of birth, as previously reported^33^. In brief, after trimming, left ventricles were mechanically minced in Ca^2+^-free and Mg^2+^-free HBSS on ice and then subjected to stepwise enzymatic digestion with 0.15% trypsin (Thermo Fisher Scientific, 27250018) in disassociation solution (137 mmol L^−1^ NaCl, 5.36 mmol L^−1^ KCl, 0.81 mmol L^−1^ MgSO_4,_ 5.55 mmol L^−1^ dextrose, 0.44 mmol L^−1^ KH_2_PO_4,_ 0.34 mmol L^−1^ Na_2_HPO_4_7H_2_O and 20.06 mmol L^−1^ HEPES, pH 7.4). Cells released after the first digestion were discarded, whereas cells from subsequent digestions were transferred into Gibco DMEM/F-12 medium (Thermo Fisher Scientific, 11320033), supplemented with 10% FBS (Thermo Fisher Scientific, 12483020), until all cardiac cells were isolated (∼5 times). The resulting mixture was centrifuged for 5 minutes at 800 r.p.m., resuspended in DMEM/F-12 medium and pre-plated for 2 hours to remove non-cardiomyocytes based on the observation that non-muscle cells attach to the substrata more rapidly. The cardiomyocytes were then collected and plated on laminin-coated culture plates at a density of 2 × 10^6^ cells per milliliter in DMEM/F-12 plus 10% FBS and 0.1 μM bromodeoxyuridine (BrdU) to inhibit the growth of non-myocytes. Cells were incubated at 37 °C with 5% CO_2_ in a humidified atmosphere. A confluent monolayer of spontaneously beating cardiomyocytes was formed within 2 days and was ready for downstream gene transfer and treatment. For induction of cellular senescence, *Igfbp7*^*−/−*^ and WT mNCMs were treated with Dox (MilliporeSigma, D1515) (1 μM) or Dox (1 μM) + trichostatin A (MilliporeSigma, T8552) (400 nM) for 72 hours, before harvesting for immunoblotting with cellular senescence marker acetylated p53 (acetyl-p53) and total p53 as described in the ‘Immunoblotting’ section. Solvent-treated cells were used as control (NT).

### qRT–PCR

Total RNA from patients’ whole blood samples stored in Tempus Blood RNA Tubes was isolated using Tempus Spin RNA Isolation Kit (Thermo Fisher Scientific, 4380204). RNA concentrations were quantified using a fluorescence-based Qubit RNA BR Assay Kit (Thermo Fisher Scientific, Q10211) with a Qubit Fluorometer. cDNAs were synthesized from 1 μg of total RNA with the SuperScript IV VILO Master Mix (Thermo Fisher Scientific, 11756050). Target gene-specific PCR primers were obtained from Bio-Rad (PrimerPCR SYBR Green Assay, 10025636; human *TP53* (qHsaCID0013658), *CDKN1A* (qHsaCID0014498) and *CDKN2A* (qHsaCED0056722)). qRT–PCR was carried out using BrightGreen qPCR MasterMix (abm, MasterMix-S). Isolation of total RNA from mouse heart tissues was performed using TRIzol reagent (Thermo Fisher Scientific, 15596026) and from cultured cells using PureLink RNA Mini Kit (Thermo Fisher Scientific, 12183018A). cDNAs were synthesized from 1 μg of total RNA with either iScript Reverse Transcription Supermix for RT–PCR (Bio-Rad, 170-8841) or 5× All-In-One RT MasterMix with AccuRT (abm, G592). Target gene-specific intro-spanning forward and reverse primers were designed by primer3 and BLAST using the NCBI Primer-BLAST tool (https://www.ncbi.nlm.nih.gov/tools/primer-blast/index.cgi?LINK_LOC=BlastHome). qRT–PCR was carried out using BrightGreen qPCR MasterMix (abm, MasterMix-S) or PowerUp SYBR Green Master Mix (Thermo Fisher Scientific, A25918) in Hard-Shell 96-well PCR plates (Bio-Rad, HSR9905K) on a LightCycler 96 System (Roche Life Science, 05815916001). All reactions were run in triplicate; gene expressions were normalized to *HPRT1* (hypoxanthine-guanine phosphoribosyltransferase 1) housekeeping gene; and the results are shown as fold change against control. Primer sequences used for qRT–PCR are listed in Supplementary Table 6.

### Quantitative PCR assay for relative telomere repeat DNA measurement

Genomic DNA was extracted from 8-week post-surgery mouse heart samples using a spin-column based DNeasy Blood & Tissue Kits (Qiagen, 69506) according to the manufacturer’s protocol. DNA concentrations were quantified using fluorescence-based Qubit dsDNA BR Assay Kit (Thermo Fisher Scientific, Q32850) to ensure sufficient quantity and purity. Relative telomere repeat copy number was measured with a real-time PCR-based assay that compares telomere repeat sequence copy number to the single-copy gene 36b4 (encodes acidic ribosomal phosphoprotein) as reported^62^. In brief, after optimizing the reaction conditions by running a six-point standard curve (two-fold dilution from 90 ng to 2.8125 ng) for telomere and a five-point standard curve (two-fold dilution from 60 ng to 3.75 ng) for 36b4 from a pool of control mouse DNAs not related to the study, PCR reactions were performed in triplicate in 20-μl reaction volumes with SsoAdvanced Universal SYBR Green Supermix (Bio-Rad, 1725270) and 10 pmol of telomere-specific primers (forward: CGGTTTGTTTGGGTTTGGGTTTGGGTTTGGGTTTGGGTT; reverse: GGCTTGCCTTACCCTTACCCTTACCCTTACCCTTACCCT) or 36b4 primers (forward: ACTGGTCTAGGACCCGAGAAG; reverse: TCAATGGTGCCTCTGGAGATT) and 10 ng of DNA sample on a LightCycler 96 System (Roche Life Science). The thermal cycling profile for all reactions consisted of 5 minutes at 95 °C polymerase activation and DNA denature step, followed by 35 cycles of 95 °C for 15 seconds, 56 °C for 20 seconds and 72 °C for 30 seconds. After determining C_q_ value using the LightCycler 96 application software (Roche Life Science), data were exported to Microsoft Excel, formatted and analyzed with the comparative cycle threshold (Ct) method (2^−ΔΔCt^) to calculate telomere/single-copy gene (T/S) ratios and, thereby, relative differences in the amount of telomere repeat DNA between each group. Data are shown as fold change against WT sham control. Statistical comparisons were made using one-way ANOVA.

### siRNA knockdown of *IGFBP7* and *IGF1R* in hCMs

Either primary hCMs isolated from the ventricles of adult hearts obtained from PromoCell (c-12810) or the AC16 hCM cell line (AC16) (MilliporeSigma, SCC109) was used for siRNA knockdown, as indicated. hCMs were seeded in a six-well plate containing myocyte growth medium (PromoCell, C-22070), at a density of 10,000 cells per cm^2^, in a humidity-controlled incubator at 37 °C in 5% CO_2_ for about 24 hours until cells reached nearly 70% confluency before starting siRNA treatment. Knockdown of *IGFBP7* in hCMs was achieved using two Silencer Select pre-designed siRNAs specific to human *IGFBP7* (Thermo Fisher Scientific, siRNA ID s7239, UGGUAUCUCCUCUAAGUAAtt, and siRNA ID s7240, CGAGCAAGGUCCUUCCAUAtt). The combination of the above two siRNAs was delivered into hCMs using Lipofectamine RNAiMAX reagent (Thermo Fisher Scientific, 13778). Silencer Select negative control No. 1 siRNA-treated (Thermo Fisher Scientific, 4390844) cells were used as control. *IGFBP7* mRNA knockdown in hCMs was confirmed by qRT–PCR. Forty-eight hours after siRNA treatment, the treatment was started first by replacing the complete medium with basic myocyte growth medium without supplements for 4 hours, followed by induction with IGF-1 (100 ng ml^−1^) (R&D Systems, 291-G1-200) or insulin (4 μg ml^−1^) (MilliporeSigma, 91007 C) for 15 minutes. Where indicated, a pre-treatment step with IGF1R/InsR inhibitor BMS-754807 (500 nM) (MilliporeSigma, BM0003-5MG) was added 1 hour before induction. Same as above, two Silencer Select validated siRNAs specific to human *IGF1R* (Thermo Fisher Scientific, siRNA ID s7212, CCGAAGAUUUCACAGUCAAtt, and siRNA ID s7211, GCAUGGUAGCCGAAGAUUUtt) were used to knockdown *IGF1R* in AC16 cardiomyocytes.

### Construction of human IGFBP7 expression plasmid and recombinant adenovirus

To generate IGFBP7 expression plasmid, WT human IGFBP7 CDS (NCBI Reference Sequence: NM_001553.3) and truncated mutant forms as illustrated in Extended Data Fig. 5a with 6xHis epitope tag added to the 3′ end were generated by GeneArt gene synthesis and subcloned into pcDNA3.4 TOPO TA cloning vector (Thermo Fisher Scientific) to make plasmid pcDNA3.4-hIGFBP7-6xHis. IGFBP7 constitutive expression recombinant adenovirus vector was further constructed via In-Fusion HD PCR Cloning technology using the AdenoX system 3 kit (Takara Bio, 632267). In brief, WT and mutant IGFBP7 cDNA were PCR-amplified with 15-bp extensions that are homologous to the ends of the linearized adenoviral vector using the corresponding pcDNA3.4-IGFBP7 as a template. The PCR product was then spin-column-purified and mixed with the linearized pAdenoX-ZsGreen1 adenoviral vector in an In-Fusion reaction. After the reaction, a portion of the mixture is transformed into *Escherichia coli* (Stellar Competent Cells; Takara Bio, 636766) and screened. Once a PCR-positive clone is identified, the recombinant pAdenoX-IGFBP7 plasmids were amplified and purified with NucleoBond Xtra Plasmid Midi Kit (Takara Bio, 740410). After confirmation by restriction digestion and sequence analysis, the recombinant pAdenoX-IGFBP7 plasmids were subsequently linearized with the restriction enzyme PacI (New England Biolabs, R0547) and then transfected into Adeno-X 293 cells (Takara Bio, 632271) for viral rescue and amplification. Before moving to high-titer stock preparation, the presence of each gene-specific recombinant construct was verified by PCR and western blotting with anti-6xHis epitope tag antibody (Thermo Fisher Scientific, MA1-21315). To determine fluorescence-based infectivity titers, adenoviral stocks were serially diluted (ten-fold) and applied to HEK293 cells. After 48 hours, fluorescent cells were scored with a fluorescence microscope. The final adenoviral copy numbers in each stock were determined using the Adeno-X qPCR Titration Kit (Takara Bio, 632252).

### Generation of recombinant human IGFBP7 protein

High-yield recombinant human IGFBP7 proteins with biological activity were generated using Invitrogen’s Expi293 Expression System. In brief, after establishing cell suspension culture of Expi293F cells, pcDNA3.4-hIGFBP7-6xHis plasmid was transfected into Expi293F cells using the Expi293 Expression System Kit (Thermo Fisher Scientific, A14635) according to the standard protocol. Expression of secreted IGFBP7-6xHis recombinant protein in culture media was monitored daily by immunoblotting with anti-6xHis epitope tag antibody (Thermo Fisher Scientific, MA1-21315). Six days after transfection, cell culture media containing secreted IGFBP7 recombinant protein was collected, purified and concentrated by using Amicon Ultra centrifugal filter units (Sigma-Aldrich, Z706345), and concentration of the purified IGFBP7 recombinant protein was measured using a pre-commercial Elecsys assay (Roche Diagnostics) on a Cobas e411 platform. The purified recombinant IGFBP7 protein was used to test the binding efficiency of multiple anti-IGFBP7 antibodies in a cell-free system.

### Immunoprecipitation

Isolation and pulldown of histidine-tagged IGFBP7 protein were performed with Dynabeads His-Tag Isolation & Pulldown Assay (Thermo Fisher Scientific, 10104D), following the standard user guide. In brief, primary hCM or AC16 cells were infected with IGFBP7 recombinant adenovirus for 48 hours, followed by treatment as indicated in each figure legend. Treated cells were then lysated with pulldown buffer and mixed with Dynabeads to form the beads–protein complex. After several washes, histidine-tagged IGFBP7 protein and its interacting proteins were eluted with His-Elution Buffer and subjected to downstream immunoblotting analysis with antibodies indicated in each corresponding figure legend. IR and its binding complex in the cell lysate were pulled down by INSR mAb (18–44) (Thermo Fisher Scientific, MA1-10865) with Dynabeads Protein G Immunoprecipitation Kit (Thermo Fisher Scientific, 10007D), following the standard user guide, eluted by adding 1× Bolt LDS sample buffer (Thermo Fisher Scientific, B0008) and subjected to downstream immunoblotting analysis with antibodies indicated in the corresponding figure legend.

### Cellular senescence assay

Cellular senescence assay was performed in both hCMs and mNCMs. hCMs cultured on Millicell EZ slides (Millipore, PEZQS0416) were first treated with IGFBP7 siRNA or control siRNA as described in the siRNA section. Twenty-four hours after siRNA treatment, Dox was added to the culture media with a final concentration at 1 μM for an additional 48 hours. After washing with PBS, cells were fixed and stained with the Cellular Senescence Assay Kit (Millipore, KAA002) to detect the induction of senescence-associated β-galactosidase activity, following the standard procedure provided by the manufacturer, and the images were taken using Nikon light microscopy. Similarly, *Igfbp7*^*−/−*^ and WT mNCMs were treated with Dox for 7 days before proceeding to Cellular Senescence Assay.

### Statistics and reproducibility

Human-biomarker-related large clinical data were analyzed using SAS version 9.4 software (SAS Institute). For continuous variables, the summary data are presented as mean ± s.d., and overall *P* values were calculated using ANOVA. For categorical variables, the values are presented as counts (% of total), and overall *P* values were calculated using a Fisher exact test or a chi-square test when appropriate. For control versus HFpEF and HFpEF versus HFrEF, a Bonferroni correction was used to calculate the *P* values. *P* < 0.05 was considered statistically significant. Statistical analyses for all the other data were conducted using GraphPad Prism version 9 (GraphPad Software). Comparisons between multiple groups of continuous variables were assessed with one-way ANOVA with Bonferroni correction for multiple comparisons. Unpaired two-tailed Studentʼs *t*-tests were used to compare two groups of continuous variables. Details of the statistical tests used are indicated in the respective figure legends. All values are presented as mean ± s.e.m.; *n* refers to the sample size. *P* < 0.05 was considered statistically significant. All micrographs shown are representative of at least two independently repeated experiments. All experiments were repeated independently with similar results.

### Reporting summary

Further information on research design is available in the Nature Portfolio Reporting Summary linked to this article.

### Supplementary information

Supplementary Information Supplementary Tables 1–6

Reporting Summary

### Source data

Source Data Fig. 2 Unprocessed western blots for Fig. 2

Source Data Fig. 3 Unprocessed western blots for Fig. 3

Source Data Fig. 4 Unprocessed western blots for Fig. 4

Source Data Fig. 5 Unprocessed western blots for Fig. 5

Source Data Fig. 6 Unprocessed western blots for Fig. 6

Source Data Fig. 8 Unprocessed western blots for Fig. 8

Source Data Extended Data Fig. 2 Unprocessed western blots for Extended Data Fig. 2

Source Data Extended Data Fig. 4 Unprocessed western blots for Extended Data Fig. 4

Source Data Extended Data Fig. 7 Unprocessed western blots for Extended Data Fig. 7

## Figures and Tables

**Extended Data Table. 1:**
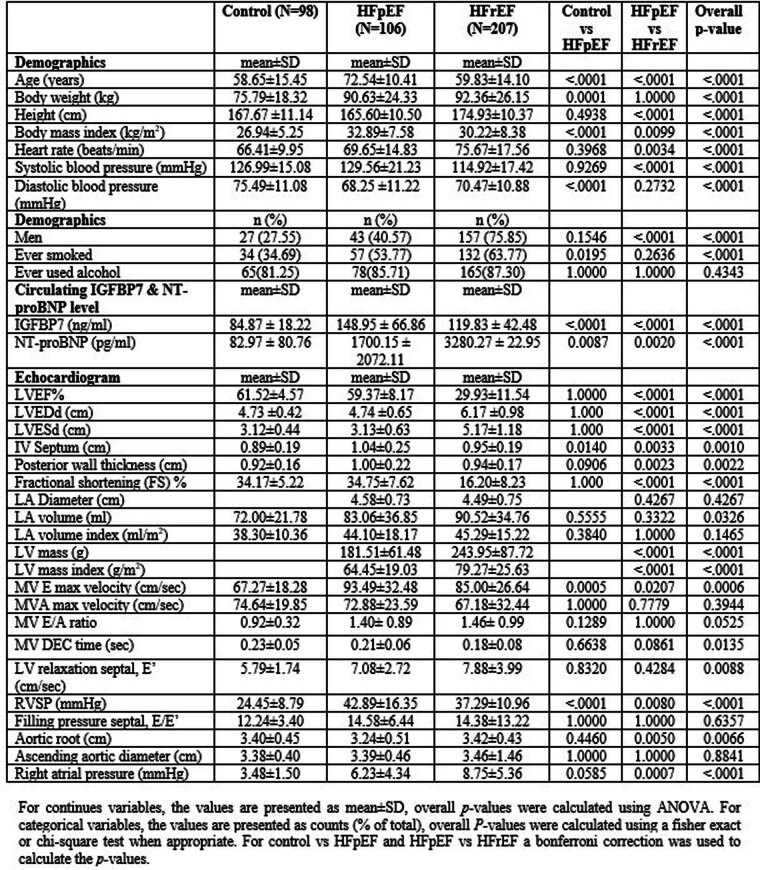
Demographics, circulating IGFBP7 and NT-proBNP levels and echocardiogram measurements by types of HF

**Extended Data Table. 2:**
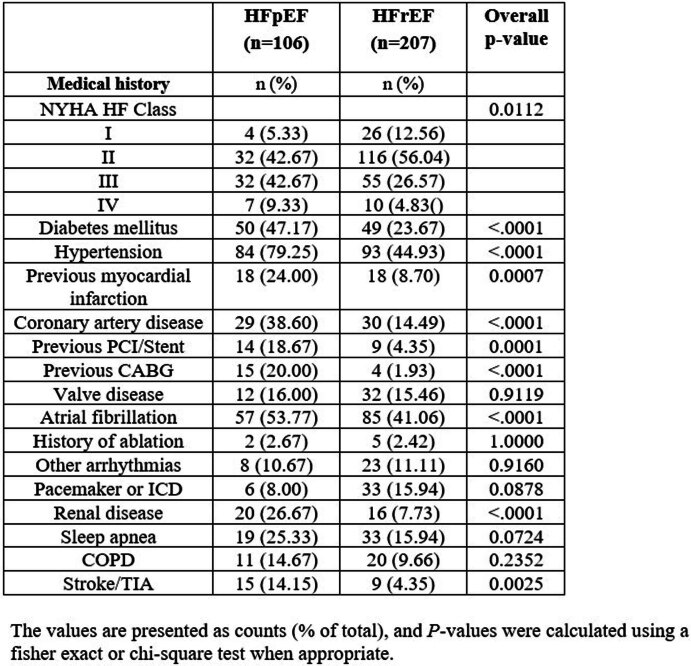
Medical history by types of HF

## Data Availability

All data supporting the findings in this study are included in the main article and associated files. Source data are provided with this paper.
