# Supplementary material for: Insulin-like growth factor-binding protein-7 (IGFBP7) links senescence to heart failure
Source: Nat Cardiovasc Res. 2022 Dec 22;1(12):1195–214. doi: 10.1038/s44161-022-00181-y (PMC11358005; doi:10.1038/s44161-022-00181-y)
Supplement: Supplementary file 1 — Supplementary Tables 1–6 [file 44161_2022_181_MOESM1_ESM.pdf]

nature cardiovascular research

Article https://doi.org/10.1038/s44161-022-00181-y

Insulin-like growth factor-binding protein-7 (IGFBP7) links senescence to heart failure

In the format provided by the authors and unedited

Supplementary Tables 1-6

Supplementary Table 1. Plasma samples used for SomaScan

Supplementary Table 2. Blood samples used for q-RT-PCR studies

Supplementary Table 3. Tissue morphometry, echocardiographic and PV analysis of *Igfbp7*$^{-/-}$ and *Igfbp7*$^{+/+}$ mice at 8 weeks post TAC and sham operation

Supplementary Table 4. Tissue morphometry and echocardiographic analysis of CD-1 mice at 4 weeks post TAC plus *aav9-shRNA* injection

Supplementary Table 5. Tissue morphometry, echocardiographic and PV analysis of C57BL/6 mice at 4 weeks post TAC plus antibody injection

Supplementary table 6. Primer Sequences (5’ to 3’) used for RT-qPCR

Supplementary Table 1. Plasma samples used for SomaScan

Subject ID	Age	Sex	Body weight kg	Height cm	BMI	Heart rate	BPS mmHg	BPD mmHg	LVEF (%)	NYHA class	IGFBP7 ng/ml	proBNP II pg/ml
**Controls**												
04-01-003	55	F	79.37	173.7	26.3	59	149	82			93.75	91.7
04-01-004	63	F	71.8	169	25.1	60	129	81			92.06	135.6
04-01-006	61	F	53	158.5	21.1	75	130	69			74.38	121.3
04-01-014	67	M	67.5	161	26	67	133	72			65.61	65.55
04-01-015	51	M	86.8	178.5	27.2	51	147	91			70.41	23.76
04-01-017	66	F	63.9	156	26.3	70	123	84			75.15	9.03
04-01-019	55	M	102	173	34.1	75	148	104			83.58	26.14
04-01-025	76	M	76	171	26.5	44	140	82			95.3	16.33
04-01-033	60	M	72.5	178	23	60	122	78			90.96	41.07
**HFpEF**												
01-01-056	60	F	157.6	180	48.64	70	108	62	65	2	318	483.2
01-01-078	58	F	106	157	43	68	150	92	50	2	91.93	154.3
01-01-085	70	M	83.7	178	26.42	80	150	78	45	2	234.6	2203
01-01-109	79	M	87	172.7	29.17	50	120	80	50	2	121.5	499.2
01-01-119	71	F	77	155	32.05	82	120	62	50	2	69.47	145.5
01-01-129	84	F	63	165	23.14	96	142	80	77	2	118.1	828.5
01-01-147	68	F	97	155	40.37	72	130	70	57	3	154.1	304.9
01-01-148	79	F	78	157	31.64	100	124	60	55	3	85.03	562.3
01-01-149	59	M	86.6	172.5	29.1	54	118	68	63	3	160.6	1644
01-01-157	78	F	72.6	162	27.66	80	118	68	62	2	126.8	1310
01-01-166	75	F	99.3	160	38.79	60	131	71	63	2	115.9	116.2
01-01-194	86	M	60	162.5	22.72	60	150	70	50	3	199	1842
01-01-198	72	M	101	185.5	29.35	70	108	62	48	3	112.3	4018
**HFrEF**												
01-01-001	71	F	64	165	23.51	64	122	80	16	2	145.5	1033
01-01-009	60	M	62	173	20.72	51	99	57	47	2	107.6	580.5
01-01-018	53	M	78	175	25.47	69	84	58	15	2	112.5	789.3
01-01-026	75	M	80.9	182	24.42	70	122	80	25	2	191.4	2778
01-01-086	80	M	72.5	180	22.38	60	104	60	26	2	106.2	4273
01-01-128	23	M	68	175	22.2	93	102	69	8	4	81.69	4771
01-01-184	72	M	65.7	175.5	21.33	70	120	64	39	2	91.36	845.8
01-01-187	52	M	95.8	170	33.15	99	99	66	15	4	120.2	2242
01-01-203	26	M	98.6	193	26.47	101	146	67	16	3	121.2	4815
01-01-207	84	F	61.8	168	21.9	100	102	58	28	2	122.9	1989
01-01-216	41	F	65.7	167.5	23.42	99	136	94	17	3	162.7	2491
01-01-221	68	M	71	178	22.41	62	116	79	25	2	131	3051
01-01-233	63	F	107.5	155	44.75	60	180	70	27	2.5	183.1	3266
01-01-040	56	F	56.8	163	21.38	68	80	60	8	1	78.02	3427
01-01-007	63	M	93	173	31.07	75	130	80	32	2	96.06	1502
01-01-014	55	F	92.3	157	37.45	60	98	50	25	3	187	3903
01-01-045	87	F	56.4	160	22.03	55	150	60	33	3	97.74	272.6
01-01-064	79	F	58.9	157	23.9	65	110	60	31	2	108.5	3712
01-01-106	72	M	116	180	35.8	58	128	80	19	2	104.2	1744
01-01-134	89	M	75.5	170	26.12	74	88	56	33	4	110.3	3795
01-01-142	53	M	118.4	177.8	37.45	88	104	70	26	2	81.1	741.3
01-01-146	50	M	136	170	47.06	80	150	80	27	3	100.5	154
01-01-153	54	M	106	177	33.83	64	130	80	33	2	81.02	141.7
01-01-159	77	M	59	168	20.9	88	139	62	25	3	116.4	1829
01-01-192	61	M	60	170	20.76	65	122	62	18	2	77.39	2470
01-01-220	73	M	83.9	191	23	75	82	50	27	3	121	2877

Supplementary Table 2. Blood samples used for q-RT-PCR studies

Subject ID	Age	Sex	Body weight kg	Height cm	BMI	heart rate	BPS mmHg	BPD mmHg	LVEF %	NYHA class	IGFBP7 ng/ml	proBNP II pg/ml
**Controls**												
04-01-014	67	M	67.5	161	26	67	133	72			65.61	65.55
04-01-017	66	F	63.9	156	26.3	70	123	84			75.15	9.03
04-01-018	68	F	70.9	158.5	28.2	72	140	83			73.8	72.97
04-01-021	60	F	87	158.7	34.5	79	128	78			80.09	13.8
04-01-025	76	M	76	171	26.5	44	140	82			95.3	16.33
04-01-028	72	F	56	150	24.9	75	147	83			88.85	57.46
04-01-032	75	F	58	160	22.7	64	125	74			87.6	75.48
04-01-037	81	F	52	160	20.3	63	138	84			84.7	258
04-01-038	65	F	53.5	170	18.5	62	125	75			87.84	36.97
04-01-041	65	M	84	188	23.8	65	133	79			96.13	67.42
04-01-042	45	M	80	182	24.2	68	121	78			80.15	57.69
04-01-046	59	M	50	156	20.5	85	126	77			81.14	31.23
04-01-056	82	M	58	167.5	20.7	59	116	61			128.3	99.51
04-01-060	40	F	63.5	166	23	73	101	73			89.67	55.69
04-01-061	71	F	67	152.5	28.8	58	122	68			69.95	68.73
**HFpEF**												
01-01-020	68	F	71	165	26.08	81	110	55	55	2	241.6	1187
01-01-030	68	F	79.2	158	31.73	54	102	52	50	3	341.1	3897
01-01-056	60	F	157.6	180	48.64	70	108	62	65	2	318	483.2
01-01-080	40	F	108.7	168	38.51	98	132	78	61	3	243.5	232
01-01-085	70	M	83.7	178	26.42	80	150	78	45	2	234.6	2203
01-01-149	59	M	86.6	172.5	29.1	54	118	68	63	3	160.6	1644
01-01-191	79	M	115	183	34.34	65	122	62	55	2	247	5380
01-01-239	72	F	71.6	162.5	27.11	60	144	64	74	2	200.6	4398
01-01-242	65	M	117.5	183	35.09	60	142	62	58	3	168	762.9
01-01-270	46	M	70.5	165	25.9	68	94	59	59	3	345.6	1832
01-01-281	72	M	69	175.5	22.4	54	109	64	50	2	266.5	724.9
01-01-289	66	F	120.8	170	41.8	116	100	58	65	3	276	3102
01-01-290	79	F	91.6	155	38.13	78	120	70	71	3	396.2	9708
01-01-296	82	M	77.5	178	24.46	55	110	78	52	3	370.8	2379
01-04-041	66	F	132.4	161	51.08	61	123	80	58	3	159.2	478.3
**HFrEF**												
01-01-156	66	M	72.8	178	22.98	67	100	65	20	2	78.1	14736
01-01-189	54	M	76.6	175.5	24.87	71	120	54	39	3	102.8	6554
01-01-212	62	M	146.5	166	53.16	75	94	60	27	3	102.8	8091
01-01-260	69	F	72.5	172.5	24.36	115	115	45	26	3	84.54	6709
01-01-276	48	M	66	178	20.83	61	75	56	10	3	154.9	20905
01-01-286	40	M	100.6	188	28.46	66	88	59	10	3	186.3	23608
01-02-007	80	F	59	170	20.42	68	110	60	19	4	133.8	8065
01-01-032	74	M	89.9	172	30.39	81	125	61	40	3	135.9	28793
01-01-055	85	M	66	165	24.24	65	144	55	38	3	347.2	17230
01-01-246	80	F	63.2	165	23.21	90	86	62	20	3	66.12	5312
01-01-256	73	F	63	157.5	25.4	100	142	108	31	4	139.6	8299
01-01-257	63	M	77.9	172.5	26.18	68	117	68	30	3	83.03	6362
01-01-306	63	F	78.4	170	27.13	76	108	62	22	4	154.6	70000
01-02-001	65	M	73.1	170	25.29	80	116	66	33	3	130.1	7979
01-02-014	69	F	71.4	163	26.87	76	102	50	22.5	1	117.7	4880
01-04-021	81	M	82.2	170	28.44	86	103	64	50	3	147.1	19946

Supplementary Table 3. Tissue morphometry, echocardiographic and PV analysis of *Igfbp7*$^{-/-}$ and *Igfbp7*$^{+/+}$ mice at 8 weeks post TAC and sham operation

At 8 weeks post-surgery	*Igfbp7*$^{+/+}$ sham	*Igfbp7*$^{+/+}$ TAC	*Igfbp7*$^{-/-}$ sham	*Igfbp7*$^{-/-}$ TAC
**Tissue morphometry**	n=30	n=31	n=30	n=38
heart weight, mg	206.59±3.48	280.77±14.68**	131.23±3.10	155.83±5.71
Lung weight, mg	253.65±10.98	352.86±31.94**	179.92±65.15	178.30±3.13
Body weight, g	44.94±0.91	40.54±0.89*	32.60±0.95	30.61±0.71
Tibia length, mm	21.79±0.26	21.64±0.24	20.67±0.15	20.71±0.21
HW/BW, mg/g	4.63±0.09	7.05±0.42****	4.08±0.10	5.14±0.20
LW/BW, mg/g	5.74±0.30	9.12±1.04**	5.57±0.14	5.90±0.13
HW/TL, mg/mm	9.52±0.19	13.08±0.77****	6.38±0.18	7.55±0.29
LW/TL, mg/mm	11.55±0.41	16.43±1.58***	8.73±0.27	8.64±0.18
**Echocardiography**	n=15	n=16	n=7	n=13
LVESD (mm)	3.11±0.14	3.60±0.14****	2.43±0.10	2.46±0.07
LVEDD (mm)	4.48±0.09	4.52±0.11****	3.74±0.15	3.67±0.07
EF (%)	57.56±3.33	41.23±3.36****	64.65±2.05	61.66±2.15
LV Mass (corrected) (mg)	176.52±16.07	256.21±22.50****	138.86±17.30	134.52±10.61
A’ (mm/s)	-16.03±2.10	-13.07±1.60*	-13.74±1.11	-16.76±1.41
E’ (mm/s)	-21.21±2.19	-19.28±2.46*	-21.53±0.67	-23.90±1.90
MV A (mm/s)	603.49±60.8	677.93±44.31	541.35±21.76	602.55±42.76
MV E (mm/s)	752.96±71.42	872.31±46.92	719.69±4.55	794.57±47.12
IVRT	12.77±1.95	24.12±2.41*	13.27±1.22	16.88±1.06
IVCT	15.25±1.19	20.59±2.01	15.02±1.22	17.51±0.62
MV E/e’	-37.63±3.52	-54.34±7.61*	-33.62±1.26	-35.41±3.90
**Invasive hemodynamics**)	n=10	n=11	n=8	n=16
HR (bpm)	618±8.04	587±13.56	580±13.08	574±6.06
Arterial elastance (Ea)	4.90±0.27	6.53±1.35	5.17±0.55	6.05±0.67
dP/dt max (mmHg/sec)	9074±419	6931±463	9201±1086	7322±343
dP/dt min (mmHg/sec)	-7982±368	-6184±472	-7614±959	-7035±348
EF (%)	79.42±3.15	59.47±5.25*	75.54±2.95	74.07±2.08
LVEDP (mmHg)	9.17±0.67	13.79±1.41*	8.27±0.82	10.05±0.65

LVESP (mmHg)	82.29±3.57	100±4.67	80.13±6.40	98.49±4.48
Tau (Weiss' method) (ms)	6.26±0.35	8.88±0.59*	6.13±0.56	7.38±0.24

n = number of mice per group; Sham = sham-operated; TAC = transaortic constriction; LVEDD = left ventricular (LV) end-diastolic dimension; LVESD = LV end-systolic dimension; EF (%) = LV ejection fraction; MV A = mitral valve A velocity; MV E = mitral valve E velocity; A' = velocity at A', E' = velocity at E'; IVRT = isovolumic relaxation time; IVCT = isovolumic contraction time; MV E/e' = ratio of the PW doppler derived mitral valve velocity at E to tissue doppler derived velocity at E'; LVEDP = LV end-diastolic pressure; LVESP = LV end-systolic pressure; HR = heart rate; Tau = the time constant of isovolumic pressure decay. Values are presented as mean ± s.e.m., one-way ANOVA with Tukey's correction for multiple comparisons were used to calculate *p* values. Statistical significance is represented as $^{*}p < 0.05$, $^{**}p < 0.01$, $^{***}p < 0.001$ and $^{****}p < 0.0001$, comparing *Igfbp7*$^{-/-}$ *TAC* to *Igfbp7*$^{+/+}$ *TAC*.

Supplementary Table 4. Tissue morphometry and echocardiographic analysis of CD-1 mice at 4 weeks post TAC plus *aav9-shRNA* injection

**At 4 weeks post-treatment**	**TAC+ *aav9-mcherry-U6-mIgfbp7-shRNA***	**TAC + *aav9-scrmb-shRNA***
**Tissue morphometry**	n=11	n=9
heart weight, mg	247.21±11.12	268.88±22.26
Lung weight, mg	379.44±21.97	469.83±38.63*
Body weight, g	44.21±1.70	41.99±0.71
Tibia length, mm	22.671±0.14	22.56±0.18
HW/BW, mg/g	5.63±0.24	6.40±0.51
LW/BW, mg/g	8.69±0.55	11.18±0.88*
HW/TL, mg/mm	10.90±0.46	11.90±0.94
LW/TL, mg/mm	16.72±0.92	20.78±1.60*
**Echocardiography**	n=11	n=9
LVESD (mm)	3.53±0.10	3.52±0.20
LVEDD (mm)	4.76±0.08	4.64±0.13
EF (%)	48.84±2.84	34.39±3.71**
A' (mm/s)	-22.88±1.95	-29.72±3.18
E' (mm/s)	-31.48±2.48	-37.19±3.56
MV A (mm/s)	650.23±30.69	775.26±40.24*
MV E (mm/s)	901.66±33.54	941.43±67.44
IVRT	16.71±0.42	22.11±1.79**

IVCT	10.55±0.32	12.98±0.94****
MV E/e’	-30.93HW±2.90	-28.43±5.03
MV E/A	1.40±0.06	1.21±0.06*

n = number of mice per group; TAC = transaortic constriction; LVEDD = left ventricular (LV) end-diastolic dimension; LVESD = LV end-systolic dimension; EF (%) = LV ejection fraction; MV A = mitral valve A velocity; MV E = mitral valve E velocity; A’ = velocity at A’, E’ = velocity at E’; IVRT = isovolumic relaxation time; IVCT = isovolumic contraction time; MV E/e’ = ratio of the PW doppler derived mitral valve velocity at E to tissue doppler derived velocity at E’; MV E/A = mitral valve E to A ratio. Values are presented as mean ± s.e.m., Unpaired two-tailed *t-test* was used to calculate *p* value. Statistical significance is represented as $^{*}p < 0.05$, $^{**}p < 0.01$, $^{***}p < 0.001$ and $^{****}p < 0.0001$.

Supplementary Table 5. Tissue morphometry, echocardiographic and PV analysis of C57BL/6 mice at 4 weeks post TAC plus antibody injection

**At 4 weeks post-treatment**	**TAC+ IGFBP7 antibody (Clone 65)**	**TAC+ Control IgG**
**Tissue morphometry**	n=14	n=14
heart weight, mg	166.44±8.98	199.61±11.64*
Lung weight, mg	150.32±11.28	183.98±23.39
Body weight, g	26.56±0.94	26.83±0.97
Tibia length, mm	21.21±0.11	21±0
HW/BW, mg/g	6.28±0.28	7.56±0.52*
LW/BW, mg/g	5.32±0.18	7.09±0.96
HW/TL, mg/mm	7.84±0.42	9.51±0.55*
LW/TL, mg/mm	6.57±0.17	8.97±1.18*
**Echocardiography**	n=15	n=14
LVESD (mm)	3.57±0.12	3.98±0.11*
LVEDD (mm)	4.53±0.09	4.71±0.10*
EF (%)	43.19±2.52	32.66±2.51*
A’ (mm/s)	-22.29±2.08	-23.39±1.83
E’ (mm/s)	-32.53±2.63	-34.97±3.02
MV A (mm/s)	752.22±31.40	839.25±28.63*
MV E (mm/s)	962.68±36.02	990.14±25.39

IVRT	16.73±0.41	19.89±0.37****
IVCT	10.45±0.37	11.68±0.39*
MV E/e'	-34.96±5.61	-34.53±6.04
MV E/A	1.29±0.04	1.18±0.02*
**Invasive hemodynamics**	n=9	n=10
Heart rate (HR) (bpm)	559.79±9.60	589.93±3.05
Arterial elastance (Ea) (mmHg/ml)	6.65±0.92	7.26±1.30
dP/dt max (mmHg/sec)	6802±363	7655±299
dP/dt min (mmHg/sec)	-6081±452	-6558±467
EF (%)	54.79±2.63	58.70±3.50
LVEDP (mmHg)	7.31±0.72	12.75±1.86*
LVESP (mmHg)	104.13±4.71	114.75±2.71
Tau (Weiss' method) (ms)	7.46±0.17	8.81±0.55*

n = number of mice per group; TAC = transaortic constriction; LVEDD = left ventricular (LV) end-diastolic dimension; LVESD = LV end-systolic dimension; EF (%) = LV ejection fraction; MV A = mitral valve A velocity; MV E = mitral valve E velocity; A' = velocity at A', E' = velocity at E'; IVRT = isovolumic relaxation time; IVCT = isovolumic contraction time; MV E/e' = ratio of the PW doppler derived mitral valve velocity at E to tissue doppler derived velocity at E'; MV E/A = mitral valve E to A ratio; LVEDP = LV end-diastolic pressure; LVESP = LV end-systolic pressure; HR = heart rate; Tau = The time constant of isovolumic pressure decay. Values are presented as mean ± s.e.m., Unpaired two-tailed *t-test* was used to calculate *p* value. Statistical significance is represented as $^{*}p < 0.05$, $^{**}p < 0.01$, $^{***}p < 0.001$ and $^{****}p < 0.0001$.

Supplementary table 6. Primer Sequences (5’ to 3’) used for RT-qPCR

Primer Pairs Used for RT-qPCR	
Human IGFBP7: Primer Pair 1	
actggctgggtgctggta	tggatgcatggcactcata
Human IGFBP7: Primer Pair 2	
gaatcccgacacctgtcctc	cagcacccagccagttactt
Human HPRT1	
tgacactggcaaaacaatgca	ggtccttttcaccagcaagct
Human NPPB	
caccgcaaaatggtcctcta	gtccatcttcctcccaaagc
Human TP53	
cagcacatgacggaggttgt	tcatccaaatactccacacgc
Human CDKN1A (p21)	
tgtccgtcagaacccatgc	Aaagtcgaagttccatcgctc
Mouse Nppa	
gcttccaggccatattggag	gggggcatgacctcatctt
Mouse Nppb	
gaggtcactcctatcctctgg	gccatttcctccgacttttctc
Mouse Trp53	
cacgtactctcctcccctcaat	aactgcacagggcacgtctt
Mouse Cdkn1a	
Bio-Rad PrimerPCR SYBR Green Assay:	qMmuCED0046265
Mouse Gadd45a	
Bio-Rad PrimerPCR SYBR Green Assay:	qMmuCED0001074
Mouse Ddb1	

Bio-Rad PrimerPCR SYBR Green Assay:	qMmuCED0045622
Mouse Sod2	
Bio-Rad PrimerPCR SYBR Green Assay:	qMmuCED0006109
Mouse Cdkn1b	
Bio-Rad PrimerPCR SYBR Green Assay:	qMmuCED0037679
Mouse Cat	
Bio-Rad PrimerPCR SYBR Green Assay:	qMmuCED0011237
Mouse Myh6	
tgcactacggaaacatgaagtt	cgatggaatagtacacttgctgt
Mouse Myh7	
actgtcaacacttaagagggtca	ttggatgatttgatcttccaggg
Mouse Il-6	
cgagcccaccaggaacgaaagtc	ctggctggaagtctcttgcggag
Mouse Il-1b	
ccctgcagctggagagtgtgg	tgtgctctgcttgagaggtgct
Mouse Ctgf	
caaagcagctgcaaatacca	ggccaaatgtgtcttccagt
Mouse Tgfb	
caacaacccctttgccaaag	tcccccaagcagttgacagt
Mouse Hprt1	
gctgacctgctggattacat	ttggggctgtactgcttaac
