# Supplementary material for: Insulin-like growth factor-binding protein-7 (IGFBP7) links senescence to heart failure
Source: Nat Cardiovasc Res. 2022 Dec 22;1(12):1195–214. doi: 10.1038/s44161-022-00181-y (PMC11358005; doi:10.1038/s44161-022-00181-y)
Supplement: Supplementary file 2 — Reporting Summary [file 44161_2022_181_MOESM2_ESM.pdf]

nature portfolio

Corresponding author(s): Peter P. Liu; NCVR-2021-08-0214B

Last updated by author(s): Oct 28, 2022

Reporting Summary

Nature Portfolio wishes to improve the reproducibility of the work that we publish. This form provides structure for consistency and transparency in reporting. For further information on Nature Portfolio policies, see our Editorial Policies and the Editorial Policy Checklist.

Statistics

For all statistical analyses, confirm that the following items are present in the figure legend, table legend, main text, or Methods section.

n/a	Confirmed	
☐	☒	The exact sample size (*n*) for each experimental group/condition, given as a discrete number and unit of measurement
☐	☒	A statement on whether measurements were taken from distinct samples or whether the same sample was measured repeatedly
☐	☒	The statistical test(s) used AND whether they are one- or two-sided<br>*Only common tests should be described solely by name; describe more complex techniques in the Methods section.*
☐	☒	A description of all covariates tested
☐	☒	A description of any assumptions or corrections, such as tests of normality and adjustment for multiple comparisons
☐	☒	A full description of the statistical parameters including central tendency (e.g. means) or other basic estimates (e.g. regression coefficient) AND variation (e.g. standard deviation) or associated estimates of uncertainty (e.g. confidence intervals)
☐	☒	For null hypothesis testing, the test statistic (e.g. *F*, *t*, *r*) with confidence intervals, effect sizes, degrees of freedom and *P* value noted<br>*Give P values as exact values whenever suitable.*
☒	☐	For Bayesian analysis, information on the choice of priors and Markov chain Monte Carlo settings
☒	☐	For hierarchical and complex designs, identification of the appropriate level for tests and full reporting of outcomes
☒	☐	Estimates of effect sizes (e.g. Cohen's *d*, Pearson's *r*), indicating how they were calculated

Our web collection on statistics for biologists contains articles on many of the points above.

Software and code

Policy information about availability of computer code

Data collection	Cobas e411 analyzer (Roche Diagnostics) was used for biomarker data collection. SOMAscan proteomics (SomaLogic) was used to map SASP changes in plasma samples and analyzed using SomaSuite ver. 1.0.20120704. VisualSonic Vevo 2100 and 3100 with VevoLab 5.6.0 by FUJIFILM VisualSonics were used for echocardiography data collection and analysis. Millar Pressure-Volume System (MPVS) with LabChart 8.0 by ADInstruments was used to acquire PV loop data and analysis. The Leica Aperio Versa 8 or EVOS M5000 Cell Imaging System and software (ThermoFisher, AMF5000) was used to scan immunohistochemistry slides and analyzed using Aperio ImageScope v12.4.3. , while the Olympus FluoView 1000 Laser Scanning Confocal Microscope or Zeiss Elyra S.1 LSM 880 Airyscan Confocal Microscope was used for immunofluorescent image data collection and analyzed using FLOUVIEW FV10-ASW 4.2 Viewer or Aperio ImageScope viewing v12.4.(Leica Biosystems) . The intensities of the chemiluminescence signals of the immunoblots were detected using the ChemiDoc XRS+ System (Bio-Rad, 1708265) and quantified using Image Lab software (version 6.1; Bio-Rad). LightCycle96 with software LightCycle96 SW 1.1 (Roche Diagnostics) was used for qPCR data collection and analysis. PerkinElmer EnVision multimode plate reader with EnSpire Manager software v2.00 was used for ELISA data collection and analysis, while for Multiplex ELISA data, Meso Scale Discovery's U-PLEX platform was used as per Manufacturer's protocols with MESO QuickPlex SQ 120 Instrument and Discovery Workbench V 4.0. software.
Data analysis	Human biomarker related large clinical data were analysis using SAS/9.4 software (SAS Institute Inc.). For continues variables, the values are presented as mean±SD, overall p-values were calculated using ANOVA. For categorical variables, the values are presented as counts (% of total), overall P-values were calculated using a fisher exact or chi-square test when appropriate. For control vs HFpEF and HFpEF vs HFrEF a bonferroni correction was used to calculate the p-values. P values of P<0.05 was considered statistically significant.<br><br>Statistical analyses for all the other data were conducted using GraphPad Prism software V9 (GraphPad Software Inc.). Comparisons between multiple groups of continuous variables were assessed with one-way ANOVA with Tukey's correction for multiple comparisons. Unpaired two-tailed Student t-tests were used to compare of two groups of continuous variables. Details of the statistical tests used are indicated in the respective figure legends. All values are presented as means ± s.e.m., n refers to the sample size. P< 0.05 was considered significant. Statistical significance is represented as *p < 0.05, **p < 0.01, ***p < 0.001 and ****p < 0.0001.

ImageScope v12.4.3 (Leica), EVOS M5000 v1.4 (ThermoFisher) and V10-ASW 4.2 Viewer (Olympus Microscope) were used for microscope image processing.

For manuscripts utilizing custom algorithms or software that are central to the research but not yet described in published literature, software must be made available to editors and reviewers. We strongly encourage code deposition in a community repository (e.g. GitHub). See the Nature Portfolio guidelines for submitting code & software for further information.

Data

Policy information about availability of data

All manuscripts must include a data availability statement. This statement should provide the following information, where applicable:

- Accession codes, unique identifiers, or web links for publicly available datasets
- A description of any restrictions on data availability
- For clinical datasets or third party data, please ensure that the statement adheres to our policy

All data supporting the finding in this study are included in the main article and associated files. Source data are provided with this paper.

Field-specific reporting

Please select the one below that is the best fit for your research. If you are not sure, read the appropriate sections before making your selection.

☒ Life sciences ☐ Behavioural & social sciences ☐ Ecological, evolutionary & environmental sciences

For a reference copy of the document with all sections, see nature.com/documents/nr-reporting-summary-flat.pdf

Life sciences study design

All studies must disclose on these points even when the disclosure is negative.

Sample size	Sample sizes for in vivo experiments were based on previously published experiments and experience from the Liu lab, while complying with the 3Rs rule on reducing, replacing and refining the use of animals for scientific purpose. The simple size (n) for each experiment is provided in the figure legends. For in vitro experiments, each treatment is represented by 3 individual replicates.
Data exclusions	Data were not excluded from study reporting. Animals who suffered perioperative (same day) death were not included in study reporting.
Replication	Results shown are representative of several independently performed experiments. All in vitro experiments were repeated at least 3 times from biological replicates, containing at least 3 technical replicates. There were no experiments were not replicated or could not be reproduced.
Randomization	All studies were performed with randomization when possible. For TAC surgery, mice with body weight between 24-26 g were preselected and then were randomly assigned prior to surgery. For cell culture experiments, individual wells were randomly assigned for treatment conditions. Randomization does not apply to human patient samples and immonoblotting experiments, which were placed into groups based on their diagnosis.
Blinding	Surgeon, imaging (echocardiography, PV loop and microscopy) and pathology measurement were blinded. Data analysis was performed independently with animal identification only by numbering (identified by ear notching or tail marking) as assigned prior to experimental procedure. For immunoblotting and qPCR, samples were processed and assays conducted in unblinded fashion.

Reporting for specific materials, systems and methods

We require information from authors about some types of materials, experimental systems and methods used in many studies. Here, indicate whether each material, system or method listed is relevant to your study. If you are not sure if a list item applies to your research, read the appropriate section before selecting a response.

Materials & experimental systems

n/a	Involved in the study	
☐	☒	Antibodies
☐	☒	Eukaryotic cell lines
☒	☐	Palaeontology and archaeology
☐	☒	Animals and other organisms
☐	☒	Human research participants
☒	☐	Clinical data
☒	☐	Dual use research of concern

Methods

n/a	Involved in the study	
☒	☐	ChIP-seq
☒	☐	Flow cytometry
☒	☐	MRI-based neuroimaging

Antibodies

Antibodies used

For Immunoblots, the following antibodies were used:

IGFBP7 polyclonal antibody (ThermoFisher PA1-86872, 1:1000),

anti-IGFBP7 [EPR11913(B)] (abcam ab170932, 1:1000),

Rb mAb to IGFBP3 (ab193910, 1:1000)

CTGF (abcam ab6992, 1:1000),

anti-53BP1 (abcam ab36823, 1:1000),

anti-p21 [EPR18021] (abcam ab188224, 1:1000),

anti-p16ARC [EP1551Y] (abcam ab51243, 1:1000),

anti-Histone H3 (abcam ab1791, 1:1000),

phosppho-p53 (ser392) (Cell Signaling, #9281, 1:1000),

Acetyl-p53 (Lys379) (Cell Signaling, #2570, 1:1000),

p53 (DSHB Hybridoma Product PCRP-TP53-1F7, 0.5?g/ml; PCRP-TP53-1F7 was deposited to the DSHB by Protein Capture Reagents Program, produced by JHU/CDI),

Phospho-IGF-1 Receptor b (Thy1135) (Cell Signaling, #3918, 1:500),
IGF-1 Receptor b (Cell Signaling, #9750, 1:500),

Phospho-IRS-1 (Ser612) (Cell Signaling, #3203, 1:500),

Phospho-IRS-1 (Ser318) (Cell Signaling, #5610, 1:500),

IRS-1 (Cell Signaling, #3407, 1:500),

IRS-1 (Millipore, 06-248, 1:1000),

IRS-2 (Cell Signaling, #4502, 1:1000),

INSR (18-44) (ThermoFisher, MA1-10865, 1:1000),

Phospho-Akt (Ser473) (D9E) (Cell Signaling, #4060, 1:1000),

Phospho-Akt (Thr308) (Cell Signaling, #4056, 1:1000),

Akt (pan) (C67E7) (Cell Signaling, #4691, 1:1000),

phosphor-FOXO3A (S253) [EPR1951(2)] (abcam, ab154786, 1:1000),

FOXO3A (abcam ab109629, 1:1000),

Insulin receptor b (C18C4) (Enzo ADI-905-683, 1:1000),

anti-cGAS (Cell Signaling, #15102, 1:1000),

anti-STING (Millipore Sigma, MABF270, 1ug/ml),

anti-DDB1 (abcam, ab109027, 1:50000),

anti-SOD2 (acetyl K68) (abcam ab137037, 1:1000),

anti-SOD2 (abcam ab68155, 1:1000).

Blots were incubated with HRP-conjugated Goat anti-mouse IgG (Bio-Rad, 170-5047, 1:100,000), Goat anti-rabbit IgG (Bio-Rad, 170-5046, 1:100,000), or monoclonal mouse anti-goat IgG (Jackson ImmunoResearch, 205-032-176, 1:50,000) and developed using Clarity Western ECL Substrate (Bio-Rad, 170-5061) or SuperSignal West Pico PLUS Chemiluminescent Substrate (ThermoFisher, 34580). To detected low abundant protein, SuperSignal West Femto Maximum Sensitivity Substrate (ThermoFisher, 34095) or SuperSignal West Atto Ultimate Sensitivity Chemiluminescent Substrate (ThermoFisher, A38544) was used. The intensities of the chemiluminescence signals were detected using the ChemiDoc XRS+ System (Bio-Rad, 1708265) and quantified using Image Lab software (version 6.1; Bio-Rad). To normalize signals to total protein, blot membranes were either pre-stained with No-stain protein labelling reagent (Thermo-Fisher, A4449) or stripped and re-probed with antibody against GAPDH (ThermoFisher, MA5-15738, 1:5000), or Vinculin (Millipore Sigma, V4505, 1:1000) and the results were shown as fold change against control.

For immunofluorescent staining:

Anti-IGFBP7 (abcam, ab74169 1:200),
mouse monoclonal anti-vimentin (V1-10) (abcam, ab20346, 1g/ml),
Alexa Flour 568 conjugated Isolectin GS-IB4 (ThermoFisher, I21412).

FM 1-43FX membrane probe (5 µg/mL) (ThermoFisher, F35355),

anti-IGF1 receptor (abcam, ab131476, 1:100),

Alexa Fluor 488 conjugated wheat germ agglutinin (WGA) (1 µg/mL) (ThermoFisher, W11261)

Picro Sirius Red (abcam, ab150681)

Following overnight incubation with primary antibody, the sections were incubated with a matching Alexa Fluor Dyes conjugated

secondary antibody (ThermoFisher, 1:1000) at room temperature for 1 hour, followed by nuclear stain with Hoechst 33342 (1 µg/mL)

(ThermoFisher, WH21492) at room temperature for 10 min and were mounted with Dako fluorescence mounting medium

(AgilentDako, S302380-2).

Validation	Anti-IGFBP7 antibodies (ThermoFisher PA1-86872 and abcam ab170932) for immunoblot were validated using recombinant IGFBP7 protein as a positive control and Igfbp7 KO tissue lysates as negtive control.<br><br>For immunofluorescent staining, anti-IGFBP7 antibody (abcam, ab74169 1:200) was validated using IGFBP7 over-expression AC16 cell as positive control and Igfbp7 KO tissue sections as negative controls.<br><br>For all the other primary antibodies, validation information are available on manufacture's website.

Eukaryotic cell lines

Policy information about cell lines

Cell line source(s)	AC16 human cardiomyocyte cell line (AC16) (Millipore/Sigma, SCC109)<br>Primary human cardiac myocytes (HCM) from PromoCell (C-12810, Lot#463Z016.2). Donor: 35 years old Caucasian female.<br>Primary human cardiac fibroblasts (HCF) from PromoCell (C-12375, Lot#463Z007.1). Donor: 59 years old Caucasian male.<br>Primary human cardiac microvascular endothelial cells (HCMEC) from PromoCell (C-12285, Lot#447Z026.7). Donor: 63 years old Caucasian male.<br>Adeno-X 293 cells (Takara Bio, 632271)<br>Expi293F™ Cells (ThermoFisher, A14527)
Authentication	AC16 cells were verified by Millipore and the following Quality Control Testing were performed by Millipore:<br>• Each vial contains ≥ 1X106 viable cells.<br>• Cells are tested negative for HPV-16, HPV-18, Hepatitis A, B, C, and HIV-1 & 2 viruses by PCR.<br>• Cells are negative for mycoplasma contamination.<br>• Each lot of cells is genotyped by STR analysis to verify the unique identity of the cell line.<br>Each lot of Primary HCM, HCF and HCMEC were verified and certificated by PromoCell with viability and growth characteristics, phenotypic characterization, and tested free from microbiological contaminates and infectious viruses.
Mycoplasma contamination	Cells obtained from the vendor are certified negative for mycoplasma contamination. Primary cells are one time use only. For cell line, only cells in earlier passages were used for the study, no verification for mycoplasma contamination were performed.
Commonly misidentified lines<br>(See ICLAC register)	No commonly misidentified lines were used for this study.

Animals and other organisms

Policy information about studies involving animals; ARRIVE guidelines recommended for reporting animal research

Laboratory animals	Igfbp7-/- mice in CD1 background were kindly provided by Dr. Arun Seth (Sunnybrook Research Institute). CD1 and C57BL/6 mice were from Charles River. All mice are males, age 12-22 weeks old at the start of surgery. Mice were housed at 24 degrees Celsius at 30-60% humidity on a 12h light/12 h dark schedule at the Animal Care and Veterinary Service Facility, University of Ottawa.
Wild animals	No wild animals were used for this study.
Field-collected samples	This study did not involve field-collected samples.

Ethics oversight	All animal experimental protocols were approved by the Animal Care and Use Committee at the University of Ottawa and performed in accordance with institutional guidelines.

Note that full information on the approval of the study protocol must also be provided in the manuscript.

Human research participants

Policy information about studies involving human research participants

Population characteristics	Human population characteristics of the participates was listed on Extended data Table 1 - Demographics of patients enrolled in the study.
Recruitment	Informed consent was obtained from all patients/family members before enrolled into the study.
Ethics oversight	The collection and use of human samples in this study were approved by the Ottawa Health Science Network Research Ethics Board members (Ottawa Health Science Research Board REB number: 20140869-01H)

Note that full information on the approval of the study protocol must also be provided in the manuscript.
