# Supplementary figures and images for: Insulin-like growth factor-binding protein-7 (IGFBP7) links senescence to heart failure
Source: Nat Cardiovasc Res. 2022 Dec 22;1(12):1195–214. doi: 10.1038/s44161-022-00181-y (PMC11358005; doi:10.1038/s44161-022-00181-y)

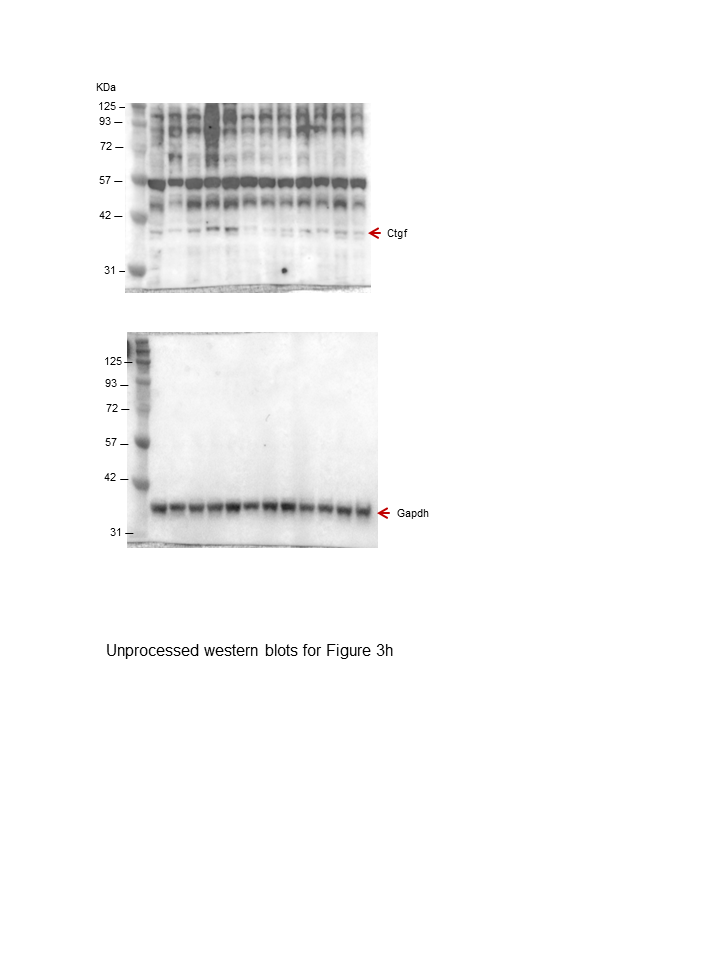

Supplement: Supplementary file 4 — Unprocessed western blots for Fig. 3 [file 44161_2022_181_MOESM4_ESM.tif]

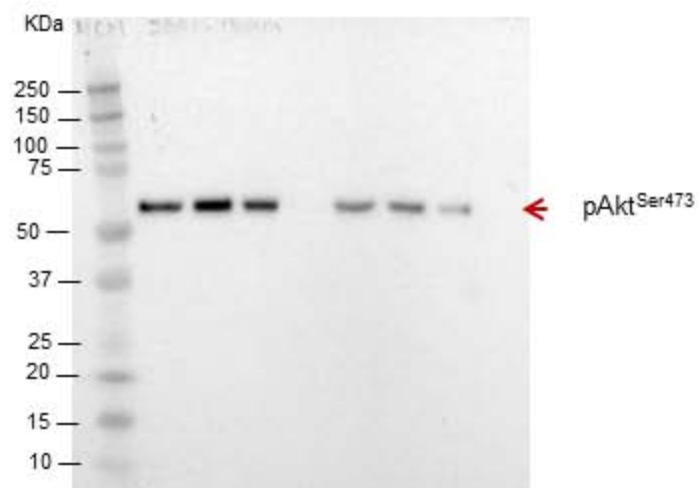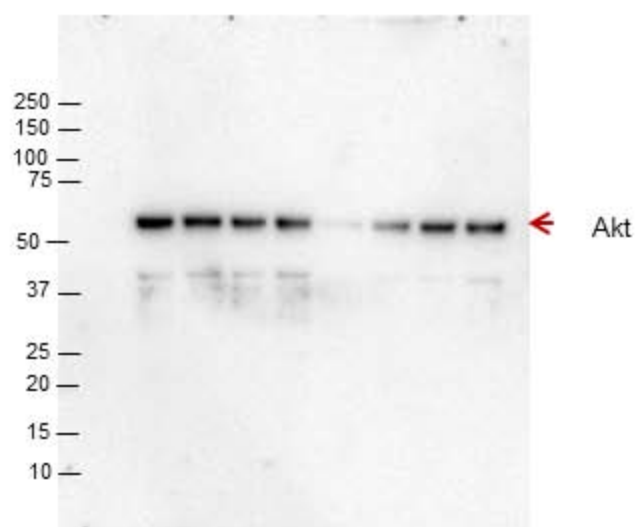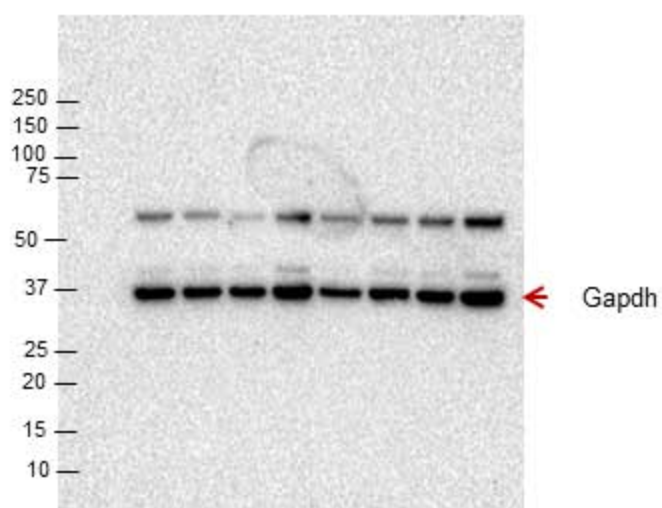

Unprocessed western blots for Figure 6d

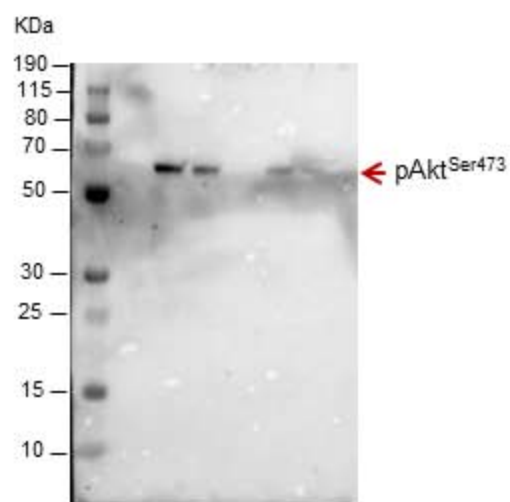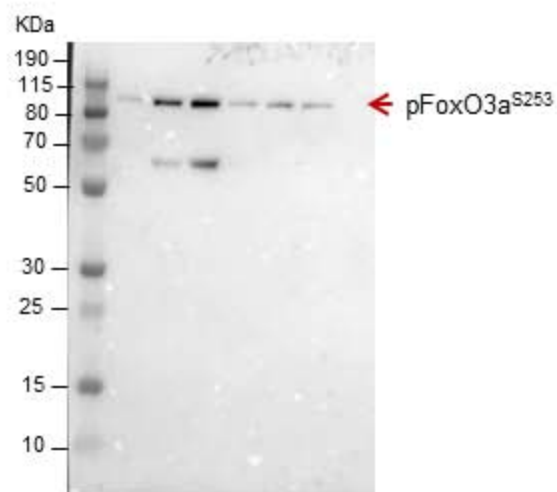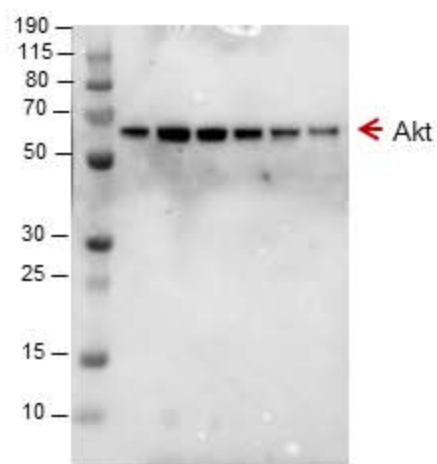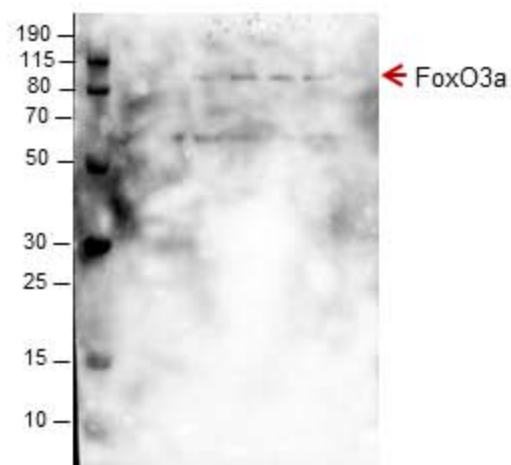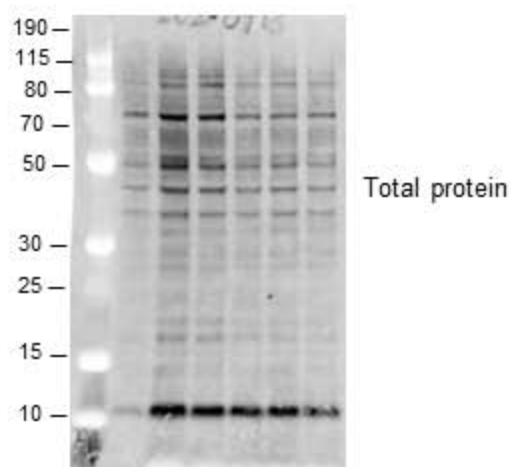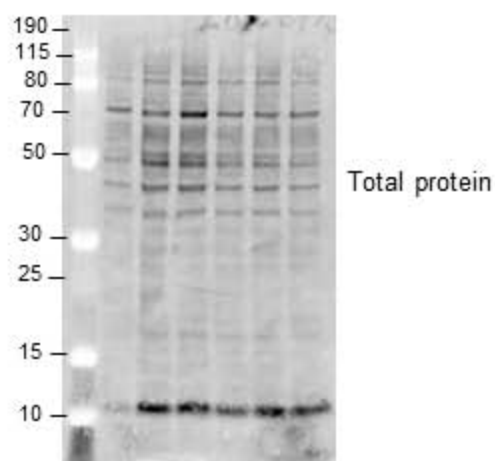

Unprocessed western blots for Figure 6e

Supplement: Supplementary file 7 — Unprocessed western blots for Fig. 6 [file 44161_2022_181_MOESM7_ESM.pdf]

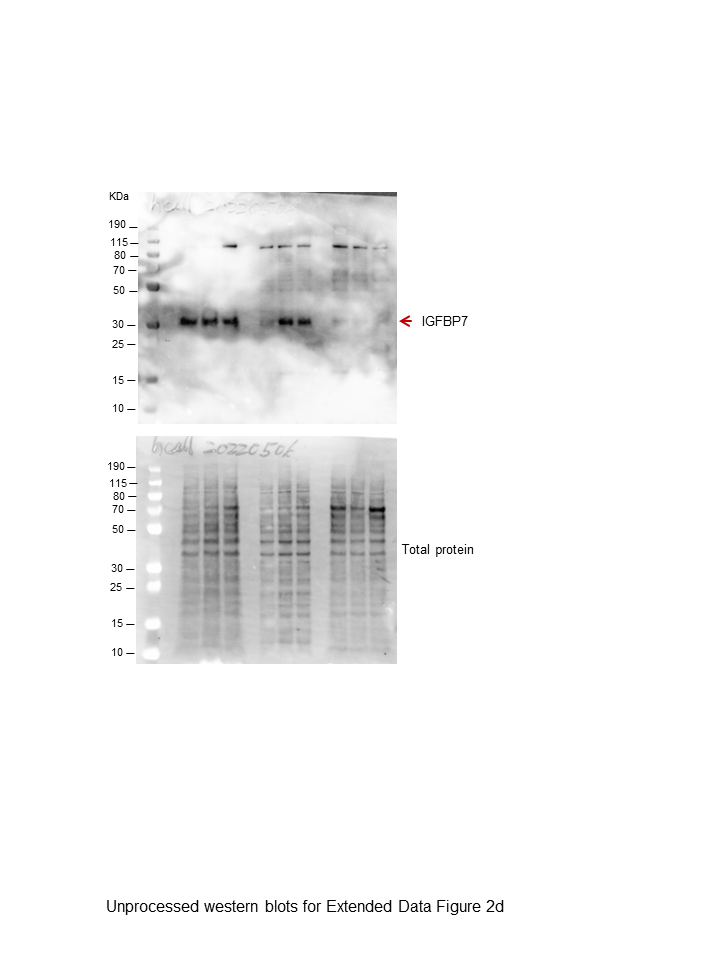

Supplement: Supplementary file 9 — Unprocessed western blots for Extended Data Fig. 2 [file 44161_2022_181_MOESM9_ESM.tif]

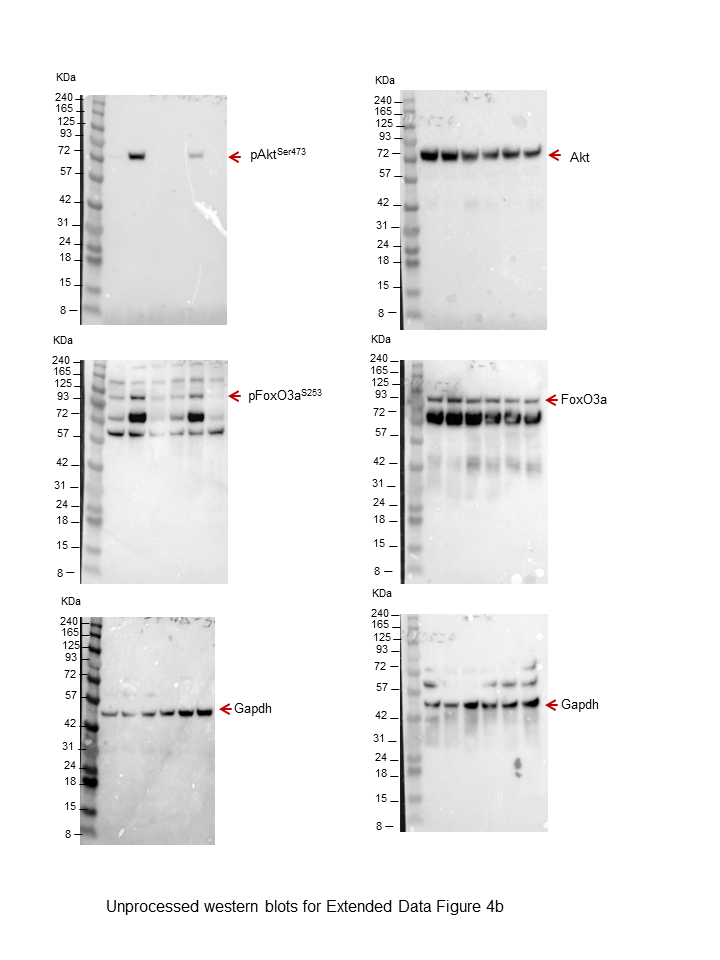

Supplement: Supplementary file 10 — Unprocessed western blots for Extended Data Fig. 4 [file 44161_2022_181_MOESM10_ESM.tif]
