# Supplementary material for: Insulin-like growth factor-binding protein-7 (IGFBP7) links senescence to heart failure
Source: Nat Cardiovasc Res. 2022 Dec 22;1(12):1195–214. doi: 10.1038/s44161-022-00181-y (PMC11358005; doi:10.1038/s44161-022-00181-y)
Supplement: Supplementary file 5 — Unprocessed western blots for Fig. 4 [file 44161_2022_181_MOESM5_ESM.pdf]

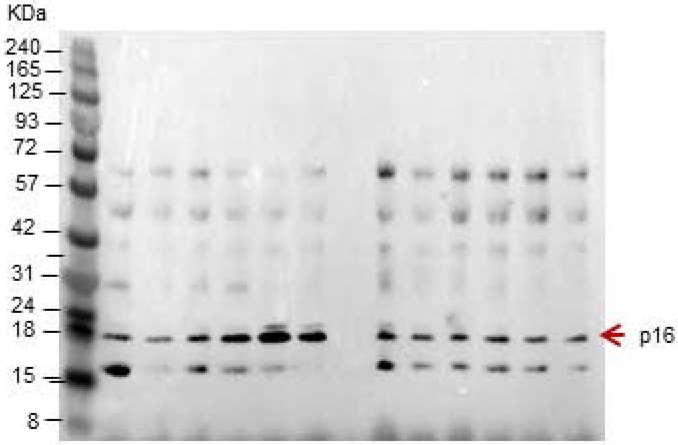

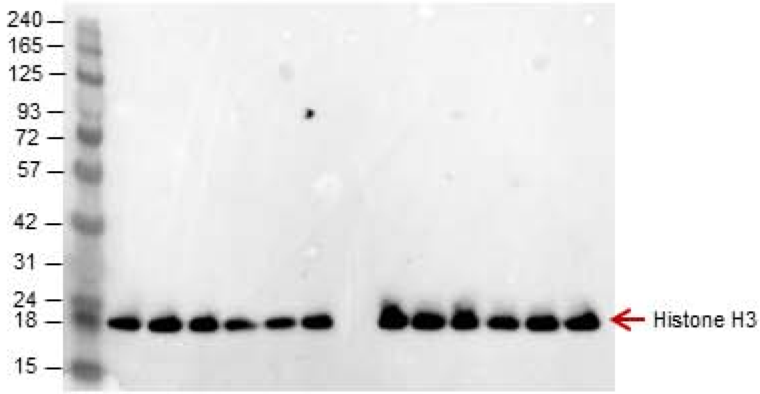

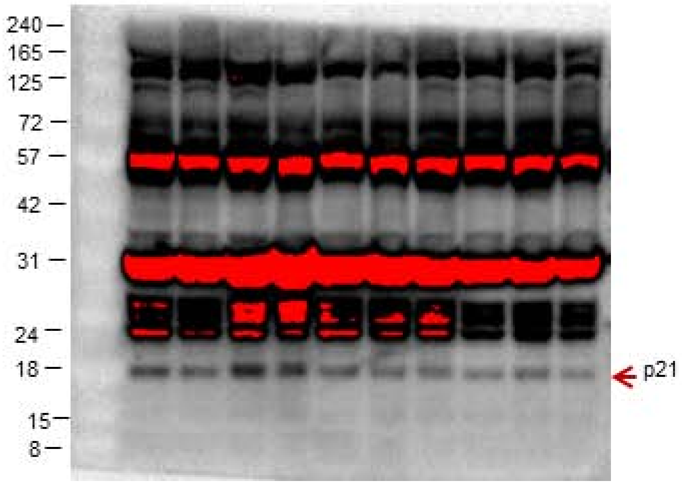

Unprocessed western blots for Figure 4d

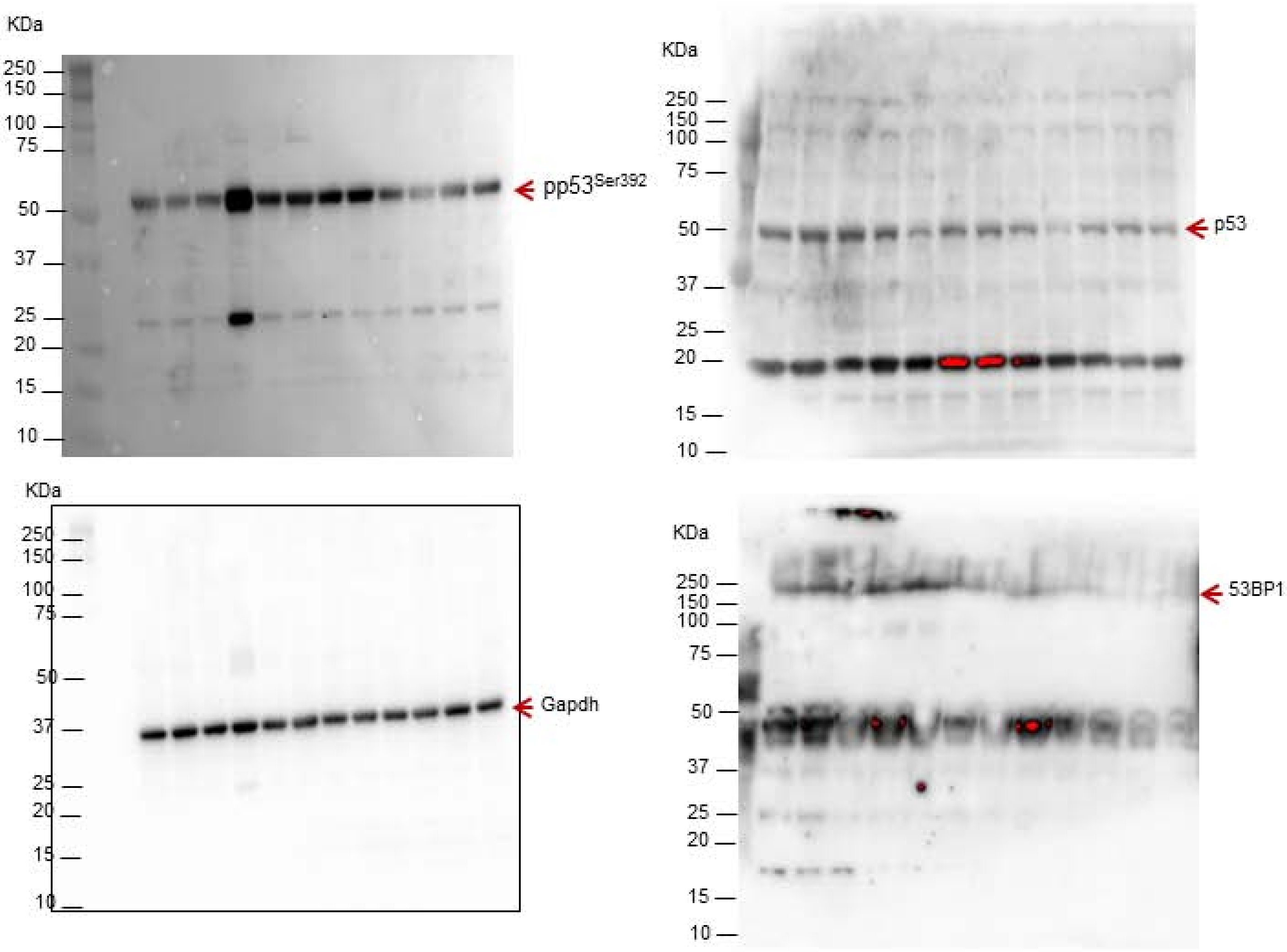

Unprocessed western bots for Figure 4e

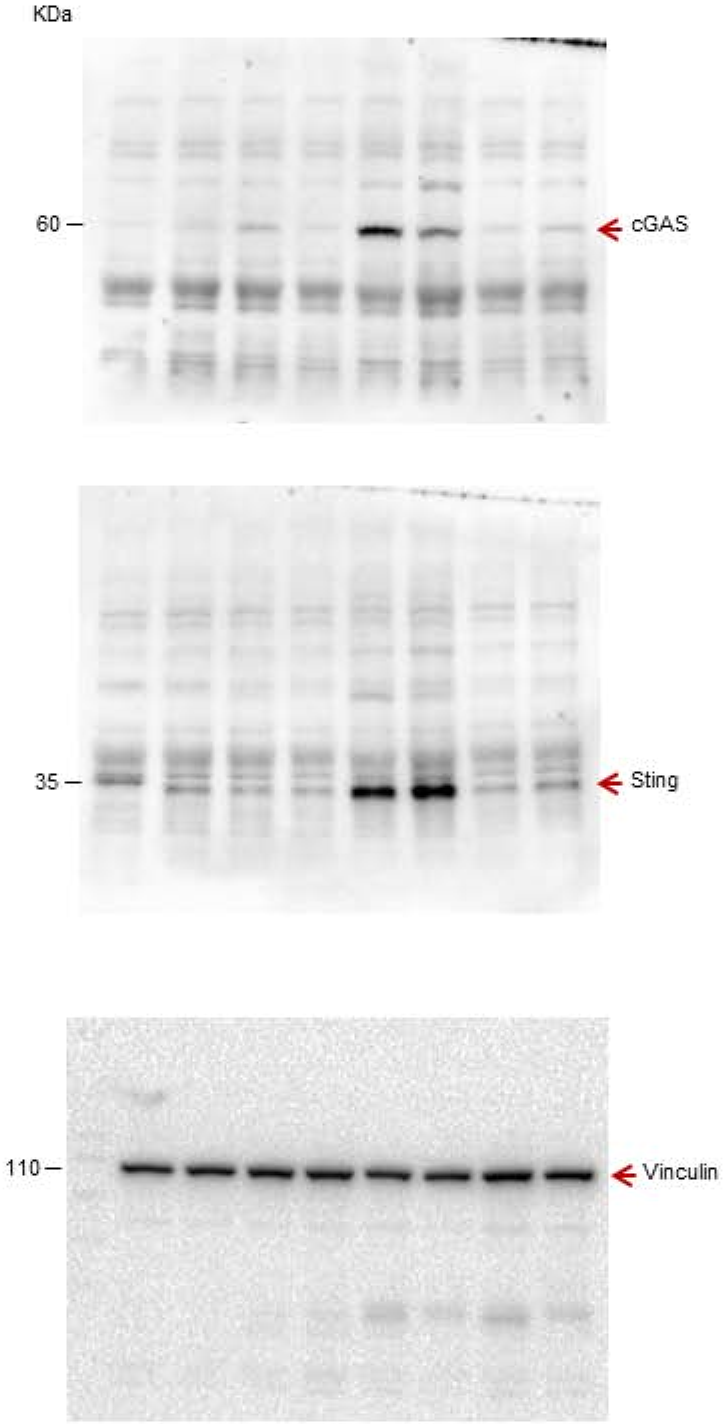

Unprocessed western blots for Figure 4g

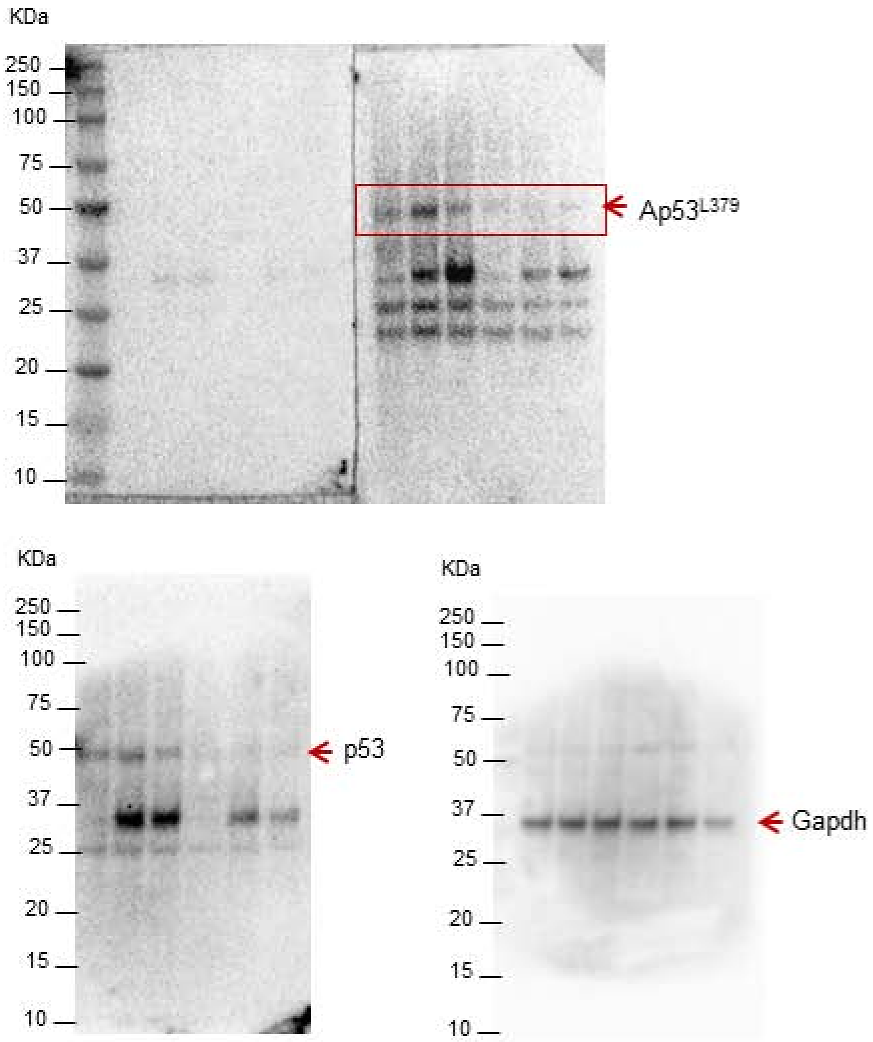

Unprocessed western blots for Figure 4h
