# Supplementary material for: Insulin-like growth factor-binding protein-7 (IGFBP7) links senescence to heart failure
Source: Nat Cardiovasc Res. 2022 Dec 22;1(12):1195–214. doi: 10.1038/s44161-022-00181-y (PMC11358005; doi:10.1038/s44161-022-00181-y)
Supplement: Supplementary file 6 — Unprocessed western blots for Fig. 5 [file 44161_2022_181_MOESM6_ESM.pdf]

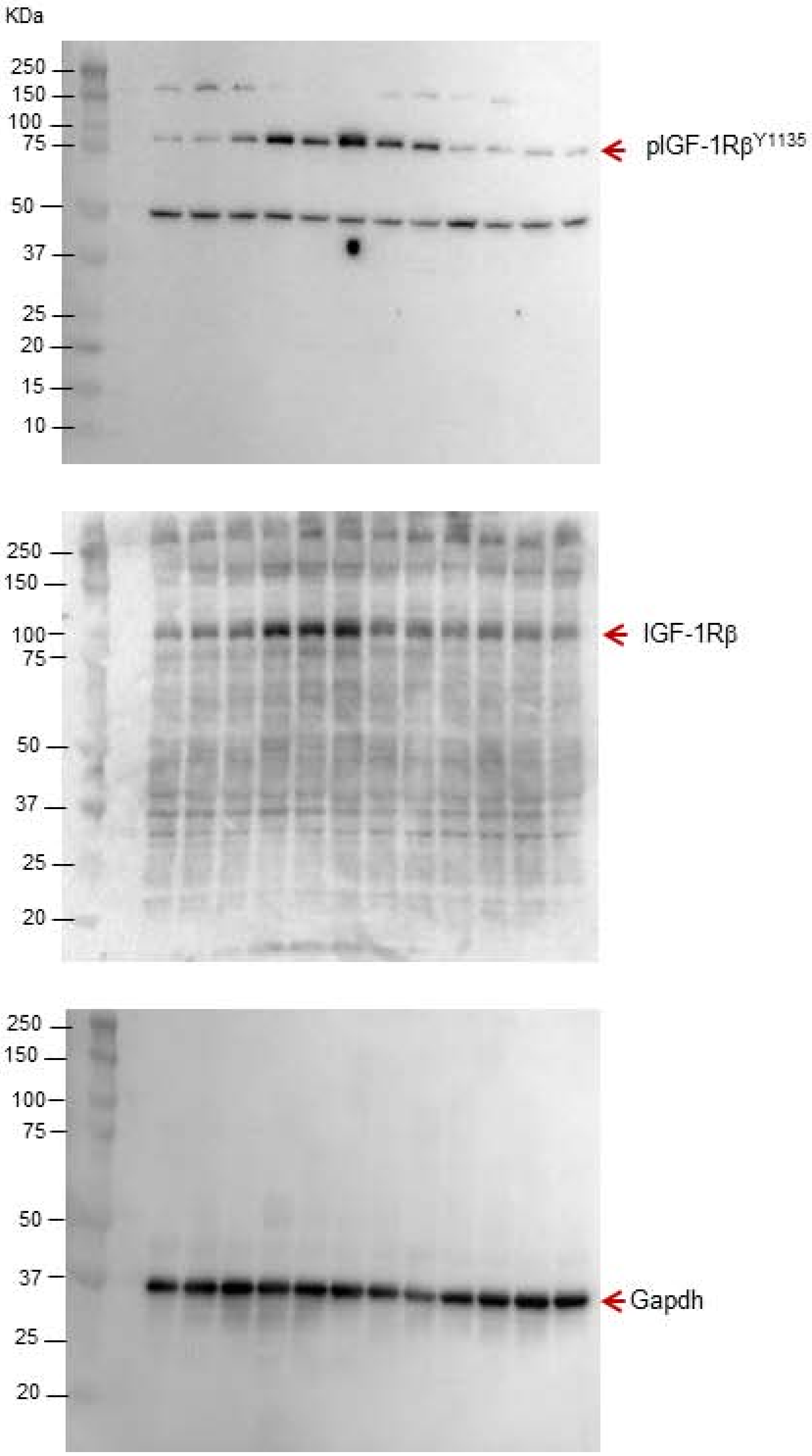

Unprocessed western blots for Figure 5a

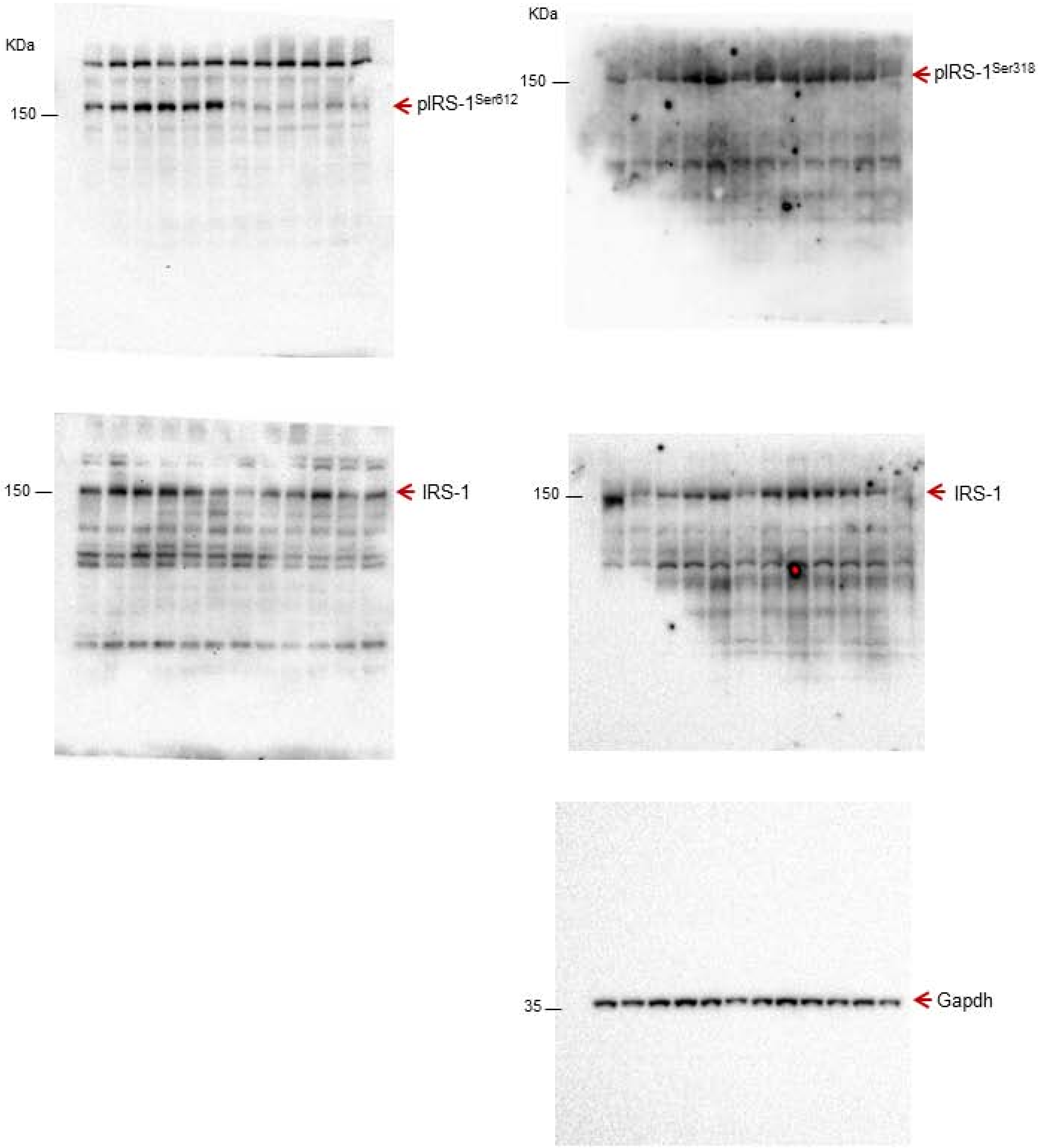

Unprocessed western blots for Figure 5b

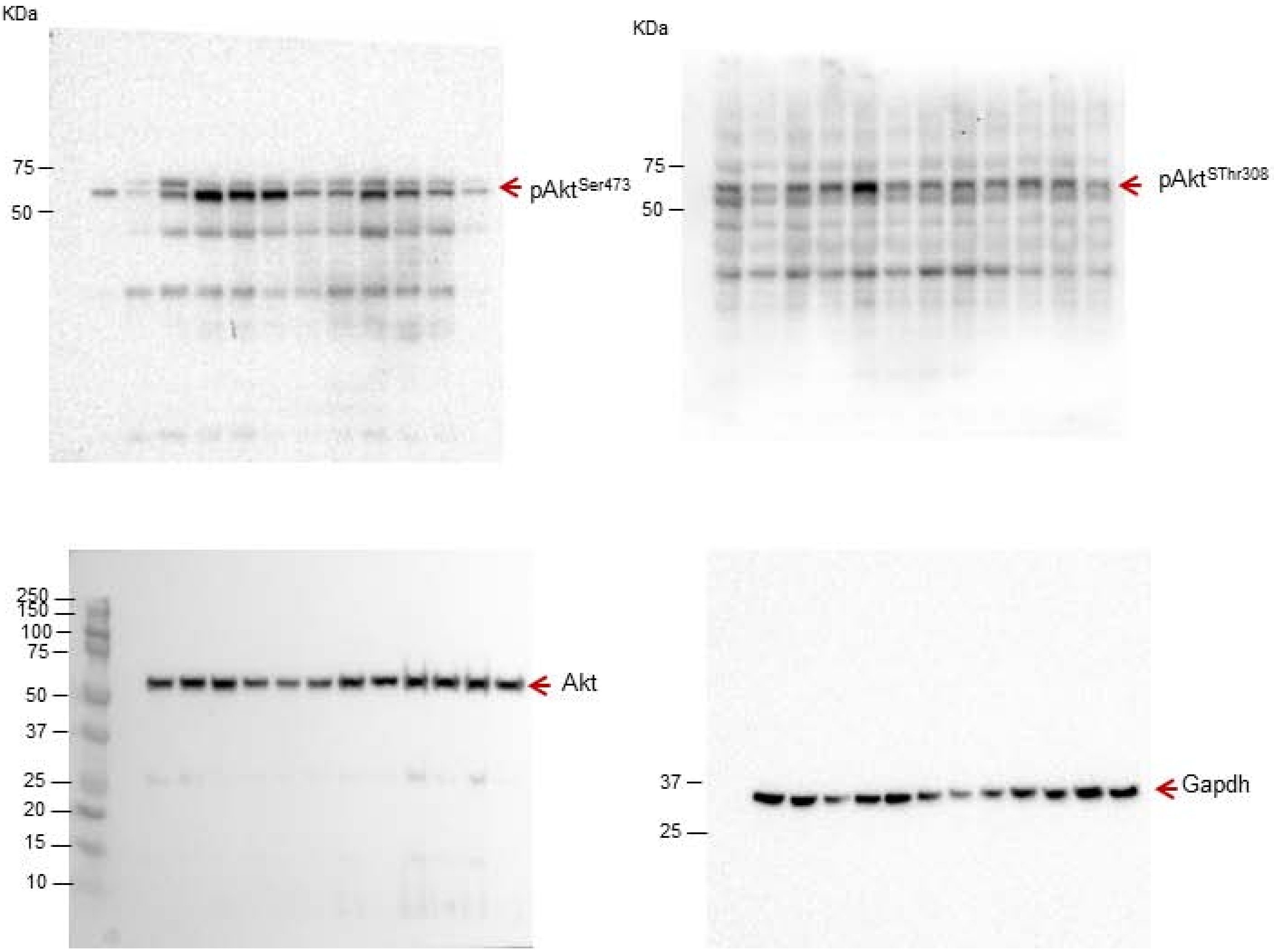

Unprocessed western blots for Figure 5c

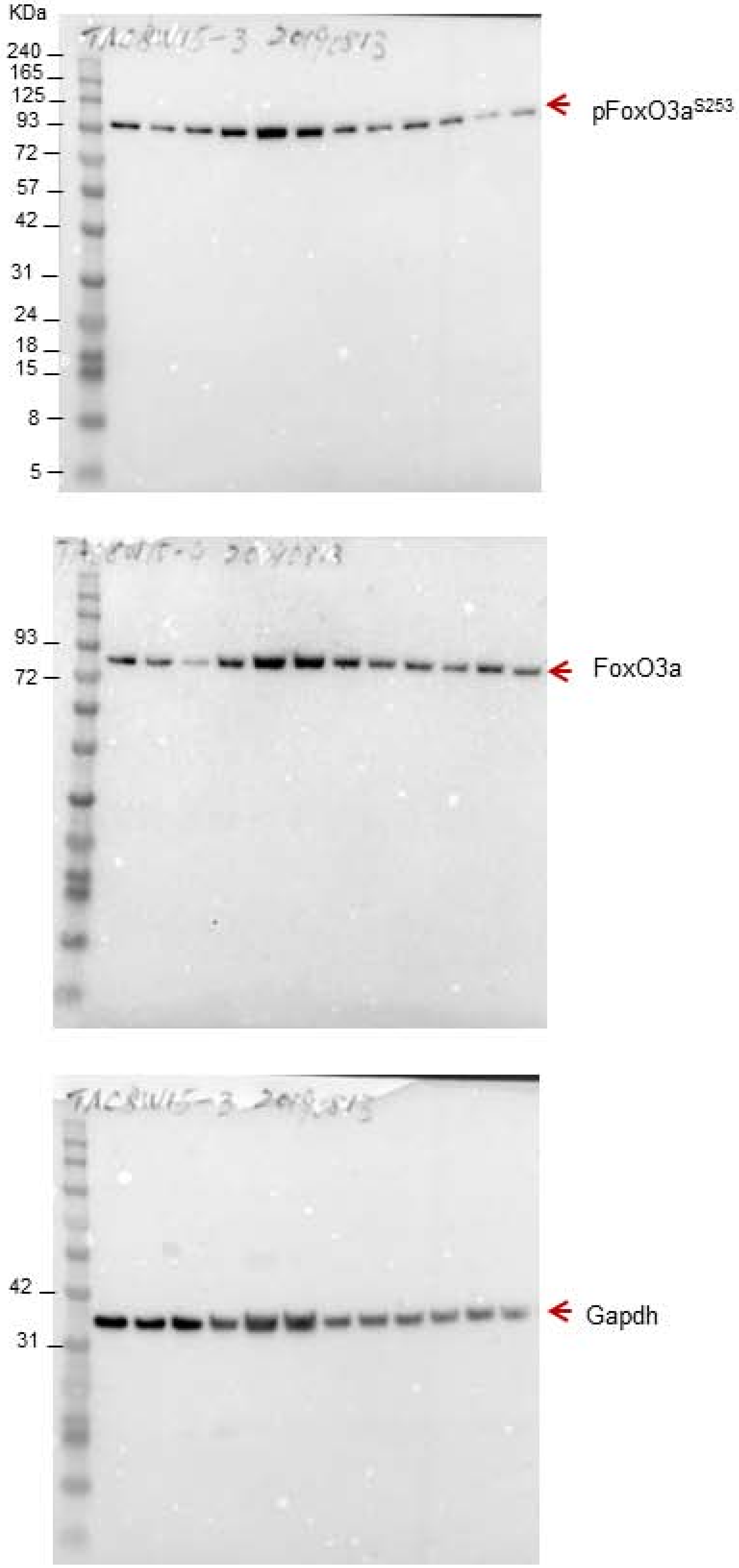

Unprocessed western blots for Figure 5d

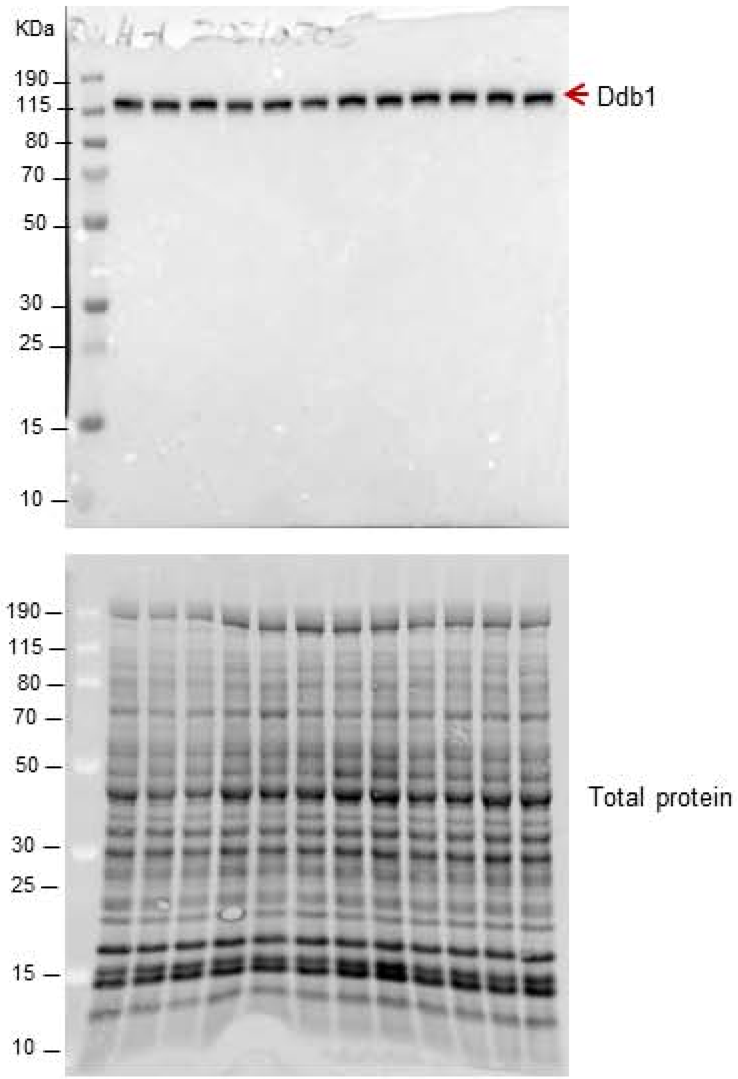

Unprocessed western blots for Figure 5e

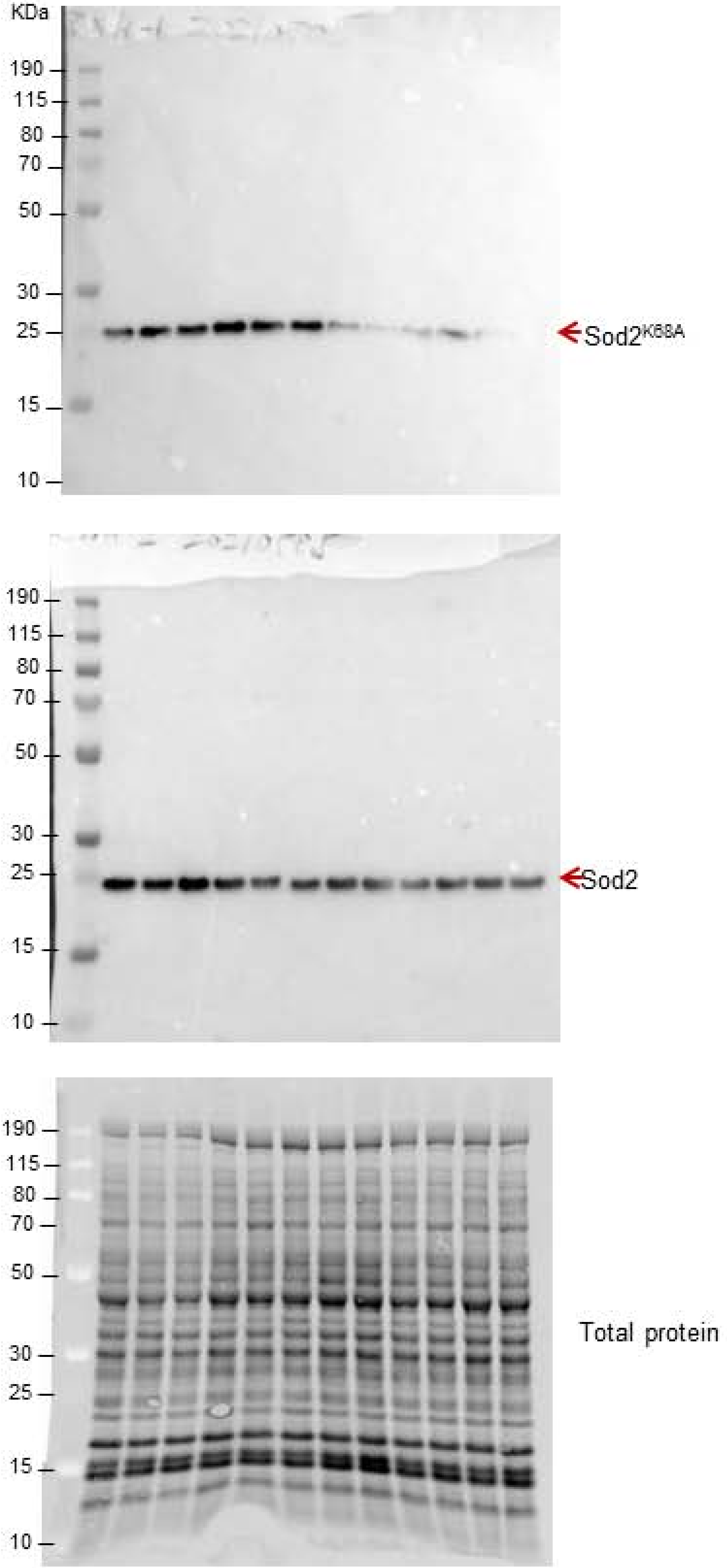

Unprocessed western blots for Figure 5f
