# Supplementary material for: Insulin-like growth factor-binding protein-7 (IGFBP7) links senescence to heart failure
Source: Nat Cardiovasc Res. 2022 Dec 22;1(12):1195–214. doi: 10.1038/s44161-022-00181-y (PMC11358005; doi:10.1038/s44161-022-00181-y)
Supplement: Supplementary file 8 — Unprocessed western blots for Fig. 8 [file 44161_2022_181_MOESM8_ESM.pdf]

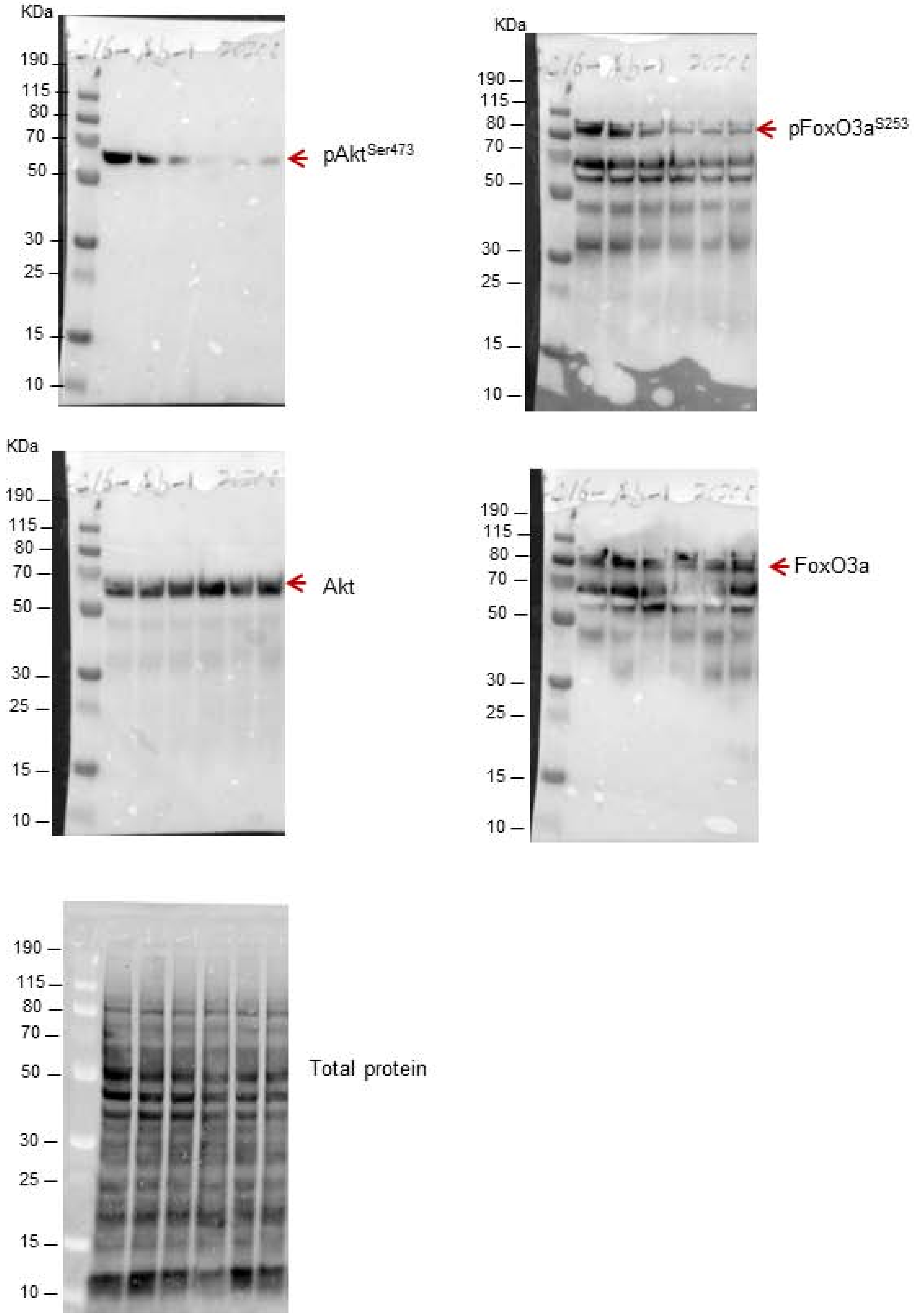

Unprocessed western blots for Figure 8a

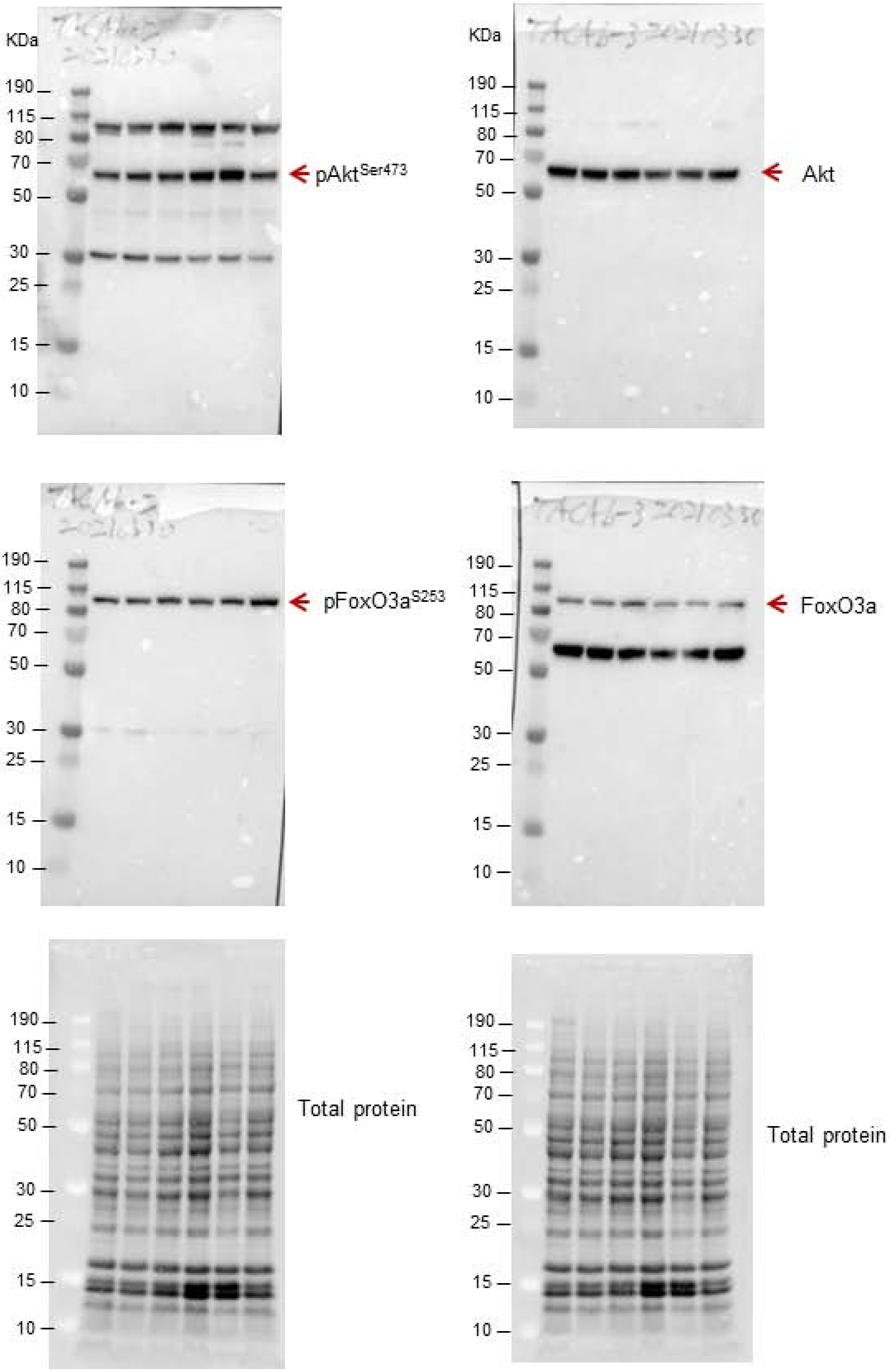

Unprocessed western blots for Figure 8h

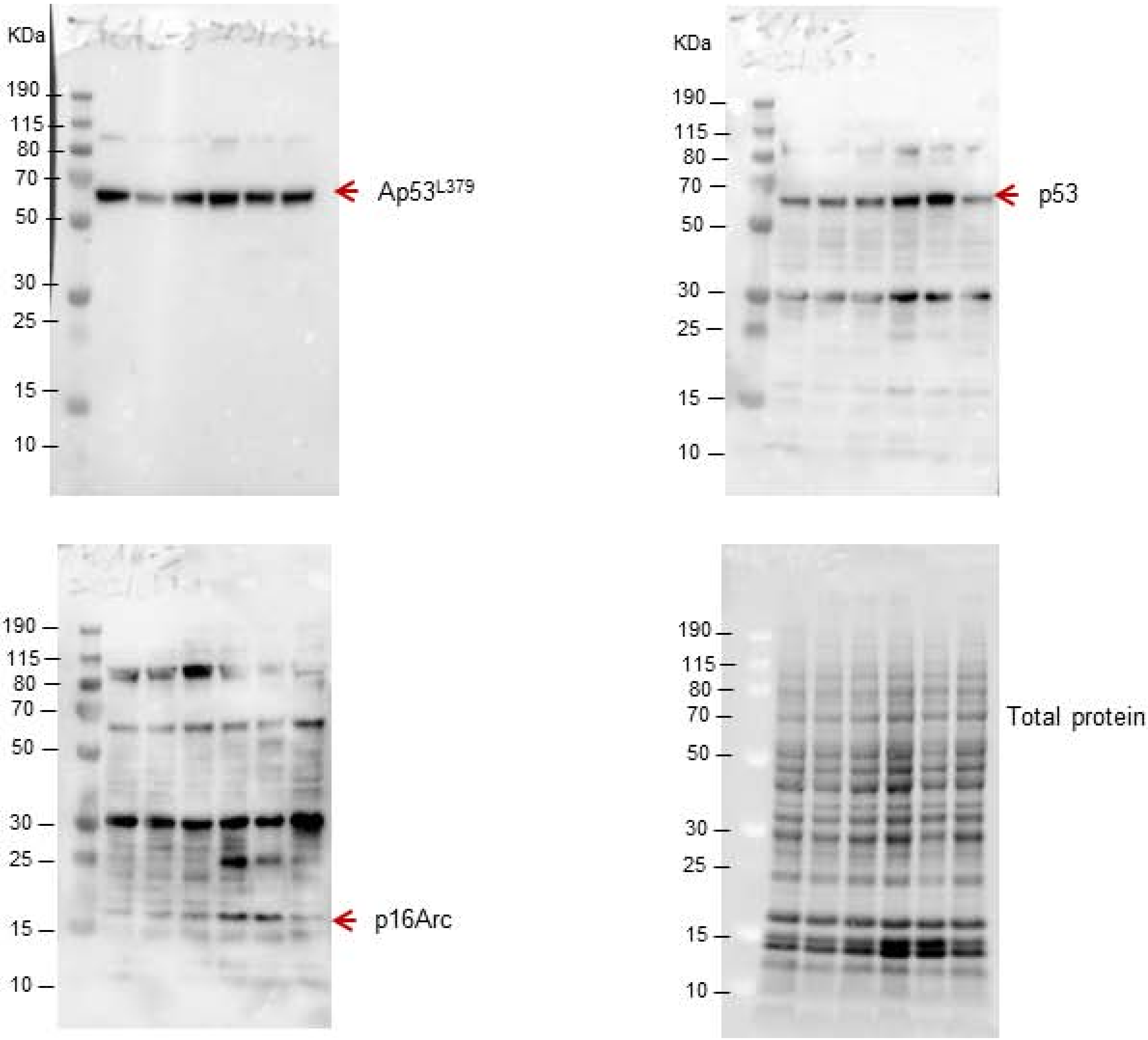

Unprocessed western blots for Figure 8j
