# Supplementary material for: Insulin-like growth factor-binding protein-7 (IGFBP7) links senescence to heart failure
Source: Nat Cardiovasc Res. 2022 Dec 22;1(12):1195–214. doi: 10.1038/s44161-022-00181-y (PMC11358005; doi:10.1038/s44161-022-00181-y)
Supplement: Supplementary file 11 — Unprocessed western blots for Extended Data Fig. 7 [file 44161_2022_181_MOESM11_ESM.pdf]

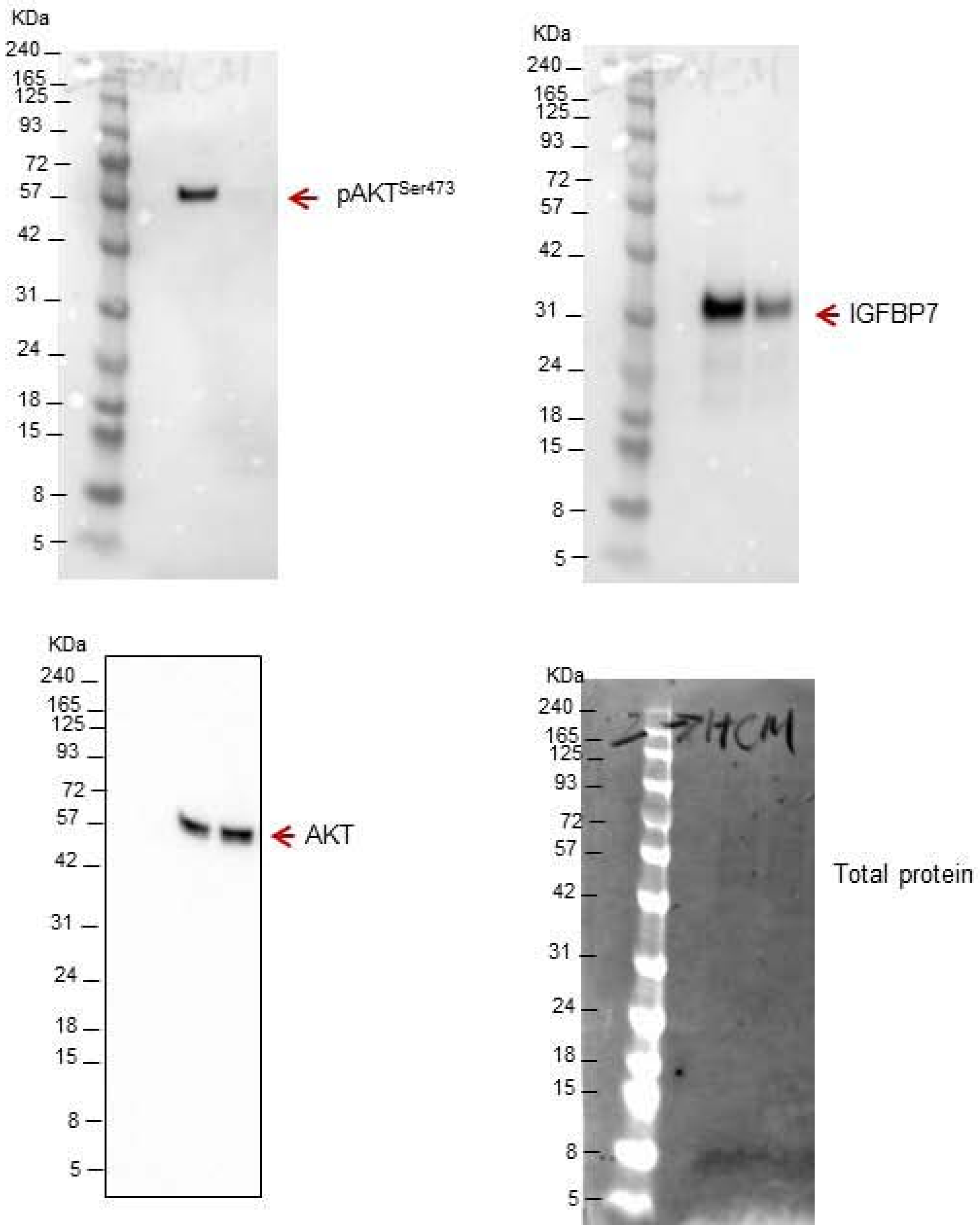

Unprocessed western blots for Extended Data Figure 7b

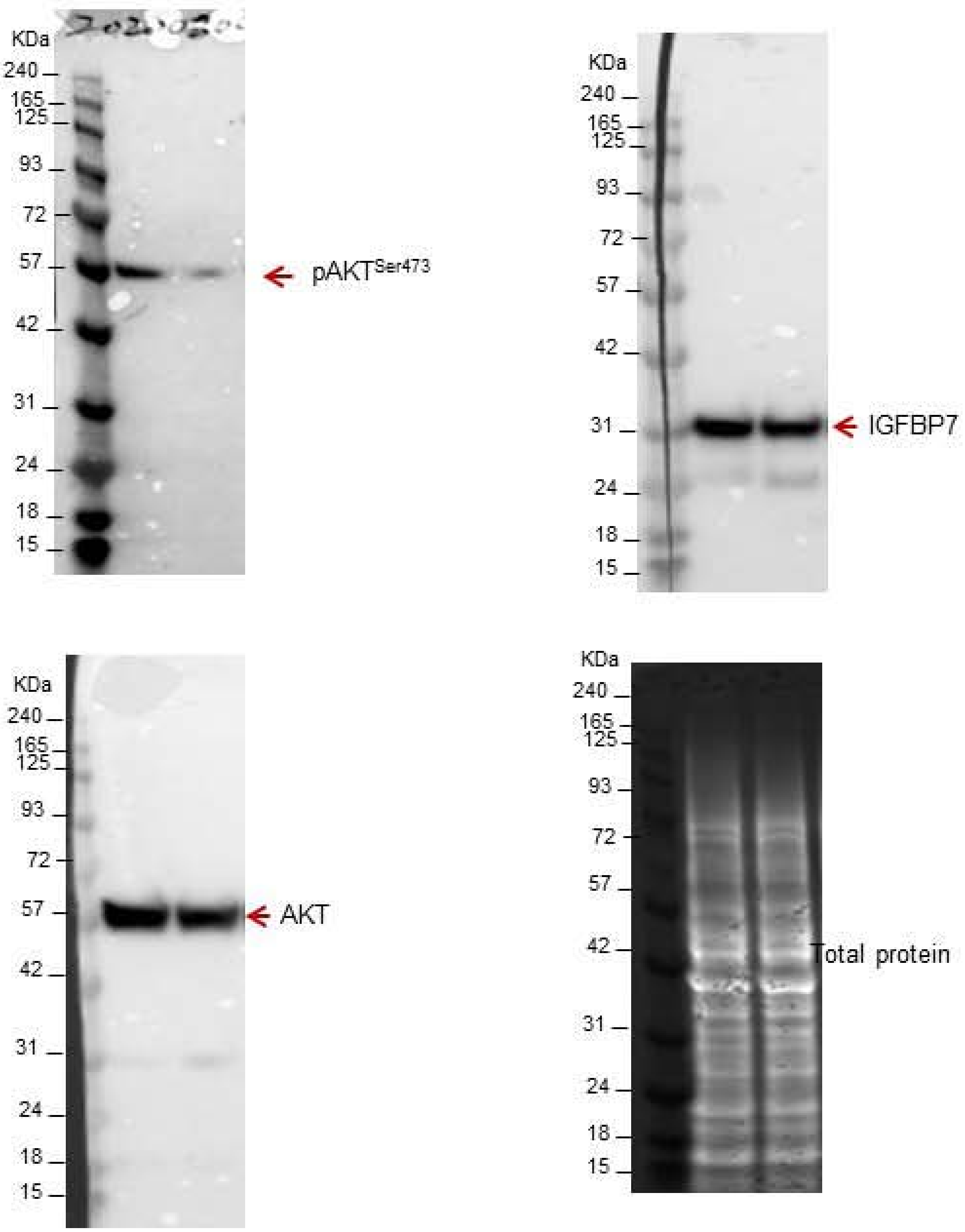

Unprocessed western blots for Extended Data Figure 7c
